# Chinese herbal medicine combined with conventional therapy for gastric precancerous lesions: a systematic review and meta-analysis

**DOI:** 10.3389/or.2026.1947905

**Published:** 2026-10-19

**Authors:** Chang Feng, Jiamin Cao, Lu Zhan, Shujing Yi, Yuanqi Li, Fang Li, Run Yang, Yuantao Zhong, Xingen Jiang, Ruo Chen, Gaofei Feng

**Affiliations:** 1 Graduate School, Guangzhou University of Chinese Medicine, Guangzhou, China; 2 Beijing University of Chinese Medicine Shenzhen Hospital (Longgang) (Shenzhen Longgang District Hospital of Traditional Chinese Medicine), Shenzhen, China; 3 Graduate School, China Academy of Chinese Medical Sciences, Beijing, China; 4 Health Care Department, Guang’anmen Hospital, China Academy of Chinese Medical Sciences, Beijing, China

**Keywords:** Chinese herbal medicine, chronic atrophic gastritis, conventional therapy, dysplasia, gastric precancerous lesions, intestinal metaplasia, meta-analysis, systematic review

## Abstract

**Background:**

Chronic atrophic gastritis and gastric precancerous lesions are associated with increased gastric cancer risk. Chinese herbal medicine is widely used as an adjunct to conventional therapy, but the certainty and clinical interpretation of its effects remain unclear.

**Objective:**

To evaluate the efficacy and safety of Chinese herbal medicine added to the same conventional therapy for chronic atrophic gastritis and gastric precancerous lesions.

**Methods:**

Eight international and Chinese databases and three trial registries were searched from inception to 31 March 2026 and updated to 16 August 2026. Randomised controlled trials comparing Chinese herbal medicine plus the same conventional therapy with conventional therapy alone and reporting extractable pathological outcomes were included. Random-effects models used restricted maximum likelihood estimation and Hartung-Knapp inference. Additional sensitivity analyses examined clinically comparable histopathology, baseline balance based on auditable pretreatment data, and exclusion of high-risk-of-bias results. Risk of bias was assessed using RoB 2 and certainty using GRADE.

**Results:**

Sixty-nine randomised trials comprising 6,983 participants in eligible comparisons were included. Exploratory Broad end-of-treatment analyses favoured combined treatment for gastric mucosal atrophy (30 studies; SMD −1.63, 95% CI −2.03 to −1.24), intestinal metaplasia (29 studies; SMD −1.48, 95% CI −1.90 to −1.06), and dysplasia or intraepithelial neoplasia (21 studies; SMD −1.25, 95% CI −1.51 to −0.99). Heterogeneity was substantial (I^2^ 82.4%–92.7%); prediction intervals crossed the null for atrophy and intestinal metaplasia but not for dysplasia/intraepithelial neoplasia. Sensitivity analyses restricted to clinically comparable histopathological assessments included only three to four studies and were imprecise, with confidence intervals crossing the null. *Helicobacter pylori* clearance/negative conversion among baseline-positive participants was higher with combined treatment (15 studies; RR 1.25, 95% CI 1.15–1.37), whereas the strict test-of-cure sensitivity was imprecise (3 studies; RR 1.19, 95% CI 0.91–1.56). There was no clear evidence of a difference in reported adverse events (14-study evidence set; RR-contributing studies = 12; RR 0.93, 95% CI 0.58–1.50), which should not be interpreted as evidence of equivalent safety. Certainty was low for all five principal outcomes.

**Conclusion:**

Chinese herbal medicine added to conventional therapy may be associated with lower short-term pathological scores on average, but the magnitude and clinical meaning of these effects remain uncertain because of trial limitations, marked heterogeneity, and imprecision in clinically comparable analyses. The findings do not establish durable histological regression or gastric cancer prevention, and the pooled estimates should not be interpreted as a class-wide effect applicable to all Chinese herbal medicines.

**Systematic Review Registration:**

https://www.crd.york.ac.uk/PROSPERO/view/CRD420261442138, identifier CRD420261442138.

## 1 Introduction

Gastric cancer remains a major global health burden, with more than 968,000 new cases and nearly 660,000 deaths estimated in 2022, ranking fifth worldwide for both cancer incidence and mortality (1). Most non-cardia intestinal-type gastric cancers develop through a multistep sequence of chronic gastritis, gastric mucosal atrophy, intestinal metaplasia, dysplasia, and invasive carcinoma. These precursor conditions are clinically important because the risk of progression increases with histological severity. A recent global meta-analysis estimated gastric-cancer progression rates of 2.09, 2.89, and 10.09 per 1,000 person-years for atrophic gastritis, intestinal metaplasia, and dysplasia, respectively (2). Although progression is not inevitable, identifying and managing these lesions provides an important opportunity for gastric-cancer risk reduction.

Current management is centred on identifying and eradicating *Helicobacter pylori* and performing high-quality endoscopic examination with appropriate biopsy sampling. Accurate characterisation of the extent and severity of atrophy and intestinal metaplasia informs risk-stratified surveillance (3–6). Histological staging systems such as OLGA and OLGIM can assist risk stratification, while visible dysplastic lesions require expert endoscopic assessment and treatment (3, 5, 6). *Helicobacter pylori* eradication can heal non-atrophic gastritis, may promote regression of atrophy, and reduces gastric-cancer risk; nevertheless, patients with established extensive atrophy, intestinal metaplasia, or dysplasia may retain residual risk and may continue to require surveillance (3–6). Beyond eradication and the endoscopic management of neoplasia, evidence for medical interventions that reliably reverse established histological lesions or prevent their long-term progression remains limited.

Chinese herbal medicine is widely used in China as an adjunct to conventional therapy for chronic atrophic gastritis and gastric precancerous lesions. Proposed therapeutic actions include protection of the gastric mucosal barrier, modulation of inflammatory and immune responses, improvement of microcirculation, and regulation of oxidative stress, cell proliferation, and apoptosis (7, 8). Chinese integrated guidance considers traditional Chinese medicine and integrated Chinese-conventional treatment potentially beneficial, but also emphasises that the evidence base is constrained by the scarcity of large, multicentre, placebo-controlled trials with standardised pathological assessment and long-term follow-up (7). Accordingly, the clinical value of adding Chinese herbal medicine to established conventional therapy management requires rigorous synthesis focused on objective pathological outcomes rather than mechanistic plausibility or symptom response alone.

Several systematic reviews have reported favourable effects of traditional Chinese medicine on chronic atrophic gastritis or gastric precancerous lesions, but important uncertainties remain. A 2020 review primarily evaluated study-defined clinical efficacy, *H. pylori* eradication, endoscopic response, traditional Chinese medicine syndrome response, and individual symptoms, rather than separately synthesising continuous scores for atrophy, intestinal metaplasia, and dysplasia (9). A subsequent review of Weifuchun included 15 studies but was restricted to a single proprietary medicine and identified substantial limitations in trial design, heterogeneous treatment duration, and the absence of long-term follow-up (10). Another review combined a meta-analysis of 12 randomised trials with network pharmacology and molecular docking, addressing a broader mechanistic question rather than providing a pathology-centred assessment of the incremental effect of adjunctive Chinese herbal medicine (11). More generally, previous reviews often combined heterogeneous herbal interventions or comparators, relied heavily on non-standardised composite response rates, and did not simultaneously separate the three principal pathological lesions, quantify the expected range of effects using prediction intervals, and integrate result-specific RoB 2 assessments with outcome-level GRADE judgements.

We therefore conducted a systematic review and meta-analysis of randomised controlled trials to evaluate the incremental efficacy and safety of Chinese herbal medicine added to the same conventional therapy regimen for patients with chronic atrophic gastritis or gastric precancerous lesions. We required extractable pathological data and separately synthesised post-treatment scores for gastric mucosal atrophy, intestinal metaplasia, and dysplasia or intraepithelial neoplasia. We also distinguished objective pathological outcomes from study-defined clinical response, symptom, endoscopic, biomarker, *H. pylori*, and safety outcomes, and evaluated the robustness and certainty of the evidence using random-effects models, prediction intervals, sensitivity analyses, RoB 2, and GRADE.

## 2 Methods

### 2.1 Protocol and registration

This review was not prospectively registered before the original search cutoff. The original database search ended on 31 March 2026. The published PROSPERO record CRD420261442138 reports a review start date of 1 March 2026, first submission on 5 July 2026, and registration on 9 July 2026. At registration, formal searching/study identification was recorded as completed and screening as started, whereas data extraction, risk-of-bias assessment, and data synthesis were recorded as not started. PROSPERO also states that no separate full protocol had been written; accordingly, the published registration record was used as the reference for the registration-to-final-method comparison. The core population, intervention, and randomised-study eligibility framework was retained, while comparator wording, pathological outcome architecture, model choice, small-study-effect testing, and reviewer-driven robustness analyses were clarified or refined during revision. These differences are documented in Supplementary Table SR10 and are not described as prespecified. The review is reported in accordance with PRISMA 2020 (12).

### 2.2 Eligibility criteria

Studies were eligible according to the PICOS criteria specified for this review. Participants had chronic atrophic gastritis or gastric precancerous lesions, including intestinal metaplasia, low-grade dysplasia, or low-grade intraepithelial neoplasia, diagnosed using recognised criteria. Eligibility was not restricted by *H. pylori* status or traditional Chinese medicine syndrome differentiation. For mixed populations, data for the eligible population had to be separately extractable.

The intervention was Chinese herbal medicine added to conventional therapy and the comparator was the same conventional therapy alone. Chinese herbal medicine could be a fixed or modified formula in a clinically used preparation. Studies were eligible only when the incremental effect attributable to adding Chinese herbal medicine could be isolated. At least one extractable pathological outcome was required, and only randomised controlled trials were eligible.

Multiple reports from the same or substantially overlapping participant population were linked and counted as one independent study. Complementary reports were used only for non-overlapping information. Multi-arm trials were counted once at study level and shared controls were handled to prevent double-counting.

### 2.3 Information sources and search strategy

The original search covered PubMed, Embase, the Cochrane Library, Web of Science, CNKI, Wanfang Data, VIP, and SinoMed, together with ClinicalTrials.gov, WHO ICTRP, and ChiCTR, from inception to 31 March 2026. Reference lists of included studies and relevant reviews were also screened. On 16 August 2026, all eight databases were rerun using the same search concepts, and ClinicalTrials.gov, WHO ICTRP, and ChiCTR were re-searched without date or recruitment-status restrictions. No language restrictions were applied to study eligibility.

Complete original and update search strategies, platform-specific syntax adaptations, and search audit notes are provided in Supplementary Appendix S1.

### 2.4 Study selection and eligibility audit

C.F. and J.C. independently screened titles and abstracts. Any record considered potentially eligible by either reviewer proceeded to full-text assessment. The same two reviewers independently assessed full texts against the predefined PICOS criteria and recorded one primary reason for exclusion for each assessed full-text report. Disagreements were resolved by discussion and, when necessary, adjudication by G.F. Multiple reports from the same or substantially overlapping population were linked before study-level counting.

A structured full-text eligibility re-assessment was conducted using the same PICOS criteria and source-level verification. Reports previously included or excluded were rechecked under the unchanged criteria. For the 26 reports recorded as not retrieved in the historical screening snapshot, title- and author-based searches were repeated in CNKI, Wanfang Data, and VIP. Because the 26 full texts were still not directly accessible, original-article requests were submitted through Wanfang Library document delivery; 15 reports were obtained through this route and assessed. For the remaining 11 reports, additional searches of the relevant journal official websites and historical journal archives were undertaken, but the full texts remained unavailable. The original title/abstract screening counts were retained because individual historical screening decisions could not be reliably reconstructed from later working fields, and unsupported reasons for earlier omissions were not inferred.

### 2.5 Data extraction and source verification

Data were extracted independently by C.F. and J.C. using a predesigned standardised form at study, comparison, and result level. Discrepancies were resolved by discussion and, when necessary, adjudication by G.F.; the completed dataset was additionally checked by L.Z. Extracted information included randomised and analysed denominators, pathology methods and biopsy information, intervention reproducibility fields, *H. pylori* denominators and testing details, and result-specific RoB 2 evidence. When discrepancies were identified between prior extraction records and the source report, source-verified full-text data were used. Information not documented in the source was retained as not reported; no clinical assumptions or numerical imputations were used to fill undocumented fields.

For continuous pathological outcomes, source-reported post-treatment means, standard deviations, and result-specific denominators were used. Source-reported mean changes or baseline-adjusted estimates would have been analysed separately, but no lesion-specific study provided a directly usable mean-change with SDchange or an ANCOVA/baseline-adjusted effect. We therefore did not derive change-score variances from baseline and follow-up standard deviations or assume within-participant correlations.

### 2.6 Outcome definitions and pathology comparability architecture

The three principal pathological outcomes were lesion-specific end-of-treatment scores for gastric mucosal atrophy, intestinal metaplasia, and dysplasia/intraepithelial neoplasia, analysed separately. Lower scores indicated less severe pathological changes; negative SMDs favoured combined treatment. Overall/composite histopathology, endoscopic-pathological composites, clinical composites, and lesion-specific outcomes were not treated as interchangeable.

For eligibility as a continuous lesion-specific pathological outcome, the source had to report a numerical or ordinal severity measure explicitly representing gastric mucosal atrophy, intestinal metaplasia, or dysplasia/intraepithelial neoplasia at the eligible end-of-treatment time point, with directly usable arm-level summary data and denominators. No specific histopathological grading system was mandatory for the Broad analysis. Source-defined lesion-severity scores were therefore eligible even when reporting of the biopsy site or sampling protocol was incomplete, whereas the Restricted analysis required explicit biopsy-derived histopathology with interpretable anatomical sampling, scale, time point, and denominator. OLGA/OLGIM staging was not an eligibility requirement and, when reported, was analysed separately from lesion-specific continuous scores.

Pathology evidence was organised into three levels. Level 1 (Broad) comprised eligible lesion-specific end-of-treatment scores across heterogeneous measurement systems and was interpreted as an exploratory average rather than evidence of a clinically uniform or class-wide effect. Contributing sources used ordinal 0-3 or 0/1/2/3 grades, 0/3/6/9 grades, and source-defined or multi-item totals; score direction was harmonised so that lower values indicated less severe pathology. Sampling descriptions ranged from explicit antrum/body/angulus or multi-site biopsy to incompletely reported sites. Level 2 (Restricted clinically comparable histopathology) required explicit biopsy-derived lesion-specific pathology, interpretable anatomical sampling, a clear scale, time point and denominator, and no severe unexplained scale or construct anomaly. Level 3 comprised non-comparable pathological evidence summarised separately. Because the available reporting did not establish full clinical interchangeability, clinical interpretation gives particular weight to the uncertainty of the Restricted evidence.

The following result-selection hierarchy was applied: (1) use the eligible end-of-treatment time point; (2) keep atrophy, intestinal metaplasia, and dysplasia/IEN as separate lesion constructs; (3) when a source identified an overall lesion value, use that value rather than selecting the most favourable site; (4) when a source explicitly designated a primary anatomical site or scale, use that designation; and (5) when no defensible single choice existed, preserve all eligible candidate results in the audit and apply the shared-study/shared-control construction without selecting by effect size or statistical significance. Anatomical-site and scale criteria were used only to define the Restricted sensitivity. The study-level implementation is documented in Supplementary Data S7.

The baseline-balanced sensitivity required directly auditable pretreatment mean and SD values on the same lesion-specific construct and scale as the end-of-treatment outcome with clear denominators. An author-reported statement of baseline P > 0.05 alone was insufficient. No arbitrary numerical threshold for baseline SMD was imposed.

Clinically interpretable binary histological outcomes were kept separate from continuous pathological scores. Pure lesion-specific histological improvement was not combined with lesion disappearance/normalisation, endoscopy-plus-pathology responses, clinico-endoscopic-pathological composites, multi-lesion composite responses, or denominator-conflicted outcomes. Sparse OLGA/OLGIM and stage-transition evidence was summarised separately. Study-level pathology measurement, biopsy-site/protocol, baseline-comparability, and outcome-selection details are provided in Supplementary Data S7.

Study-defined clinical total effective rate was treated as a non-principal secondary composite outcome. Definitions and response thresholds were not standardised across all contributing trials; each trial’s source-reported response definition, numerator, and denominator were retained rather than imposing a common threshold retrospectively. This outcome was therefore interpreted as a heterogeneous study-defined clinical response measure and not as an interchangeable measure of histological regression.

### 2.7 *Helicobacter pylori* and safety outcomes

The broad *H. pylori* outcome was defined as clearance/negative conversion among participants explicitly positive at baseline. An additional anti-H. pylori-regimen subset and a stricter test-of-cure sensitivity were examined separately. The strict subset required a baseline-positive denominator; a guideline-accepted follow-up test (urea breath, faecal antigen, or biopsy-based testing); assessment at least 4 weeks after completion of *H. pylori* therapy; and source-documented medication washout sufficient to reduce false-negative testing (PPIs/PCABs for at least 2 weeks and antibiotics/bismuth for at least 4 weeks) (5, 13). Unsupported timing or washout assumptions were not permitted. Post-treatment negative status in mixed baseline populations, outcomes lacking a baseline-positive denominator, continuous infection grades, and studies without adequate timing documentation were not included in the strict subset. Study-level baseline-positive denominators, baseline and follow-up diagnostic methods, timing, washout information, eradication regimens, events, and final strict test-of-cure classification are provided in Supplementary Data S8.

For safety, quantitative synthesis required explicit arm-specific event counts and denominators. The broad reported-adverse-event analysis included all studies with eligible quantitative event data, with a clinical-adverse-event-only sensitivity excluding a composite safety outcome that also incorporated laboratory/organ-function abnormalities. Qualitative safety statements were summarised narratively and were not converted to zero events. Double-zero trials were retained in the complementary RD sensitivity.

### 2.8 Risk of bias assessment

RoB 2 (22 August 2019 version) was applied at result/outcome-family level for the effect of assignment to intervention (14). Five domains were assessed: randomisation process, deviations from intended interventions, missing outcome data, outcome measurement, and selection of the reported result. Overall judgements followed the RoB 2 algorithms. For D4, lack of reported assessor blinding for subjective pathological scoring led to Some concerns when knowledge of intervention could plausibly influence assessment; High risk required evidence that such influence was likely or another RoB 2 high-risk condition. S20 was High overall because of D1 evidence from strong, directionally consistent baseline pathological imbalances and inconsistent baseline-comparability reporting; its D4 judgement remained Some concerns. High-risk results were retained in the Broad synthesis and excluded in a sensitivity analysis. Any algorithm-guided judgement or documented override is shown with its signalling responses and rationale in Supplementary Data S3.

### 2.9 Statistical analysis

For continuous outcomes, MDs were used when the same meaningful scale was shared and bias-corrected Hedges g SMDs when conceptually equivalent constructs were measured on different scales. SMD was not used to legitimise pooling of different constructs. Dichotomous outcomes were summarised as RRs. Random-effects models used restricted maximum likelihood estimation of tau-squared and Hartung-Knapp inference with an *ad hoc* safeguard that did not allow the Hartung-Knapp standard error to be smaller than the conventional random-effects standard error; 95% prediction intervals were reported when estimable (15–17). For binary principal outcomes, approximate numbers needed to treat for benefit or harm were derived from the pooled observed control-group risk and pooled RR when the confidence interval for the absolute effect remained on one side of no difference; no single NNT/NNH was reported when the confidence interval included both benefit and harm.

Eligible treatment arms sharing a comparator were combined when clinically appropriate, with the shared control counted once. Common-effect Mantel-Haenszel models were model-comparison sensitivities for dichotomous outcomes only. Adverse-event RD sensitivity retained double-zero trials. Outcome mappings and sensitivity definitions were applied consistently across the final dataset.

### 2.10 Heterogeneity, subgroup, meta-regression, and sensitivity analyses

Clinical and methodological heterogeneity was evaluated using participant characteristics, disease subtype, *H. pylori* status, herbal regimen, conventional-therapy comparator, treatment duration, pathology system, biopsy method, outcome definition, and time point. Statistical heterogeneity was described using Q, I-squared, tau-squared, and prediction intervals.

Subgroup factors were pathological scoring system, treatment duration (<=8 vs. > 8 weeks), broad conventional-therapy comparator, disease subtype, and *H. pylori* status. Two additional exploratory dimensions were explicit TCM syndrome-differentiation reporting and conventional-therapy functional category. Formal interaction testing required at least three independent studies in each compared category. Interaction tests used mixed-effects meta-regression with a common residual tau-squared and Knapp-Hartung omnibus tests; Benjamini-Hochberg adjustment was applied separately across the 20 estimable subgroup interaction tests and across the eight estimable univariable meta-regression tests. Analyses that did not meet the minimum-size or variation requirements were not included in the corresponding multiplicity family.

Univariable meta-regression was performed only when at least 10 studies had reliable covariate data and meaningful variation. The retained covariates were treatment duration (coefficient per 4-week increase), broad conventional-therapy comparator, and *H. pylori* status. No multivariable model was fitted. Biopsy protocol and fixed versus modified herbal therapy were not formally modelled because missing or conflated reporting would have caused substantial misclassification.

Sensitivity analyses comprised the Restricted histopathology set, the baseline-balanced set, exclusion of High-RoB results, leave-one-out analysis, the extreme-effect analysis (absolute SMD >4), *H. pylori* regimen/test-of-cure restrictions, clinical-adverse-event-only analysis, adverse-event RD analysis, and model-comparison analyses. The Broad synthesis was interpreted as an exploratory average across heterogeneous measurement systems rather than evidence of a clinically uniform class effect; the Restricted and other sensitivity analyses assessed clinical comparability and robustness.

### 2.11 Reporting bias and small-study effects

For syntheses with at least 10 statistically independent contributing comparisons, formal asymmetry tests were considered (18). For SMD syntheses with archived study-level asymmetry inputs, funnel-plot asymmetry was assessed using the Pustejovsky-Rodgers regression test, which is designed to avoid the artifactual association between an SMD and its standard error that can affect conventional Egger regression (19). RR outcomes used the Harbord modified score test (20). For non-principal secondary SMD outcomes, the archived dataset did not preserve the study-level inputs required for corrected SMD-specific asymmetry testing; therefore, no replacement formal asymmetry P values are reported for those outcomes. Contour-enhanced funnel plots were generated for the five principal outcomes and are provided in Supplementary Data S5. Two-sided P < 0.10 was treated as suggestive of small-study effects; neither the presence nor absence of statistical asymmetry was equated with publication bias, and findings were interpreted with heterogeneity, study size, trial methods, and availability of registered or unpublished evidence (18). Archived R scripts, principal study-level inputs, and validation targets are provided in Supplementary Data S6. Exact software versions used for the earlier archived analyses were not preserved. The final method-corrected Pustejovsky-Rodgers analyses and the corrected dysplasia/IEN baseline-balanced sensitivity were independently rerun and verified in R 4.5.2 with meta version 8.2-1; sessionInfo(), validation logs, analysis inputs, and outputs are archived in Supplementary Data S6.

### 2.12 Certainty of evidence

GRADE certainty was assessed for the five principal outcomes in the Summary of Findings table: atrophy, intestinal metaplasia, dysplasia/IEN, *H. pylori* clearance/negative conversion, and reported adverse events (15, 21). RoB 2 and GRADE judgements were reviewed by C.F. and J.C., with discrepancies resolved by discussion and adjudication by G.F. when needed. Randomised evidence started at high certainty and could be downgraded for risk of bias, inconsistency, indirectness, imprecision, and publication/dissemination bias. Overlapping concerns were not double-counted. For the principal pathological outcomes, differences in measurement systems and biopsy implementation were addressed primarily under inconsistency and through the Restricted clinical-comparability sensitivity; indirectness was not downgraded separately because the target outcome was lesion-specific histopathological severity itself. The same GRADE domains were applied separately to the 23 non-principal secondary outcomes; those ratings did not replace the principal-outcome GRADE judgements in the main Summary of Findings table.

During preparation of this manuscript, ChatGPT (GPT-5.5 Thinking and GPT-5.6 Thinking, OpenAI) and Codex (GPT-5.6 Sol, OpenAI) were used for language editing, manuscript organisation, selected drafting and revision, and R-code development and checking. All study eligibility decisions, source verification, analyses, statistical outputs, and final interpretations were independently reviewed and verified by the authors, who take full responsibility for the work.

## 3 Results

### 3.1 Study selection

The final integrated PRISMA 2020 flow combined the original and update searches, which identified 3,080 database records and 15 registry records (3,095 total). After 1,265 duplicates were removed, 1,830 records were screened and 1,267 were excluded at title/abstract stage. Full texts of 563 reports were sought; 11 reports remained unavailable after the additional retrieval attempts described above, leaving 552 reports assessed for eligibility. A total of 483 full-text reports were excluded: 454 historical/original exclusions after re-assessment using the same PICOS criteria, 15 later-retrieved historical reports that were assessed and found ineligible, and 14 reports excluded from the 2026 update search. The final review included 69 independent randomised studies. For audit transparency, the historical screening snapshot is reported separately: 26 reports had originally been recorded as not retrieved; 15 were subsequently obtained through Wanfang Library document delivery and assessed, whereas 11 remained unavailable despite subsequent journal-website and historical-archive searches.

Full-text eligibility re-assessment using the same PICOS criteria identified 67 eligible historical/original studies: 47 retained from the initially included 52-study set and 20 previously missed eligible studies. Accordingly, 454 of the 521 historically assessed full-text reports were ineligible after the eligibility audit. Five studies from the initially included set were removed because their eligible comparisons did not satisfy the PICOS criteria.

The updated search through 16 August 2026 yielded 28 additional records, corresponding to 16 unique reports after deduplication. Fourteen were excluded and two newly eligible independent studies were added. The 468-participant WW-1 trial identified in the update (91) was excluded because it compared WW-1 with matched placebo rather than Chinese herbal medicine added to the same conventional-therapy background versus that background alone. The final review therefore comprised 69 independent randomised studies. The selection process and audit trail are shown in Figure 1 and Supplementary Table SR1. Report‐level full‐text exclusion reasons are provided in Supplementary Data S1.

**FIGURE 1:**
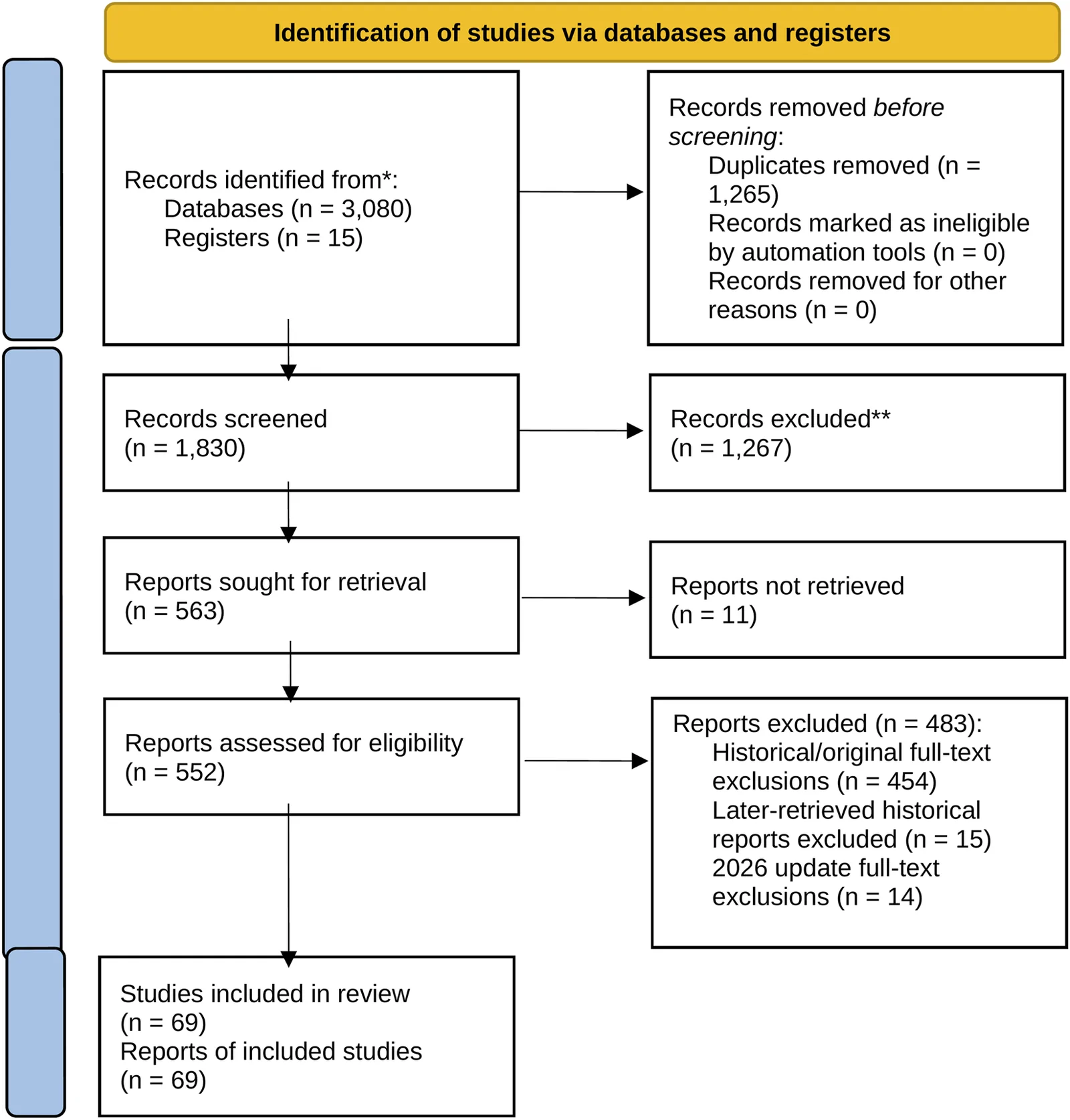
PRISMA 2020 flow diagram and eligibility audit. The final integrated flow identified 3,080 database records and 15 registry records (3,095 total); after removal of 1,265 duplicates, 1,830 records were screened and 1,267 were excluded at title/abstract stage. Full texts of 563 reports were sought; 11 remained unavailable after additional retrieval attempts, and 552 were assessed. Full-text exclusions totalled 483: 454 historical/original exclusions after re-assessment using the same PICOS criteria, 15 later-retrieved historical reports assessed and found ineligible, and 14 update-search exclusions. The final review included 69 independent randomised studies. Historical retrieval audit: 26 reports had been recorded as not retrieved in the original screening snapshot; 15 were subsequently obtained and assessed, and 11 remained unavailable. Adapted from Page MJ et al. BMJ. 2021;372:n71, under the CC BY 4.0 license.

### 3.2 Characteristics of the final evidence base

The 69 included studies were published between 2003 and 2026 and represented 6,983 randomised participants across the eligible study comparisons after shared controls were counted once (22–63), (64–90). The final evidence base comprised 47 studies retained from the initially included set, 20 eligible studies identified during full-text re-assessment, and two studies from the 2026 update. Treatment duration ranged from 10 days to 12 months where explicitly reported. Herbal interventions and conventional-therapy backgrounds were clinically diverse; source-reported formulation, preparation, duration, manufacturer/quality-control information, and modification status are provided in Supplementary Table SR2, with NR retained when information was not reported. All 69 included trials were conducted in China.

Detailed intervention reproducibility was incomplete in many reports. Chinese ingredient names and quantities were transcribed from the source reports. For botanical identification, the corresponding botanical/Latin names in Supplementary Table S1 were editorially standardised from the source-reported Chinese ingredient terms using the 2025 Chinese Pharmacopoeia and accepted taxonomic nomenclature; they were not necessarily printed verbatim in the original reports. Where a Chinese term did not identify a unique species, a genus-level designation or ‘species not specified’ was used. No undocumented formula component, dose, manufacturer, batch, or quality-control information was reconstructed. Table 1 summarises the final evidence base; detailed characteristics of all 69 studies are provided in Supplementary Tables S1A–E.

**TABLE 1:** Summary characteristics of the final 69-study evidence base.

Characteristic	Final value	Comment
Independent studies	69	47 retained from the initially included set + 20 identified during reassessment + 2 update-search studies
Randomised participants in eligible comparisons	6,983	Shared control groups counted once
Publication years	2003–2026	Based on included-study source audit
Historical screening snapshot	1,814 records screened; 521 reports assessed	Original screening-stage counts retained for audit transparency
Historical/original eligible set after full-text reassessment	67 studies	47 retained + 20 identified; same PICOS criteria
2026 update search	28 records -> 16 unique reports -> 14 excluded -> 2 included	Search updated through 16 August 2026
Primary pathology contributors	Atrophy k = 30; IM k = 29; dysplasia/IEN k = 21	Exploratory broad end-of-treatment analyses
Treatment duration	10 days to 12 months where reported	Substantial clinical variation
Intervention reproducibility	Variable; many fields NR	Detailed source-reported information in Supplementary Table SR2

Detailed characteristics of all 69 independent studies are provided in Supplementary Tables S1A–E. Source-reported intervention-reproducibility information is provided in Supplementary Table SR2 and Supplementary Data S2. Participant totals refer to eligible study comparisons with shared controls counted once. NR, not reported.

### 3.3 Risk of bias

RoB 2 was evaluated at result/outcome-family level. Among the 31 studies contributing to at least one Broad continuous lesion-specific pathology synthesis, 30 were judged to have some concerns overall and S20 was judged High risk for its atrophy and intestinal-metaplasia results because of multiple strong, directionally consistent baseline pathological imbalances coupled with inconsistent reporting of baseline comparability. Excluding S20 did not materially change either pooled estimate. Across this pathology family, allocation concealment, deviations/adherence reporting, assessor blinding, and prospective outcome-selection safeguards were commonly insufficiently reported; missing-data concerns were concentrated in a small number of studies. The distribution of RoB 2 judgements for the 31 study-outcome-family assessments contributing to the primary continuous lesion-specific pathology family is shown in Figure 2. Supplementary Data S3 provides complete signalling-question responses, domain judgements, source locators, and rationales for all 102 final result/outcome-family assessments.

**FIGURE 2:**
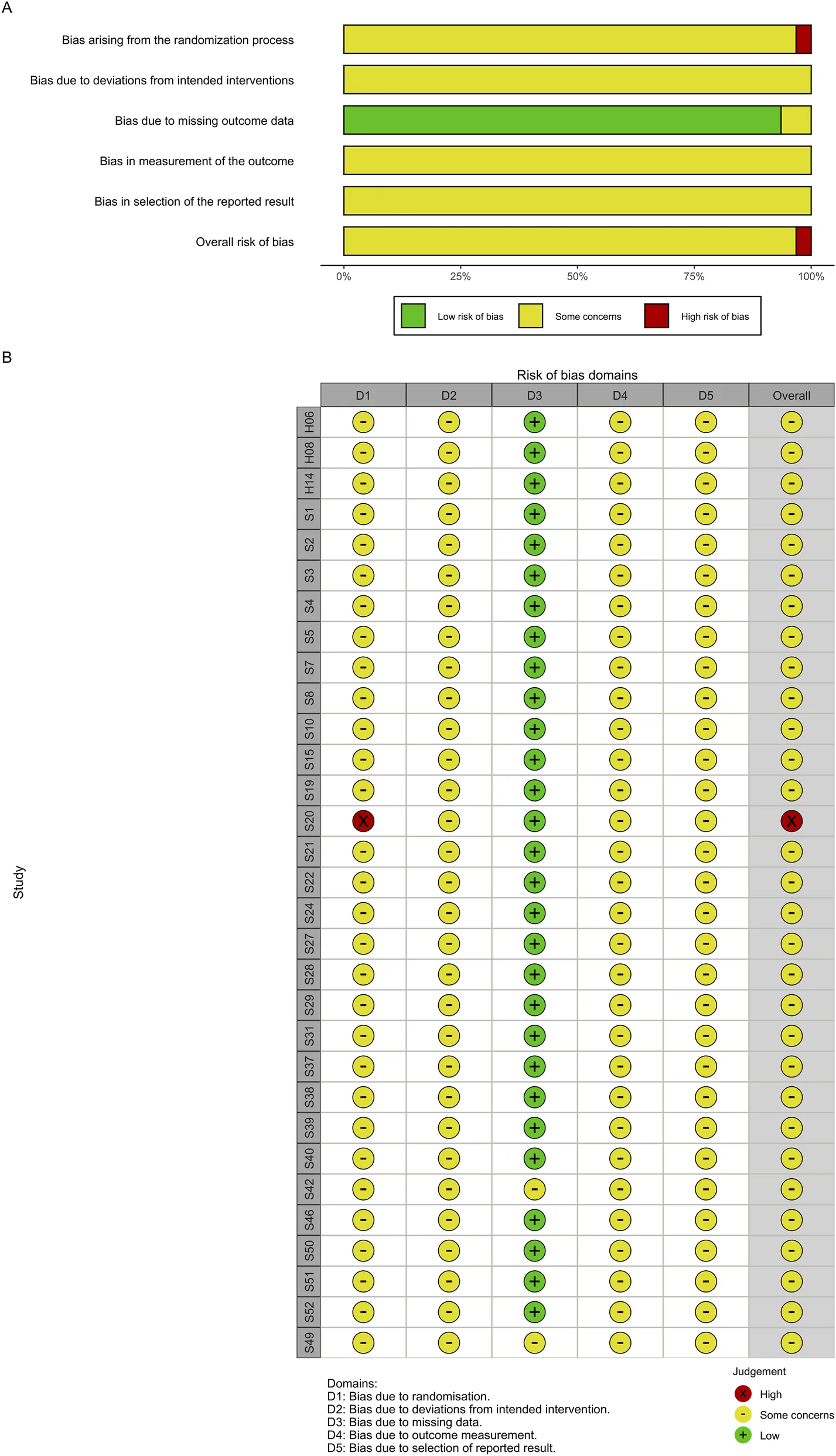
RoB 2 distribution for the 31 study‐outcome‐family assessments contributing to the primary continuous lesion‐specific pathology family (30 some concerns; 1 high risk). **(A)** Distribution of risk‐of‐bias judgements across the five RoB 2 domains and the overall judgement. **(B)** Traffic‐light plot of individual study‐outcome‐family assessments by domain and overall judgement. RoB 2 targeted the effect of assignment to intervention. The complete signalling‐question audit of all 102 final result/outcome‐family assessments is provided in Supplementary Data S3. S20 was judged high risk for its atrophy and intestinal‐metaplasia results; all other assessments in this 31‐assessment figure had some concerns overall. D1, randomisation process; D2, deviations from intended interventions; D3, missing outcome data; D4, outcome measurement; D5, selection of the reported result.

The companion denominator audit reports randomised, completed, analysed, pathology-specific, *H. pylori*, and safety denominators for all 69 studies. Fourteen studies required study-specific documentation or adjudication of denominators, attrition, or analysis populations, and 55 of 69 reports did not name the analysis population as ITT, FAS, PPS, or completer. These source-supported classifications informed D2 and D3; no completer or per-protocol result was relabelled as intention-to-treat (Supplementary Data S4).

### 3.4 Primary lesion-specific pathological outcomes

The final pathology audit comprised 80 lesion-specific effects contributing to the three principal pathological outcomes: 61 met the baseline-balanced rule and 19 did not. S20 was the only study with clearly strong printed baseline imbalances affecting both atrophy and intestinal-metaplasia results; these effects were excluded from the baseline-balanced and High-RoB-exclusion sensitivities. No study reported a directly usable lesion-specific mean change with SDchange or an ANCOVA/baseline-adjusted estimate, so no change-score variance or within-participant correlation was imputed (Supplementary Data S7). For readability, the three principal lesion-specific outcomes are presented separately below and summarised in Figure 3; full sensitivity results are provided in Supplementary Table SR4.

**FIGURE 3:**
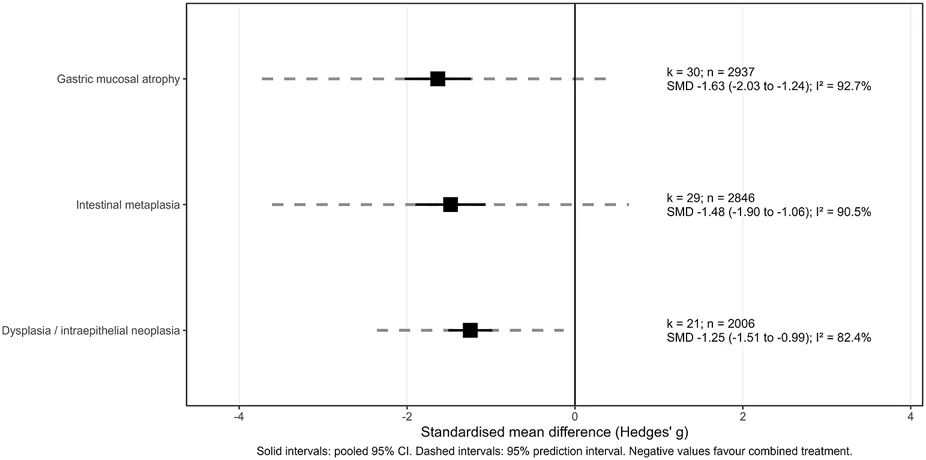
Summary of the exploratory Broad analyses for the three principal pathological outcomes. Pooled SMDs were −1.63 (95% CI −2.03 to −1.24) for atrophy, −1.48 (95% CI −1.90 to −1.06) for intestinal metaplasia, and −1.25 (95% CI −1.51 to −0.99) for dysplasia/IEN. Negative SMDs favour combined treatment. Solid horizontal intervals denote 95% confidence intervals; dashed horizontal intervals denote 95% prediction intervals. Prediction intervals crossed the line of no effect for atrophy (−3.73 to 0.46) and intestinal metaplasia (−3.61 to 0.64), whereas the dysplasia/IEN interval remained below the null (−2.36 to −0.13).

#### 3.4.1 Gastric mucosal atrophy

Thirty studies involving 2,937 participants contributed to the Broad end-of-treatment atrophy analysis. Combined treatment was associated with a lower atrophy score (SMD −1.63, 95% CI −2.03 to −1.24; I^2^ = 92.7%; tau-squared = 1.009), but the 95% prediction interval crossed the null (−3.73 to 0.46). The Restricted clinically comparable histopathology analysis included only four studies and was imprecise (SMD −0.82, 95% CI −1.79 to 0.15).

The baseline-balanced sensitivity remained in the same direction (23 studies; SMD −1.65, 95% CI −2.12 to −1.18), as did exclusion of the High-RoB result (29 studies; SMD −1.67, 95% CI −2.07 to −1.27).

#### 3.4.2 Intestinal metaplasia

Twenty-nine studies involving 2,846 participants contributed to the Broad intestinal-metaplasia analysis. The pooled estimate favoured combined treatment (SMD −1.48, 95% CI −1.90 to −1.06; I^2^ = 90.5%; tau-squared = 1.035), with a prediction interval of −3.61 to 0.64. The Restricted set comprised three studies and was imprecise (SMD −0.40, 95% CI −1.13 to 0.34).

The baseline-balanced sensitivity (22 studies; SMD −1.37, 95% CI −1.83 to −0.92) and High-RoB exclusion sensitivity (28 studies; SMD -1.48, 95% CI −1.92 to −1.05) preserved the average direction.

#### 3.4.3 Dysplasia or intraepithelial neoplasia

Twenty-one studies involving 2,006 participants contributed to the Broad dysplasia/IEN analysis. Combined treatment was associated with a lower score (SMD −1.25, 95% CI −1.51 to −0.99; I^2^ = 82.4%; tau-squared = 0.267). In contrast to atrophy and intestinal metaplasia, the 95% prediction interval remained below the null (−2.36 to −0.13). The Restricted sensitivity was nevertheless based on only three studies and was imprecise (SMD −0.82, 95% CI −2.55 to 0.92).

The baseline-balanced sensitivity included 16 studies and remained in the same average direction (SMD −1.13, 95% CI −1.43 to −0.83; 95% prediction interval −2.25 to −0.01).

Thus, the Broad analyses showed sizeable average differences in all three lesion-specific scores, but the clinically comparable Restricted analyses were based on very few studies and did not exclude the null. These findings argue against interpreting the Broad SMD magnitudes as direct evidence of uniform histological reversal.

### 3.5 Clinically interpretable and composite pathological outcomes

#### 3.5.1 Pure lesion-specific histological improvement

Pure lesion-specific histological improvement was kept separate from continuous scores and from composite response definitions. Atrophy improvement was reported by two studies (158 participants; RR 1.32, 95% CI 0.30–5.87) and intestinal-metaplasia improvement by three studies (291 participants; RR 1.68, 95% CI 0.74–3.84); both estimates were imprecise. Dysplasia/IEN improvement was reported by only one study and was summarised descriptively. Lesion normalisation/resolution, endoscopy-plus-pathology responses, multi-lesion composites, and denominator-conflicted outcomes were not mixed into these pools.

#### 3.5.2 Overall/composite pathology and long-term staging evidence

Eight studies reported a sufficiently comparable end-of-treatment overall/composite pathological response (RR 1.41, 95% CI 1.25 to 1.58; I^2^ approximately 0%), whereas six studies reporting continuous overall/composite scores were highly heterogeneous and imprecise (SMD −2.99, 95% CI −7.64 to 1.66; I^2^ = 97.6%). These constructs were analysed separately from lesion-specific outcomes. A four-arm multicentre trial of Moluodan (88; study ID H20 in the supplementary audit) contained one comparison that met the add-on PICOS: Moluodan plus folic acid versus folic acid alone. Using the 12-month FAS data, global staging improvement was 26/81 versus 59/156 (RR 0.85, 95% CI 0.58–1.24), global histological scoring improvement was 42/81 versus 85/156 (RR 0.95, 95% CI 0.74–1.23), and low-grade dysplasia disappearance was 11/18 versus 21/39 (RR 1.13, 95% CI 0.71–1.81). All three estimates were imprecise and did not exclude no difference. No pooled OLGA/OLGIM meta-analysis was undertaken because only one study provided this type of staging evidence.

### 3.6 *Helicobacter pylori* clearance/negative conversion

Fifteen studies involving 1,511 baseline-positive participants contributed to the Broad *H. pylori* clearance/negative-conversion analysis. Combined treatment was associated with a higher probability of a negative follow-up test (RR 1.25, 95% CI 1.15 to 1.37; I^2^ = 48.0%; 95% prediction interval 1.01–1.56). Restriction to studies with a defined anti-H. pylori regimen yielded a similar estimate (12 studies; RR 1.22, 95% CI 1.13–1.32).

Only three studies satisfied the stricter test-of-cure definition; this estimate was imprecise (RR 1.19, 95% CI 0.91–1.56). The Broad outcome is therefore reported as clearance/negative conversion among baseline-positive participants rather than as confirmed eradication. Post-treatment negative status without a baseline-positive denominator and other *H. pylori* constructs were summarised separately.

### 3.7 Adverse events

The Broad safety evidence set contained 14 independent studies (1,407 participants), including two double-zero studies that did not contribute to the conventional RR calculation. There was no clear evidence of a difference in reported adverse events (RR-contributing studies = 12; RR 0.93, 95% CI 0.58 to 1.50; I^2^ = 34.6%; 95% prediction interval 0.31–2.85). Excluding the S49 composite that incorporated laboratory/organ-function abnormalities produced a similar clinical-adverse-event-only result (13-study evidence set; RR-contributing studies = 11; RR 1.10, 95% CI 0.73–1.68). RD sensitivities retaining the double-zero trials were likewise close to no difference. Qualitative safety statements were not imputed as zero events.

### 3.8 Subgroup, meta-regression, and robustness analyses

Twenty of the 21 specified subgroup interaction tests met the minimum-size rule; dysplasia/IEN by *H. pylori* status was not formally tested because one category contained fewer than three independent studies. No interaction remained statistically significant after Benjamini-Hochberg correction. The closest nominal signal was for intestinal metaplasia by broad conventional-therapy comparator type (raw P = 0.057; adjusted P = 0.893). No robust study-level effect modifier was identified.

Eight of nine specified univariable meta-regression models were estimable; the dysplasia/IEN H. pylori-status model was not performed because of insufficient classifiable studies/variation. None was statistically significant after multiplicity correction. In particular, treatment duration was not associated with effect size when restricted to explicit source-reported durations: coefficient per 4 weeks −0.066 (95% CI −0.520 to 0.387) for atrophy, 0.084 (−0.378–0.546) for intestinal metaplasia, and −0.010 (−0.294 to 0.274) for dysplasia/IEN.

Leave-one-out analyses did not reverse the direction of any primary pathological result: pooled SMDs ranged from −1.69 to −1.53 for atrophy, −1.53 to −1.35 for intestinal metaplasia, and −1.30 to −1.19 for dysplasia/IEN, with all corresponding 95% CIs remaining below zero. Under the extreme-effect rule (absolute SMD >4), exclusion of S10 attenuated atrophy to SMD −1.53 (95% CI -1.87 to −1.19), and exclusion of S1 and S38 attenuated intestinal metaplasia to −1.24 (95% CI −1.47 to −1.01). No dysplasia/IEN study met the extreme-effect criterion.

### 3.9 Reporting bias and small-study effects

The SMD-specific Pustejovsky-Rodgers tests did not detect funnel-plot asymmetry for atrophy (P = 0.443), intestinal metaplasia (P = 0.405), or dysplasia/IEN (P = 0.837). The Harbord test for *H. pylori* clearance/negative conversion was suggestive at the defined P < 0.10 threshold (P = 0.052), whereas no statistical asymmetry signal was detected for reported adverse events (P = 0.385). These tests were interpreted as assessments of small-study effects rather than direct tests of publication bias; substantial heterogeneity and incomplete trial registration were also considered when judging dissemination bias. Full principal-outcome study-level forest plots and contour-enhanced funnel plots are provided in Supplementary Data S5; the principal analysis code, study-level inputs, and validation targets are provided in Supplementary Data S6.

### 3.10 Certainty of evidence

GRADE certainty was low for all three principal pathological score outcomes. Atrophy and intestinal metaplasia were downgraded for serious risk of bias and serious inconsistency. Dysplasia/IEN was likewise downgraded for serious risk of bias and serious inconsistency despite its prediction interval remaining below the null. The SMD-specific Pustejovsky-Rodgers tests did not detect asymmetry for any of the three pathological outcomes, so no dissemination-bias downgrade was applied on that statistical basis. *Helicobacter pylori* clearance/negative conversion was rated low certainty after downgrading for risk of bias and suspected dissemination bias. Reported adverse events were rated low certainty because of risk of bias and imprecision. Table 2 presents the Summary of Findings.

**TABLE 2:** GRADE Summary of Findings for the five principal outcomes. Population: chronic atrophic gastritis or gastric precancerous lesions; intervention: Chinese herbal medicine plus the same conventional therapy; comparator: conventional therapy alone; primary time point: end of treatment.

Outcome	Studies/participants	Effect (95% CI)	Heterogeneity/95% PI	GRADE	Key rating reasons	Approximate NNT/NNH
Gastric mucosal atrophy score	30/2,937	SMD -1.63 (−2.03 to −1.24)	I^2^ = 92.7%; PI -3.73 to 0.46	Low	Risk of bias; serious inconsistency	NA
Intestinal metaplasia score	29/2,846	SMD -1.48 (−1.90 to −1.06)	I^2^ = 90.5%; PI -3.61 to 0.64	Low	Risk of bias; serious inconsistency	NA
Dysplasia/IEN score	21/2,006	SMD -1.25 (−1.51 to −0.99)	I^2^ = 82.4%; PI -2.36 to −0.13	Low	Risk of bias; serious inconsistency	NA
*H. pylori* clearance/negative conversion	15/1,511	RR 1.25 (1.15–1.37) Absolute translation: 176 more/1,000*	I^2^ = 48.0%; PI 1.01 to 1.56	Low	Risk of bias; suspected dissemination bias; strict test-of-cure sensitivity imprecise	NNTB ≈6 (95% CI ≈ 4–10)‡
Reported adverse events	14/1,407	RR 0.93 (0.58–1.50) Absolute translation: 7 fewer/1,000*	I^2^ = 34.6%; PI 0.31 to 2.85	Low	Risk of bias; serious imprecision; no detected Harbord asymmetry signal	No single interpretable NNT/NNH§

*Absolute effects for the two binary principal outcomes were calculated using the pooled observed control‐group risk as the assumed baseline risk. These are study‐set absolute translations, not individualized baseline‐risk estimates. Interpretation: Negative SMDs for pathological scores favour combined treatment. RR > 1 favours clearance/negative conversion; for adverse events, RR < 1 favours fewer reported events.

For *Helicobacter pylori* clearance/negative conversion, the assumed control risk was 692 per 1,000 and the RR corresponds to approximately 869 per 1,000 with combined treatment (95% CI, 796–948), or 176 more per 1,000. For reported adverse events, the assumed control risk was 108 per 1,000 and the RR corresponds to approximately 101 per 1,000 (95% CI, 63–162), or 7 fewer per 1,000 (95% CI, approximately 45 fewer to 54 more). Absolute translations were calculated from the unrounded pooled RRs; RRs displayed in the table are rounded to two decimals. Approximate NNT/NNH values use the same study-set baseline risk.

‡ For *Helicobacter pylori* clearance/negative conversion, NNTB ≈ 6 (95% CI ≈ 4–10) represents one additional negative follow-up test.

§ A single NNT/NNH was not reported for adverse events because the 95% CI for the absolute effect included both fewer and more events.

Original-scale interpretation: contributing lesion scores included ordinal 0–3 or 0/1/2/3 grades, 0/3/6/9 grades, and source-defined or multi-item totals; lower scores indicated less severe pathology after direction harmonisation. Because these ranges are not common clinical units, SMDs cannot be translated directly into lesion disappearance, OLGA/OLGIM down‐staging, dysplasia prevention, or gastric-cancer risk reduction. Pure lesion-specific binary improvement and the single-study eligible H20 add-on stage-transition outcomes are reported separately; study-level scales, ranges and biopsy details are provided in Supplementary Data S7.

GRADE: Randomised-trial evidence started at high certainty and was rated down for risk of bias, inconsistency, indirectness, imprecision, and publication/dissemination bias. Full domain judgements are provided in Supplementary Table SR9.

Abbreviations: CI, confidence interval; IEN, intraepithelial neoplasia; IM, intestinal metaplasia; NNT, number needed to treat; NNTB, number needed to treat for benefit; NNH, number needed to harm; PI, prediction interval; RR, risk ratio; SMD, standardised mean difference.

### 3.11 Other secondary outcomes

For study-defined response outcomes, the final analyses remained directionally favourable but should be interpreted as non-standard composite or subjective outcomes rather than direct evidence of histological regression. The clinical total effective-rate analysis included 33 studies (3,287 participants; RR 1.23, 95% CI 1.19 to 1.27; I^2^ = 0%), the clinical symptom-response analysis included eight studies (905 participants; RR 1.21, 95% CI 1.13 to 1.30; I^2^ = 0%), and the TCM symptom/syndrome effective-rate analysis included 11 studies (1,024 participants; RR 1.22, 95% CI 1.15 to 1.30; I^2^ = 0%). Harbord testing did not show a small-study-effect signal for the TCM response outcome (P = 0.102). Pure endoscopic response was available from five studies (693 participants; RR 1.36, 95% CI 1.10 to 1.68; I^2^ = 50.6%), whereas the endoscopy-plus-pathology joint-response construct was reported by three studies (302 participants; RR 1.53, 95% CI 1.07 to 2.19; I^2^ = 0%). These response constructs were kept separate from the lesion-specific pathological score analyses.

For total TCM symptom/syndrome scores, 11 studies involving 978 participants were pooled. Scores were lower with combined treatment on average (SMD −1.54, 95% CI −2.34 to −0.73), but heterogeneity was substantial (I^2^ = 93.7%; tau^2^ = 1.34) and the 95% prediction interval crossed the null (−4.28 to 1.21). A restricted sensitivity (S5, S6, S17, S49b, H19, and U04; six studies; 571 participants) showed the same average direction (SMD −1.30, 95% CI −1.99 to −0.60) but remained heterogeneous (I^2^ = 87.0%; 95% prediction interval −3.17 to 0.57).

Gastric-function biomarkers also showed heterogeneous results. PGI was higher on average in 16 studies (1,634 participants; SMD 2.50, 95% CI 1.60 to 3.41; I^2^ = 94.7%), with a wide prediction interval crossing the null. In contrast, PGII showed no clear between-group difference in 13 studies (1,312 participants; SMD 0.42, 95% CI -0.30 to 1.15; I^2^ = 95.6%). Seven studies reported PGR (784 participants; MD 2.01, 95% CI 1.15 to 2.86; I^2^ = 90.3%); leave-one-out analyses retained confidence intervals above zero, although substantial heterogeneity persisted. These biomarkers were interpreted as surrogate outcomes rather than direct patient-important effects.

Inflammatory biomarker evidence was sparse and highly heterogeneous. The pooled estimates were CRP, MD −1.55 (three studies; 358 participants; 95% CI −13.92 to 10.82; I^2^ = 99.0%); IL-1beta, MD -8.84 (two studies; 190 participants; 95% CI −69.01 to 51.33); IL-6, MD −7.56 (four studies; 396 participants; 95% CI -13.84 to −1.28; I^2^ = 96.3%); IL-8, MD −8.01 (three studies; 284 participants; 95% CI −24.85 to 8.83; I^2^ = 99.1%); and TNF-alpha, SMD −1.59 (five studies; 524 participants; 95% CI −2.96 to −0.22; I^2^ = 94.6%). The H11 CRP source contained a conflict between the printed arm-level table and narrative direction; the source-verification rule retained the printed arm-level table without reversing values.

Nine individual-symptom outcomes were analysed as a multiplicity-controlled family. Six of the nine outcome-level tests remained below the Benjamini-Hochberg-adjusted 0.05 threshold: poor appetite (13 studies; SMD −1.20, 95% CI −1.55 to −0.84), fatigue/lassitude (eight studies; SMD −1.09, 95% CI −1.55 to −0.64), epigastric/gastric pain (14 studies; SMD −2.13, 95% CI −2.92 to −1.34), fullness/distension (15 studies; SMD −1.47, 95% CI −2.05 to −0.89), loose stool (five studies; SMD −1.98, 95% CI −3.58 to −0.37), and dry mouth/bitter taste (four studies; SMD −2.33, 95% CI −3.60 to −1.06). The displayed 95% confidence intervals are the model-based intervals for each outcome and were not multiplicity-adjusted. Acid reflux/regurgitation, belching, and unsatisfactory stool did not meet the multiplicity-adjusted threshold.

GRADE assessment of the 23 non-principal secondary outcomes yielded low-certainty evidence for nine outcomes and very-low-certainty evidence for the remaining 14; no non-principal secondary outcome was rated moderate or high certainty. For non-principal secondary SMD syntheses, corrected SMD-specific asymmetry testing was not available because the required study-level asymmetry inputs were not preserved; publication/dissemination bias was therefore treated as not assessable rather than as absent. *Helicobacter pylori* clearance/negative conversion and reported adverse events remained principal outcomes and retained the principal-outcome GRADE judgements reported in Table 2. Full secondary estimates, sensitivities, multiplicity adjustment, and domain-level GRADE judgements are provided in the supplementary evidence tables.

## 4 Discussion

### 4.1 Principal findings

This review synthesises 69 randomised studies after full-text eligibility re-assessment and an updated search. The exploratory Broad lesion-specific analyses were associated with lower end-of-treatment scores for atrophy, intestinal metaplasia, and dysplasia/IEN, and the average direction was stable in baseline-balanced, High-RoB-exclusion, leave-one-out, and extreme-effect sensitivity analyses. However, the clinically comparable Restricted histopathology analyses contained only three to four studies and were substantially less precise, with all confidence intervals crossing the null. The evidence therefore supports a possible average short-term difference in pathological scores but does not establish the magnitude of a clinically reproducible histological effect.

The distinction between Broad and Restricted pathology is central to interpretation. SMDs permit combination of different numerical scales only when they measure the same underlying lesion; they do not correct differences in biopsy mapping, site selection, aggregation rules, pathology systems, or denominator definitions. The Broad analyses provide an exploratory summary across heterogeneous measurement systems, whereas the Restricted analyses show what remains under tighter clinical-comparability requirements. Their imprecision is informative rather than contradictory: the most comparable evidence base is currently small.

Heterogeneity remained substantial for all three primary outcomes. Prediction intervals crossed the null for atrophy and intestinal metaplasia, whereas the dysplasia/IEN prediction interval remained below zero. This does not eliminate serious inconsistency because effect magnitudes remained heterogeneous and no robust study-level modifier emerged from subgroup or meta-regression analyses. All three pathology outcomes therefore remain low-certainty evidence.

The *H. pylori* analysis also requires cautious interpretation. The Broad baseline-positive analysis and the anti-H. pylori-regimen subset favoured combined treatment, but the strict test-of-cure sensitivity was based on only three studies and did not exclude no difference. We therefore use the term clearance/negative conversion rather than confirmed eradication. This distinction avoids implying compliance with contemporary test-of-cure timing and medication-withdrawal standards when those details were not consistently reported.

No clear difference in reported adverse events was detected. The conclusion was similar in the clinical-adverse-event-only sensitivity and RD analyses retaining double-zero studies. Nevertheless, safety definitions and ascertainment were inconsistent, follow-up was generally short, and qualitative statements could not be treated as zero events. These data therefore do not establish safety equivalence for the heterogeneous class of herbal interventions. These short trials also cannot exclude rare hepatotoxicity, nephrotoxicity, contamination or batch variability, herb-drug interactions, or long-term harms.

The non-principal secondary outcomes were directionally favourable for several study-defined clinical-response and symptom measures, but these findings do not materially strengthen the pathological conclusion. Composite clinical response definitions were non-standard, symptom outcomes were generally subjective and unblinded, and many continuous secondary outcomes showed substantial or extreme heterogeneity. PGI was higher on average but had very high heterogeneity; PGII showed no clear between-group difference. Inflammatory biomarker results were sparse and inconsistent. After multiplicity adjustment within the nine individual-symptom family, six outcomes retained BH-adjusted P < 0.05, whereas acid reflux/regurgitation, belching, and unsatisfactory stool did not. GRADE certainty for the 23 non-principal secondary outcomes was low for nine and very low for 14, with no moderate- or high-certainty secondary evidence. The statistical homogeneity of the pooled clinical total effective rate should not be interpreted as evidence that the underlying response constructs were clinically equivalent, because definitions and thresholds were study-defined and not fully standardised across trials.

### 4.2 Comparison with previous evidence and clinical interpretation

The direction of the Broad findings is broadly consistent with earlier systematic reviews suggesting potential benefits of traditional Chinese medicine for chronic atrophic gastritis, while those reviews also emphasised low trial quality and heterogeneous interventions (10, 11). The present review differs by maintaining lesion-specific pathology, explicitly separating non-interchangeable pathological constructs, auditing denominators and biopsy methods, applying result-level RoB 2, and presenting both the exploratory Broad estimates and the smaller clinically comparable Restricted evidence base.

Several biological pathways may provide mechanistic plausibility for the short-term differences observed in pathological scores. Experimental and translational evidence has proposed preservation of gastric mucosal barrier function, modulation of inflammatory and immune signalling, improvement of microcirculation, regulation of oxidative stress, and effects on epithelial proliferation and apoptosis (7, 8). However, these mechanisms were not evaluated as causal mediators in the included clinical trials, and mechanistic plausibility should not be interpreted as evidence of durable histological reversal or gastric-cancer prevention.

The findings should be interpreted within established management strategies for gastric precancerous conditions. Current guidance emphasises high-quality endoscopic assessment, systematic biopsy sampling, risk stratification, *H. pylori* eradication when indicated, and surveillance according to lesion extent and risk (4). The Maastricht VI consensus likewise supports *H. pylori* eradication as a cornerstone of gastric-cancer risk reduction (5). Adjunctive herbal therapy should not replace these established measures.

The pooled SMDs are standardised differences in score, not probabilities of lesion disappearance. An SMD cannot determine how many patients moved to a lower OLGA/OLGIM stage, achieved complete resolution of intestinal metaplasia, avoided dysplasia progression, or reduced future gastric-cancer risk. The sparse binary histological improvement analyses and the standalone eligible H20 add-on stage-transition evidence illustrate why future trials should report patient-level histological improvement, persistence, progression, and validated stage changes alongside continuous scores.

### 4.3 Strengths and limitations

The main strengths of this review are the full-text eligibility audit, explicit separation of lesion-specific and composite pathology, source-level denominator and *H. pylori* auditing, result-level RoB 2, a documented outcome-selection hierarchy, modern random-effects inference with prediction intervals, and multiple sensitivity analyses targeting different sources of uncertainty. The search was updated through 16 August 2026, and detailed study-level audit trails are provided in the Supplementary Materials.

Several limitations remain. First, many trials were small and incompletely reported, with allocation concealment, adherence/deviations, blinded pathology assessment, trial registration, protocols, and statistical analysis plans frequently unavailable. One primary-pathology study was judged High risk of bias, while most others raised some concerns. Second, herbal formulas, conventional therapies, treatment durations, patient phenotypes, biopsy protocols, pathology scales, and aggregation methods varied substantially. The intervention table shows that manufacturer, batch, quality-control, and complete composition information were often not reported, limiting reproducibility and preventing a formula-specific class-effect inference.

Third, most principal pathological analyses used end-of-treatment scores because no study provided a directly usable lesion-specific change score with its corresponding variance or an ANCOVA/baseline-adjusted treatment effect. Although randomisation is expected to reduce systematic baseline differences on average, many contributing trials were small and reporting of allocation concealment was incomplete; baseline imbalances may therefore remain relevant in individual trials. The baseline-balanced sensitivity required directly auditable pretreatment values for the same lesion construct and scale and preserved the average direction of the findings, but it cannot substitute for within-trial change-score or baseline-adjusted analyses. Residual influence of baseline pathological severity on the observed post-treatment differences therefore cannot be excluded. Fourth, the Restricted histopathology sets were very small, so estimates under the most stringent comparability criteria were imprecise. Fifth, treatment and follow-up were generally too short to determine durable lesion regression, progression, gastric-cancer incidence, or long-term safety.

Sixth, all included trials were conducted in China, and the evidence base was predominantly composed of Chinese-language reports. Although no language restriction was applied during study identification, this geographic and linguistic concentration limits external generalisability and means that region- or language-related dissemination bias cannot be fully excluded. Arm-specific demographic reporting also remained incomplete after source-level verification: mean-age data were unavailable in 7 of 69 studies and arm-specific sex distribution in 5 of 69 studies. In several trials, demographic characteristics referred to completer or full-analysis-set populations rather than the complete randomised sample. These reporting limitations constrained assessment of demographic comparability and precluded reliable exploration of age- or sex-related effect modification.

Seventh, the SMD-specific asymmetry tests did not detect a statistical signal for any of the three primary pathology outcomes, whereas the Harbord test was suggestive for *H. pylori*. Absence of funnel-plot asymmetry does not exclude selective dissemination, particularly in a body of small, incompletely registered and clinically heterogeneous trials. Corrected SMD-specific asymmetry testing could not be repeated for several non-principal secondary outcomes because the required study-level asymmetry inputs were not preserved. Finally, the historical screening snapshot recorded 26 reports as not retrieved. Their identities were reconstructed during full-text eligibility re-assessment; 15 were obtained through Wanfang Library document delivery and assessed, whereas the remaining 11 could not be obtained despite subsequent searches of the relevant journal official websites and historical archives. The final integrated PRISMA flow therefore reports 11 reports as not retrieved, while SR13 preserves the historical 26-report snapshot and its reconstruction. Because 11 reports remain unavailable, residual retrieval bias cannot be completely excluded.

### 4.4 Implications for clinical practice and future research

The evidence does not support replacing established management with Chinese herbal medicine. If adjunctive use is considered, it should occur alongside indicated *H. pylori* eradication, high-quality endoscopic and pathological assessment, risk stratification, and surveillance. The large Broad SMDs should not be used to promise complete histological reversal or cancer prevention to individual patients.

Future trials should be prospectively registered, adequately powered, multicentre where feasible, and supported by accessible protocols and analysis plans. They should report concealed allocation, blinded central or independent pathological assessment, predefined missing-data handling, and intention-to-treat analyses. Herbal interventions require complete composition, dose, botanical identification, preparation, manufacturer/batch, quality-control procedures, and explicit rules for syndrome-based modification.

Outcome assessment should use reproducible biopsy mapping and internationally interpretable pathology criteria. Trials should report lesion-specific baseline and follow-up distributions, patient-level histological improvement or resolution, OLGA/OLGIM or dysplasia stage transitions where appropriate, and longer-term persistence/progression outcomes. *Helicobacter pylori* outcomes should use accepted test-of-cure procedures, and adverse events should be systematically collected rather than described qualitatively.

### 4.5 Conclusion

Chinese herbal medicine added to the same conventional therapy may be associated with lower short-term scores for gastric mucosal atrophy, intestinal metaplasia, and dysplasia/IEN on average. However, these Broad effects were highly heterogeneous, and the more clinically comparable Restricted analyses were based on few studies and were imprecise. The pathology evidence remains low certainty.

Combined treatment was associated with higher *H. pylori* clearance/negative conversion in the Broad baseline-positive analysis, but the strict test-of-cure evidence was sparse and imprecise. There was no clear evidence of a difference in reported adverse events, which should not be interpreted as proof of equivalent safety.

The findings support possible short-term differences in pathological scores rather than definitive histological reversal or prevention of progression to gastric cancer. The evidence is insufficient to recommend a class effect for Chinese herbal medicine as a whole; efficacy and safety should be established for specific formulas or products in adequately designed confirmatory trials. Chinese herbal medicine should not substitute for established *H. pylori* management, high-quality endoscopic and pathological assessment, risk stratification, or surveillance.

## Data Availability

The original contributions presented in the study are included in the article/Supplementary Material, further inquiries can be directed to the corresponding authors.
