# Supplementary material for: Chinese herbal medicine combined with conventional therapy for gastric precancerous lesions: a systematic review and meta-analysis
Source: Oncol Rev. 2026 Oct 19;20:1947905. doi: 10.3389/or.2026.1947905 (PMC13724460; doi:10.3389/or.2026.1947905)
Supplement: Supplementary file 1 [file DataSheet5.docx]

**Supplementary Figures: Principal Outcome Forest and Funnel Plots**

These figures display the final principal-outcome study-level inputs and method-corrected small-study-effect assessments. The forest plots, study-level effect geometry, pooled estimates, and prediction intervals are unchanged. For the three SMD pathology outcomes, conventional Egger testing was replaced by the SMD-specific Pustejovsky-Rodgers test; this changed the asymmetry-test interpretation and downstream GRADE judgement but not the plotted study effects or pooled estimates. Study labels use the stable study ID and publication year; full bibliographic identities are provided in the study-characteristics tables and Reference Master.

**Supplementary Figure S1. Study-level forest plot for gastric mucosal atrophy.**

Study effects are Hedges' g standardised mean differences from the frozen Final69 Phase 4–5 study-effect table. Negative values favour Chinese herbal medicine plus conventional therapy. Squares are proportional to archived random-effects weights; horizontal lines are study-level 95% confidence intervals. The diamond is the frozen random-effects pooled estimate and the dashed line on the pooled row is the frozen 95% prediction interval.

**Supplementary Figure S2. Study-level forest plot for intestinal metaplasia.**

Display conventions are the same as Supplementary Figure S1. Negative values favour combined treatment.

**Supplementary Figure S3. Study-level forest plot for dysplasia or intraepithelial neoplasia.**

Display conventions are the same as Supplementary Figure S1. Negative values favour combined treatment.

**Supplementary Figure S4. Study-level forest plot for H. pylori clearance/negative conversion.**

Study-level risk ratios are displayed from the frozen Final69 binary input table. RR >1 favours combined treatment. The diamond and dashed pooled-row interval show the frozen random-effects estimate and 95% prediction interval.

**Supplementary Figure S5. Study-level forest plot for reported adverse events.**

The broad safety evidence set contains 14 studies. Twelve have estimable conventional risk ratios; two double-zero studies are retained in the evidence set but do not contribute an estimable conventional RR. RR <1 favours fewer reported adverse events with combined treatment.

**Supplementary Figure S6. Contour-enhanced funnel plot for gastric mucosal atrophy.**

The plot uses the final study-level Hedges' g estimates and standard errors. Shaded contours correspond to two-sided significance regions around the null. The SMD-specific Pustejovsky-Rodgers test showed no statistical asymmetry signal (P=0.443). Absence of a signal does not prove absence of dissemination bias.

**Supplementary Figure S7. Contour-enhanced funnel plot for intestinal metaplasia.**

The SMD-specific Pustejovsky-Rodgers test showed no statistical asymmetry signal (P=0.405). Interpretation remains cautious because substantial heterogeneity limits the diagnostic value of funnel asymmetry.

**Supplementary Figure S8. Contour-enhanced funnel plot for dysplasia or intraepithelial neoplasia.**

The SMD-specific Pustejovsky-Rodgers test showed no statistical asymmetry signal (P=0.837).

**Supplementary Figure S9. Contour-enhanced funnel plot for H. pylori clearance/negative conversion.**

The funnel is displayed using log-risk-ratio geometry with risk-ratio tick labels. The Harbord modified score test was suggestive at the P<0.10 threshold (P=0.052), but this does not establish dissemination bias.

**Supplementary Figure S10. Contour-enhanced funnel plot for reported adverse events.**

The broad evidence set contains 14 studies; 12 studies have estimable RRs for the conventional RR-based asymmetry assessment. The Harbord modified score test did not show a statistical asymmetry signal (P=0.385).
