# Supplementary material for: Chinese herbal medicine combined with conventional therapy for gastric precancerous lesions: a systematic review and meta-analysis
Source: Oncol Rev. 2026 Oct 19;20:1947905. doi: 10.3389/or.2026.1947905 (PMC13724460; doi:10.3389/or.2026.1947905)
Supplement: Supplementary file 3 [file Supplementaryfile1.docx]

Supplementary Appendix 1

Complete original and revision-stage update search strategies

# 1 Part A. Original search strategies

## 1.1 Search period: from database inception to 31 March 2026; final original search date: 31 March 2026.

Controlled vocabulary and free-text terms were adapted to the indexing system and syntax of each database or registry. The strategies below reproduce the final original searches used in the submitted review.

Controlled vocabulary and free-text terms were adapted to the indexing system and syntax of each database or registry. The strategies below present the final searches used in the review.

**1. PubMed**

("Gastritis, Atrophic"[Mesh]OR "Intestinal Metaplasia"[Mesh]OR atrophic gastritis[Title/Abstract]OR chronic atrophic gastritis[Title/Abstract]OR intestinal metaplasia[Title/Abstract]OR gastric intestinal metaplasia[Title/Abstract]OR gastric dysplasia[Title/Abstract]OR gastric epithelial dysplasia[Title/Abstract]OR gastric intraepithelial neoplasia[Title/Abstract]OR low-grade intraepithelial neoplasia[Title/Abstract]OR low grade intraepithelial neoplasia[Title/Abstract]OR low-grade dysplasia[Title/Abstract]OR low grade dysplasia[Title/Abstract]OR gastric precancerous lesion*[Title/Abstract]OR gastric premalignant lesion*[Title/Abstract]OR gastric precancerous condition*[Title/Abstract]OR gastric premalignant condition*[Title/Abstract])

AND("Drugs, Chinese Herbal"[Mesh]OR "Plant Preparations"[Mesh]OR Chinese herbal medicine[Title/Abstract]OR Chinese herbal drug*[Title/Abstract]OR herbal medicine[Title/Abstract]OR herbal formula[Title/Abstract]OR herbal decoction[Title/Abstract]OR decoction[Title/Abstract]OR Chinese patent medicine[Title/Abstract]OR patent medicine[Title/Abstract])

AND

(randomized controlled trial[Publication Type]OR controlled clinical trial[Publication Type]OR random*[Title/Abstract]OR randomly[Title/Abstract]OR trial[Title/Abstract])

NOT

(review[Publication Type]OR meta-analysis[Publication Type]OR systematic review[Title/Abstract]OR meta-analysis[Title/Abstract] OR review[Title])

NOT

(non-atrophic gastritis[Title/Abstract]OR non atrophic gastritis[Title/Abstract]OR superficial gastritis[Title/Abstract]OR chronic non-atrophic gastritis[Title/Abstract]OR chronic non atrophic gastritis[Title/Abstract])

NOT

(animals[Mesh] NOT humans[Mesh])

NOT

(rat[Title/Abstract]OR rats[Title/Abstract]OR mouse[Title/Abstract]OR mice[Title/Abstract]OR rabbit[Title/Abstract]OR rabbits[Title/Abstract]OR cell[Title/Abstract]OR cells[Title/Abstract])

NOT

(acupuncture[Title/Abstract]OR electroacupuncture[Title/Abstract]OR electro-acupuncture[Title/Abstract]OR moxibustion[Title/Abstract]OR acupoint[Title/Abstract]OR acupressure[Title/Abstract]OR auricular[Title/Abstract]OR tui na[Title/Abstract]OR tuina[Title/Abstract]OR massage[Title/Abstract]OR manual therapy[Title/Abstract]OR cupping[Title/Abstract])

**2. Embase**

(

'atrophic gastritis'/exp

OR 'intestinal metaplasia'/exp

OR 'gastric dysplasia'/exp

OR 'atrophic gastritis':ti,ab

OR 'chronic atrophic gastritis':ti,ab

OR 'intestinal metaplasia':ti,ab

OR 'gastric intestinal metaplasia':ti,ab

OR 'gastric dysplasia':ti,ab

OR 'gastric epithelial dysplasia':ti,ab

OR 'gastric intraepithelial neoplasia':ti,ab

OR 'low-grade intraepithelial neoplasia':ti,ab

OR 'low grade intraepithelial neoplasia':ti,ab

OR 'low-grade dysplasia':ti,ab

OR 'low grade dysplasia':ti,ab

OR 'gastric precancerous lesion*':ti,ab

OR 'gastric premalignant lesion*':ti,ab

OR 'gastric precancerous condition*':ti,ab

OR 'gastric premalignant condition*':ti,ab

)

AND

(

'chinese herbal drug'/exp

OR 'herbal medicine'/exp

OR 'chinese herbal medicine':ti,ab

OR 'chinese herbal drug*':ti,ab

OR 'herbal medicine':ti,ab

OR 'herbal formula':ti,ab

OR 'herbal decoction':ti,ab

OR decoction:ti,ab

OR 'chinese patent medicine':ti,ab

OR 'patent medicine':ti,ab

)

AND

(

'randomized controlled trial'/exp

OR random*:ti,ab

OR randomly:ti,ab

OR placebo:ti,ab

)

NOT

(

review:it

OR 'systematic review':ti,ab

OR 'meta analysis':ti,ab

)

NOT

(

'non-atrophic gastritis':ti,ab

OR 'non atrophic gastritis':ti,ab

OR 'superficial gastritis':ti,ab

OR 'chronic non-atrophic gastritis':ti,ab

OR 'chronic non atrophic gastritis':ti,ab

)

NOT

(

acupuncture:ti,ab

OR electroacupuncture:ti,ab

OR 'electro-acupuncture':ti,ab

OR moxibustion:ti,ab

OR acupoint:ti,ab

OR acupressure:ti,ab

OR auricular:ti,ab

OR 'tui na':ti,ab

OR tuina:ti,ab

OR massage:ti,ab

OR 'manual therapy':ti,ab

OR cupping:ti,ab

)

NOT

(

rat:ti,ab

OR rats:ti,ab

OR mouse:ti,ab

OR mice:ti,ab

OR rabbit:ti,ab

OR rabbits:ti,ab

OR cell:ti,ab

OR cells:ti,ab

)

NOT

(

'artificial intelligence':ti,ab

OR 'machine learning':ti,ab

OR 'deep learning':ti,ab

OR 'neural network':ti,ab

OR 'prediction model':ti,ab

OR 'predictive model':ti,ab

OR 'risk model':ti,ab

OR 'diagnostic model':ti,ab

OR nomogram:ti,ab

OR radiomics:ti,ab

OR algorithm*:ti,ab

)

NOT

(

mechanism:ti,ab

OR mechanisms:ti,ab

OR pathway:ti,ab

OR pathways:ti,ab

OR molecular:ti,ab

OR apoptosis:ti,ab

OR proliferation:ti,ab

OR signaling:ti,ab

OR 'signal transduction':ti,ab

OR biomarker*:ti,ab

OR gene*:ti,ab

OR protein*:ti,ab

OR 'in vitro':ti,ab

)

**3. Cochrane Library**

#1 [mh "Gastritis, Atrophic"] OR [mh "Intestinal Metaplasia"]

#2 atrophic gastritis:ti,ab,kw OR chronic atrophic gastritis:ti,ab,kw

#3 intestinal metaplasia:ti,ab,kw OR gastric intestinal metaplasia:ti,ab,kw

#4 gastric dysplasia:ti,ab,kw OR gastric epithelial dysplasia:ti,ab,kw OR gastric intraepithelial neoplasia:ti,ab,kw

#5 low-grade intraepithelial neoplasia:ti,ab,kw OR low grade intraepithelial neoplasia:ti,ab,kw OR low-grade dysplasia:ti,ab,kw OR low grade dysplasia:ti,ab,kw

#6 gastric precancerous lesion*:ti,ab,kw OR gastric premalignant lesion*:ti,ab,kw OR gastric precancerous condition*:ti,ab,kw OR gastric premalignant condition*:ti,ab,kw

#7 #1 OR #2 OR #3 OR #4 OR #5 OR #6

#8 [mh "Drugs, Chinese Herbal"] OR [mh "Plant Preparations"]

#9 chinese herbal medicine:ti,ab,kw OR chinese herbal drug*:ti,ab,kw OR herbal medicine:ti,ab,kw

#10 herbal formula:ti,ab,kw OR herbal decoction:ti,ab,kw OR decoction:ti,ab,kw

#11 chinese patent medicine:ti,ab,kw OR patent medicine:ti,ab,kw

#12 #8 OR #9 OR #10 OR #11

#13 non-atrophic gastritis:ti,ab,kw OR non atrophic gastritis:ti,ab,kw OR superficial gastritis:ti,ab,kw OR chronic non-atrophic gastritis:ti,ab,kw OR chronic non atrophic gastritis:ti,ab,kw

#14 acupuncture:ti,ab,kw OR electroacupuncture:ti,ab,kw OR electro-acupuncture:ti,ab,kw OR moxibustion:ti,ab,kw OR acupoint:ti,ab,kw OR acupressure:ti,ab,kw OR auricular:ti,ab,kw OR tui na:ti,ab,kw OR tuina:ti,ab,kw OR massage:ti,ab,kw OR manual therapy:ti,ab,kw OR cupping:ti,ab,kw

#15 artificial intelligence:ti,ab,kw OR machine learning:ti,ab,kw OR deep learning:ti,ab,kw OR neural network:ti,ab,kw OR prediction model:ti,ab,kw OR predictive model:ti,ab,kw OR risk model:ti,ab,kw OR diagnostic model:ti,ab,kw OR nomogram:ti,ab,kw OR radiomics:ti,ab,kw OR algorithm*:ti,ab,kw

#16 #7 AND #12

#17 #16 NOT #13

#18 #17 NOT #14

#19 #18 NOT #15

**4. Web of Science Core Collection**

TS=(

("atrophic gastritis" OR "chronic atrophic gastritis" OR "intestinal metaplasia" OR "gastric intestinal metaplasia" OR "gastric dysplasia" OR "gastric epithelial dysplasia" OR "gastric intraepithelial neoplasia" OR "low-grade intraepithelial neoplasia" OR "low grade intraepithelial neoplasia" OR "low-grade dysplasia" OR "low grade dysplasia" OR "gastric precancerous lesion*" OR "gastric premalignant lesion*" OR "gastric precancerous condition*" OR "gastric premalignant condition*")

AND

("chinese herbal medicine" OR "chinese herbal drug*" OR "herbal medicine" OR "herbal formula" OR "herbal decoction" OR decoction OR "chinese patent medicine" OR "patent medicine")

AND

(random* OR randomly OR placebo OR trial)

)

NOT TS=("non-atrophic gastritis" OR "non atrophic gastritis" OR "superficial gastritis" OR "chronic non-atrophic gastritis" OR "chronic non atrophic gastritis")

NOT TS=(acupuncture OR electroacupuncture OR "electro-acupuncture" OR moxibustion OR acupoint OR acupressure OR auricular OR "tui na" OR tuina OR massage OR "manual therapy" OR cupping)

NOT TS=("artificial intelligence" OR "machine learning" OR "deep learning" OR "neural network" OR "prediction model" OR "predictive model" OR "risk model" OR "diagnostic model" OR nomogram OR radiomics OR algorithm*)

NOT TS=(rat OR rats OR mouse OR mice OR rabbit OR rabbits OR cell OR cells)

NOT TS=(mechanism OR mechanisms OR pathway OR pathways OR molecular OR apoptosis OR proliferation OR signaling OR "signal transduction" OR biomarker* OR gene* OR protein* OR "in vitro")

**5. China National Knowledge Infrastructure (CNKI)**

((TI %= '胃癌前病变' OR KY %= '胃癌前病变' OR AB %= '胃癌前病变')OR

(TI %= '慢性萎缩性胃炎' OR KY %= '慢性萎缩性胃炎' OR AB %= '慢性萎缩性胃炎')OR

(TI %= '胃黏膜萎缩' OR KY %= '胃黏膜萎缩' OR AB %= '胃黏膜萎缩')OR

(TI %= '肠上皮化生' OR KY %= '肠上皮化生' OR AB %= '肠上皮化生')OR

(TI %= '胃肠上皮化生' OR KY %= '胃肠上皮化生' OR AB %= '胃肠上皮化生')OR

(TI %= '胃异型增生' OR KY %= '胃异型增生' OR AB %= '胃异型增生')OR

(TI %= '胃黏膜异型增生' OR KY %= '胃黏膜异型增生' OR AB %= '胃黏膜异型增生')OR

(TI %= '胃上皮内瘤变' OR KY %= '胃上皮内瘤变' OR AB %= '胃上皮内瘤变')OR

(TI %= '胃低级别上皮内瘤变' OR KY %= '胃低级别上皮内瘤变' OR AB %= '胃低级别上皮内瘤变'))AND

((TI %= '中药' OR KY %= '中药' OR AB %= '中药')OR(TI %= '中药治疗' OR KY %= '中药治疗' OR AB %= '中药治疗')OR(TI %= '中西医结合' OR KY %= '中西医结合' OR AB %= '中西医结合')

OR(TI %= '中药联合西药' OR KY %= '中药联合西药' OR AB %= '中药联合西药')OR(TI %= '中药加用西药' OR KY %= '中药加用西药' OR AB %= '中药加用西药'))

AND

((TI %= '随机' OR KY %= '随机' OR AB %= '随机')OR

(TI %= '随机对照' OR KY %= '随机对照' OR AB %= '随机对照')OR

(TI %= '随机分组' OR KY %= '随机分组' OR AB %= '随机分组')OR

(TI %= '临床试验' OR KY %= '临床试验' OR AB %= '临床试验'))

NOT((TI %= '小鼠' OR KY %= '小鼠' OR AB %= '小鼠')

OR(TI %= '大鼠' OR KY %= '大鼠' OR AB %= '大鼠')OR

(TI %= '动物实验' OR KY %= '动物实验' OR AB %= '动物实验')OR

(TI %= '细胞' OR KY %= '细胞' OR AB %= '细胞')OR

(TI %= '细胞实验' OR KY %= '细胞实验' OR AB %= '细胞实验'))

NOT((TI %= '机制' OR KY %= '机制' OR AB %= '机制')

OR(TI %= '作用机制' OR KY %= '作用机制' OR AB %= '作用机制')

OR(TI %= '分子机制' OR KY %= '分子机制' OR AB %= '分子机制')

OR(TI %= '信号通路' OR KY %= '信号通路' OR AB %= '信号通路'))

NOT((TI %= 'Meta分析' OR KY %= 'Meta分析' OR AB %= 'Meta分析')OR

(TI %= '荟萃分析' OR KY %= '荟萃分析' OR AB %= '荟萃分析')OR

(TI %= '系统评价' OR KY %= '系统评价' OR AB %= '系统评价')OR

(TI %= '系统综述' OR KY %= '系统综述' OR AB %= '系统综述')OR

(TI %= '综述' OR KY %= '综述' OR AB %= '综述'))

NOT(

(TI %= '宫颈' OR KY %= '宫颈' OR AB %= '宫颈')OR

(TI %= '宫颈上皮内瘤变' OR KY %= '宫颈上皮内瘤变' OR AB %= '宫颈上皮内瘤变')OR

(TI %= 'CIN' OR KY %= 'CIN' OR AB %= 'CIN'))

NOT((TI %= '针灸' OR KY %= '针灸' OR AB %= '针灸')OR

(TI %= '电针' OR KY %= '电针' OR AB %= '电针')OR

(TI %= '艾灸' OR KY %= '艾灸' OR AB %= '艾灸')OR

(TI %= '穴位贴敷' OR KY %= '穴位贴敷' OR AB %= '穴位贴敷')OR

(TI %= '穴位注射' OR KY %= '穴位注射' OR AB %= '穴位注射')OR(TI %= '耳穴压豆' OR KY %= '耳穴压豆' OR AB %= '耳穴压豆')OR

(TI %= '耳穴' OR KY %= '耳穴' OR AB %= '耳穴')OR

(TI %= '耳穴贴压' OR KY %= '耳穴贴压' OR AB %= '耳穴贴压')OR

(TI %= '穴位埋线' OR KY %= '穴位埋线' OR AB %= '穴位埋线')OR

(TI %= '穴位埋针' OR KY %= '穴位埋针' OR AB %= '穴位埋针')OR

(TI %= '推拿' OR KY %= '推拿' OR AB %= '推拿')OR

(TI %= '按摩' OR KY %= '按摩' OR AB %= '按摩')OR

(TI %= '拔罐' OR KY %= '拔罐' OR AB %= '拔罐')OR

(TI %= '刮痧' OR KY %= '刮痧' OR AB %= '刮痧')OR

(TI %= '穴位刺激' OR KY %= '穴位刺激' OR AB %= '穴位刺激')OR

(TI %= '梅花针' OR KY %= '梅花针' OR AB %= '梅花针'))

**6. Wanfang Data**

主题:(

(胃癌前病变 OR 慢性萎缩性胃炎 OR 胃黏膜萎缩 OR 肠上皮化生 OR 胃肠上皮化生 OR 胃异型增生 OR 胃黏膜异型增生 OR 胃上皮内瘤变 OR 胃低级别上皮内瘤变)

AND

(中药 OR 中药治疗 OR 中西医结合 OR 中药联合西药 OR 中药加用西药)

AND

(随机 OR 随机对照 OR 随机分组 OR 临床试验)

)

NOT

主题:(小鼠 OR 大鼠 OR 动物实验 OR 细胞 OR 细胞实验)

NOT

主题:(机制 OR 作用机制 OR 分子机制 OR 信号通路)

NOT

主题:(Meta分析 OR 荟萃分析 OR 系统评价 OR 系统综述 OR 综述)

NOT

主题:(宫颈 OR 宫颈上皮内瘤变 OR CIN)

NOT

主题:(针灸 OR 电针 OR 艾灸 OR 穴位贴敷 OR 穴位注射 OR 耳穴压豆 OR 耳穴 OR 耳穴贴压 OR 穴位埋线 OR 穴位埋针 OR 推拿 OR 按摩 OR 拔罐 OR 刮痧 OR 穴位刺激 OR 梅花针)

**7. VIP Chinese Science and Technology Journal Database (VIP)**

(

(T=胃癌前病变 OR K=胃癌前病变 OR R=胃癌前病变)

OR

(T=慢性萎缩性胃炎 OR K=慢性萎缩性胃炎 OR R=慢性萎缩性胃炎)

OR

(T=胃黏膜萎缩 OR K=胃黏膜萎缩 OR R=胃黏膜萎缩)

OR

(T=肠上皮化生 OR K=肠上皮化生 OR R=肠上皮化生)

OR

(T=胃肠上皮化生 OR K=胃肠上皮化生 OR R=胃肠上皮化生)

OR

(T=胃异型增生 OR K=胃异型增生 OR R=胃异型增生)

OR

(T=胃黏膜异型增生 OR K=胃黏膜异型增生 OR R=胃黏膜异型增生)

OR

(T=胃上皮内瘤变 OR K=胃上皮内瘤变 OR R=胃上皮内瘤变)

OR

(T=胃低级别上皮内瘤变 OR K=胃低级别上皮内瘤变 OR R=胃低级别上皮内瘤变)

)

AND

(

(T=中药 OR K=中药 OR R=中药)

OR

(T=中药治疗 OR K=中药治疗 OR R=中药治疗)

OR

(T=中西医结合 OR K=中西医结合 OR R=中西医结合)

OR

(T=中药联合西药 OR K=中药联合西药 OR R=中药联合西药)

OR

(T=中药加用西药 OR K=中药加用西药 OR R=中药加用西药)

)

AND

(

(T=随机 OR K=随机 OR R=随机)

OR

(T=随机对照 OR K=随机对照 OR R=随机对照)

OR

(T=随机分组 OR K=随机分组 OR R=随机分组)

OR

(T=临床试验 OR K=临床试验 OR R=临床试验)

)

NOT

(

(T=小鼠 OR K=小鼠 OR R=小鼠)

OR

(T=大鼠 OR K=大鼠 OR R=大鼠)

OR

(T=动物实验 OR K=动物实验 OR R=动物实验)

OR

(T=细胞 OR K=细胞 OR R=细胞)

OR

(T=细胞实验 OR K=细胞实验 OR R=细胞实验)

)

NOT

(

(T=机制 OR K=机制 OR R=机制)

OR

(T=作用机制 OR K=作用机制 OR R=作用机制)

OR

(T=分子机制 OR K=分子机制 OR R=分子机制)

OR

(T=信号通路 OR K=信号通路 OR R=信号通路)

)

NOT

(

(T=Meta分析 OR K=Meta分析 OR R=Meta分析)

OR

(T=荟萃分析 OR K=荟萃分析 OR R=荟萃分析)

OR

(T=系统评价 OR K=系统评价 OR R=系统评价)

OR

(T=系统综述 OR K=系统综述 OR R=系统综述)

OR

(T=综述 OR K=综述 OR R=综述)

)

NOT

(

(T=宫颈 OR K=宫颈 OR R=宫颈)

OR

(T=宫颈上皮内瘤变 OR K=宫颈上皮内瘤变 OR R=宫颈上皮内瘤变)

OR

(T=CIN OR K=CIN OR R=CIN)

)

NOT

(

(T=针灸 OR K=针灸 OR R=针灸)

OR

(T=电针 OR K=电针 OR R=电针)

OR

(T=艾灸 OR K=艾灸 OR R=艾灸)

OR

(T=穴位贴敷 OR K=穴位贴敷 OR R=穴位贴敷)

OR

(T=穴位注射 OR K=穴位注射 OR R=穴位注射)

OR

(T=耳穴压豆 OR K=耳穴压豆 OR R=耳穴压豆)

OR

(T=耳穴 OR K=耳穴 OR R=耳穴)

OR

(T=耳穴贴压 OR K=耳穴贴压 OR R=耳穴贴压)

OR

(T=穴位埋线 OR K=穴位埋线 OR R=穴位埋线)

OR

(T=穴位埋针 OR K=穴位埋针 OR R=穴位埋针)

OR

(T=推拿 OR K=推拿 OR R=推拿)

OR

(T=按摩 OR K=按摩 OR R=按摩)

OR

(T=拔罐 OR K=拔罐 OR R=拔罐)

OR

(T=刮痧 OR K=刮痧 OR R=刮痧)

OR

(T=穴位刺激 OR K=穴位刺激 OR R=穴位刺激)

OR

(T=梅花针 OR K=梅花针 OR R=梅花针)

)

**8. SinoMed (China Biology Medicine database)**

(

胃癌前病变 OR 慢性萎缩性胃炎 OR 胃黏膜萎缩 OR 肠上皮化生 OR 胃肠上皮化生 OR 胃异型增生 OR 胃黏膜异型增生 OR 胃上皮内瘤变 OR 胃低级别上皮内瘤变

)

AND

(

中药 OR 中药治疗 OR 中西医结合 OR 中药联合西药 OR 中药加用西药

)

AND

(

随机 OR 随机对照 OR 随机分组 OR 临床试验

)

NOT

(

小鼠 OR 大鼠 OR 动物实验 OR 细胞 OR 细胞实验

)

NOT

(

针灸 OR 电针 OR 艾灸 OR 穴位贴敷 OR 穴位注射 OR 耳穴压豆 OR 耳穴 OR 推拿 OR 按摩 OR 拔罐 OR 刮痧

)

NOT

(

Meta分析 OR 荟萃分析 OR 系统评价 OR 系统综述 OR 综述 OR 网络药理 OR 网络药理学 OR 数据挖掘 OR 知识图谱 OR 研究进展

)

**9. Chinese Clinical Trial Registry (ChiCTR)**

Search date: 31 March 2026.

Because the ChiCTR platform does not readily support a single complex Boolean query, the following disease-related terms were searched separately. Retrieved records were then screened using the intervention-related terms and deduplicated against records from the other trial registries.

**Disease-related search terms:**

慢性萎缩性胃炎

胃癌前病变

胃黏膜萎缩

肠上皮化生

胃异型增生

胃上皮内瘤变

低级别异型增生

低级别上皮内瘤变

chronic atrophic gastritis

gastric precancerous lesions

intestinal metaplasia

gastric dysplasia

gastric intraepithelial neoplasia

**Intervention-related screening terms:**

中药 OR 中医药 OR 中药复方 OR 中药汤剂 OR 中成药 OR 中西医结合 OR 中药联合西药 OR Chinese herbal medicine OR herbal medicine OR decoction

**10. ClinicalTrials.gov**

("atrophic gastritis" OR "intestinal metaplasia" OR "gastric dysplasia" OR "gastric intraepithelial neoplasia")

AND

("Chinese herbal medicine" OR herbal OR decoction)

**11. World Health Organization International Clinical Trials Registry Platform (WHO ICTRP)**

Search date: 31 March 2026.

**Title search terms entered separately:**

atrophic gastritis

intestinal metaplasia

gastric dysplasia

gastric precancerous lesions

Part B. Revision-stage update search strategies and audit

**Chinese herbal medicine combined with conventional therapy for gastric precancerous lesions**

Date compiled: 16 August 2026

Important note: This section records the update searches actually executed during revision. The search framework was not redesigned. The original search concepts were retained, with only platform-specific syntax adaptations where required. Bibliographic databases were updated for 2026, and trial registries were rerun without date restrictions.

# 2 Overview of databases/platforms and update methods

| No. | Database/platform | Revision-stage update method | Notes |
| --- | --- | --- | --- |
| 1 | PubMed/MEDLINE | Original strategy + Create Date restriction | 2026-04-01 to 2026-08-16 |
| 2 | Web of Science Core Collection | Original strategy rerun in full, then filtered to 2026 | Exported Full Record and Cited References |
| 3 | Cochrane CENTRAL | Original logic retained; quoted wildcard syntax adapted to NEXT where required | Date added: 2026-04-01 to 2026-08-16 |
| 4 | Embase | Original strategy retained | Verified Emtree term 'atrophic gastritis'/exp |
| 5 | CNKI | Original Chinese search logic retained and filtered to 2026 | Title/keyword/abstract fields |
| 6 | Wanfang Data | Original topic strategy retained and filtered to 2026 | Original NOT exclusions retained |
| 7 | VIP | Original T/K/R field strategy retained and filtered to 2026 | Original NOT exclusions retained |
| 8 | SinoMed | Original strategy retained and filtered to 2026 | Original NOT exclusions retained |
| 9 | ClinicalTrials.gov | Original Expert Search rerun across full registry | No date/status restriction |
| 10 | WHO ICTRP | Four Title terms searched separately across full registry | Deduplicated by TrialID |
| 11 | ChiCTR | Disease terms searched in Public/Scientific title | Status All; deduplicated by registration number |

# 3 Detailed database and registry strategies

## 3.1 1. PubMed/MEDLINE

Update implementation: the original PubMed strategy was retained and restricted using Create Date ([crdt]) for the update window.

(

"Gastritis, Atrophic"[Mesh]

OR atrophic gastritis[Title/Abstract]

OR chronic atrophic gastritis[Title/Abstract]

OR intestinal metaplasia[Title/Abstract]

OR gastric intestinal metaplasia[Title/Abstract]

OR gastric dysplasia[Title/Abstract]

OR gastric epithelial dysplasia[Title/Abstract]

OR gastric intraepithelial neoplasia[Title/Abstract]

OR low-grade intraepithelial neoplasia[Title/Abstract]

OR low grade intraepithelial neoplasia[Title/Abstract]

OR low-grade dysplasia[Title/Abstract]

OR low grade dysplasia[Title/Abstract]

OR gastric precancerous lesion*[Title/Abstract]

OR gastric premalignant lesion*[Title/Abstract]

OR gastric precancerous condition*[Title/Abstract]

OR gastric premalignant condition*[Title/Abstract]

)

AND

(

"Drugs, Chinese Herbal"[Mesh]

OR "Plant Preparations"[Mesh]

OR Chinese herbal medicine[Title/Abstract]

OR Chinese herbal drug*[Title/Abstract]

OR herbal medicine[Title/Abstract]

OR herbal formula[Title/Abstract]

OR herbal decoction[Title/Abstract]

OR decoction[Title/Abstract]

OR Chinese patent medicine[Title/Abstract]

OR patent medicine[Title/Abstract]

)

AND

(

randomized controlled trial[Publication Type]

OR controlled clinical trial[Publication Type]

OR random*[Title/Abstract]

OR randomly[Title/Abstract]

OR trial[Title/Abstract]

)

NOT

(

review[Publication Type]

OR meta-analysis[Publication Type]

OR systematic review[Title/Abstract]

OR meta-analysis[Title/Abstract]

OR review[Title]

)

NOT

(

non-atrophic gastritis[Title/Abstract]

OR non atrophic gastritis[Title/Abstract]

OR superficial gastritis[Title/Abstract]

OR chronic non-atrophic gastritis[Title/Abstract]

OR chronic non atrophic gastritis[Title/Abstract]

)

NOT

(animals[Mesh] NOT humans[Mesh])

NOT

(

rat[Title/Abstract]

OR rats[Title/Abstract]

OR mouse[Title/Abstract]

OR mice[Title/Abstract]

OR rabbit[Title/Abstract]

OR rabbits[Title/Abstract]

OR cell[Title/Abstract]

OR cells[Title/Abstract]

)

NOT

(

acupuncture[Title/Abstract]

OR electroacupuncture[Title/Abstract]

OR electro-acupuncture[Title/Abstract]

OR moxibustion[Title/Abstract]

OR acupoint[Title/Abstract]

OR acupressure[Title/Abstract]

OR auricular[Title/Abstract]

OR tui na[Title/Abstract]

OR tuina[Title/Abstract]

OR massage[Title/Abstract]

OR manual therapy[Title/Abstract]

OR cupping[Title/Abstract]

)

AND

("2026/04/01"[crdt] : "2026/08/16"[crdt])

Platform adaptation: during the PubMed update, "Intestinal Metaplasia" was not relied upon as a MeSH term; free-text terms such as intestinal metaplasia[Title/Abstract] were retained.

## 3.2 2. Web of Science Core Collection

Update implementation: the original Web of Science strategy was rerun in full and 2026 records were then filtered.

TS=(

(

"atrophic gastritis"

OR "chronic atrophic gastritis"

OR "intestinal metaplasia"

OR "gastric intestinal metaplasia"

OR "gastric dysplasia"

OR "gastric epithelial dysplasia"

OR "gastric intraepithelial neoplasia"

OR "low-grade intraepithelial neoplasia"

OR "low grade intraepithelial neoplasia"

OR "low-grade dysplasia"

OR "low grade dysplasia"

OR "gastric precancerous lesion*"

OR "gastric premalignant lesion*"

OR "gastric precancerous condition*"

OR "gastric premalignant condition*"

)

AND

(

"chinese herbal medicine"

OR "chinese herbal drug*"

OR "herbal medicine"

OR "herbal formula"

OR "herbal decoction"

OR decoction

OR "chinese patent medicine"

OR "patent medicine"

)

AND

(random* OR randomly OR placebo OR trial)

)

NOT TS=(

"non-atrophic gastritis"

OR "non atrophic gastritis"

OR "superficial gastritis"

OR "chronic non-atrophic gastritis"

OR "chronic non atrophic gastritis"

)

NOT TS=(

acupuncture OR electroacupuncture OR "electro-acupuncture"

OR moxibustion OR acupoint OR acupressure OR auricular

OR "tui na" OR tuina OR massage OR "manual therapy" OR cupping

)

NOT TS=(

"artificial intelligence"

OR "machine learning"

OR "deep learning"

OR "neural network"

OR "prediction model"

OR "predictive model"

OR "risk model"

OR "diagnostic model"

OR nomogram

OR radiomics

OR algorithm*

)

NOT TS=(

rat OR rats OR mouse OR mice OR rabbit OR rabbits OR cell OR cells

)

NOT TS=(

mechanism OR mechanisms OR pathway OR pathways OR molecular

OR apoptosis OR proliferation OR signaling OR "signal transduction"

OR biomarker* OR gene* OR protein* OR "in vitro"

)

Operational record: the complete search was rerun, 2026 records were filtered, and exports used Full Record and Cited References.

## 3.3 3. Cochrane CENTRAL

Update implementation: the original CENTRAL logic was retained. Where the updated interface rejected quoted wildcards, equivalent NEXT syntax was used without changing the search concept.

```
#1 [mh "Gastritis, Atrophic"] OR [mh "Intestinal Metaplasia"]

#2 atrophic gastritis:ti,ab,kw OR chronic atrophic gastritis:ti,ab,kw

#3 intestinal metaplasia:ti,ab,kw
   OR gastric intestinal metaplasia:ti,ab,kw

#4 gastric dysplasia:ti,ab,kw
   OR gastric epithelial dysplasia:ti,ab,kw
   OR gastric intraepithelial neoplasia:ti,ab,kw

#5 low-grade intraepithelial neoplasia:ti,ab,kw
   OR low grade intraepithelial neoplasia:ti,ab,kw
   OR low-grade dysplasia:ti,ab,kw
   OR low grade dysplasia:ti,ab,kw

#6 gastric precancerous lesion*:ti,ab,kw
   OR gastric premalignant lesion*:ti,ab,kw
   OR gastric precancerous condition*:ti,ab,kw
   OR gastric premalignant condition*:ti,ab,kw

#7 #1 OR #2 OR #3 OR #4 OR #5 OR #6

#8 [mh "Drugs, Chinese Herbal"] OR [mh "Plant Preparations"]

#9 chinese herbal medicine:ti,ab,kw
   OR chinese herbal drug*:ti,ab,kw
   OR herbal medicine:ti,ab,kw

#10 herbal formula:ti,ab,kw
    OR herbal decoction:ti,ab,kw
    OR decoction:ti,ab,kw

#11 chinese patent medicine:ti,ab,kw
    OR patent medicine:ti,ab,kw

#12 #8 OR #9 OR #10 OR #11

#13 non-atrophic gastritis:ti,ab,kw
    OR non atrophic gastritis:ti,ab,kw
    OR superficial gastritis:ti,ab,kw
    OR chronic non-atrophic gastritis:ti,ab,kw
    OR chronic non atrophic gastritis:ti,ab,kw

#14 acupuncture:ti,ab,kw
    OR electroacupuncture:ti,ab,kw
    OR electro-acupuncture:ti,ab,kw
    OR moxibustion:ti,ab,kw
    OR acupoint:ti,ab,kw
    OR acupressure:ti,ab,kw
    OR auricular:ti,ab,kw
    OR tui na:ti,ab,kw
    OR tuina:ti,ab,kw
    OR massage:ti,ab,kw
    OR manual therapy:ti,ab,kw
    OR cupping:ti,ab,kw

#15 artificial intelligence:ti,ab,kw
    OR machine learning:ti,ab,kw
    OR deep learning:ti,ab,kw
    OR neural network:ti,ab,kw
    OR prediction model:ti,ab,kw
    OR predictive model:ti,ab,kw
    OR risk model:ti,ab,kw
    OR diagnostic model:ti,ab,kw
    OR nomogram:ti,ab,kw
    OR radiomics:ti,ab,kw
    OR algorithm*:ti,ab,kw

#16 #7 AND #12
#17 #16 NOT #13
#18 #17 NOT #14
#19 #18 NOT #15
```

Platform adaptation: Date added was restricted to 2026-04-01 through 2026-08-16. Where quoted wildcards produced syntax errors, equivalent NEXT combinations were used.

## 3.4 4. Embase

Update implementation: the original Embase strategy was retained.

(

'atrophic gastritis'/exp

OR 'intestinal metaplasia'/exp

OR 'gastric dysplasia'/exp

OR 'atrophic gastritis':ti,ab

OR 'chronic atrophic gastritis':ti,ab

OR 'intestinal metaplasia':ti,ab

OR 'gastric intestinal metaplasia':ti,ab

OR 'gastric dysplasia':ti,ab

OR 'gastric epithelial dysplasia':ti,ab

OR 'gastric intraepithelial neoplasia':ti,ab

OR 'low-grade intraepithelial neoplasia':ti,ab

OR 'low grade intraepithelial neoplasia':ti,ab

OR 'low-grade dysplasia':ti,ab

OR 'low grade dysplasia':ti,ab

OR 'gastric precancerous lesion*':ti,ab

OR 'gastric premalignant lesion*':ti,ab

OR 'gastric precancerous condition*':ti,ab

OR 'gastric premalignant condition*':ti,ab

)

AND

(

'chinese herbal drug'/exp

OR 'herbal medicine'/exp

OR 'chinese herbal medicine':ti,ab

OR 'chinese herbal drug*':ti,ab

OR 'herbal medicine':ti,ab

OR 'herbal formula':ti,ab

OR 'herbal decoction':ti,ab

OR decoction:ti,ab

OR 'chinese patent medicine':ti,ab

OR 'patent medicine':ti,ab

)

AND

(

'randomized controlled trial'/exp

OR random*:ti,ab

OR randomly:ti,ab

OR placebo:ti,ab

)

NOT

(

review:it

OR 'systematic review':ti,ab

OR 'meta analysis':ti,ab

)

NOT

(

'non-atrophic gastritis':ti,ab

OR 'non atrophic gastritis':ti,ab

OR 'superficial gastritis':ti,ab

OR 'chronic non-atrophic gastritis':ti,ab

OR 'chronic non atrophic gastritis':ti,ab

)

NOT

(

acupuncture:ti,ab

OR electroacupuncture:ti,ab

OR 'electro-acupuncture':ti,ab

OR moxibustion:ti,ab

OR acupoint:ti,ab

OR acupressure:ti,ab

OR auricular:ti,ab

OR 'tui na':ti,ab

OR tuina:ti,ab

OR massage:ti,ab

OR 'manual therapy':ti,ab

OR cupping:ti,ab

)

NOT

(

rat:ti,ab OR rats:ti,ab

OR mouse:ti,ab OR mice:ti,ab

OR rabbit:ti,ab OR rabbits:ti,ab

OR cell:ti,ab OR cells:ti,ab

)

NOT

(

'artificial intelligence':ti,ab

OR 'machine learning':ti,ab

OR 'deep learning':ti,ab

OR 'neural network':ti,ab

OR 'prediction model':ti,ab

OR 'predictive model':ti,ab

OR 'risk model':ti,ab

OR 'diagnostic model':ti,ab

OR nomogram:ti,ab

OR radiomics:ti,ab

OR algorithm*:ti,ab

)

NOT

(

mechanism:ti,ab

OR mechanisms:ti,ab

OR pathway:ti,ab

OR pathways:ti,ab

OR molecular:ti,ab

OR apoptosis:ti,ab

OR proliferation:ti,ab

OR signaling:ti,ab

OR 'signal transduction':ti,ab

OR biomarker*:ti,ab

OR gene*:ti,ab

OR protein*:ti,ab

OR 'in vitro':ti,ab

)

Platform adaptation: the Emtree syntax 'atrophic gastritis'/exp was explicitly verified during the update.

## 3.5 5. CNKI (China National Knowledge Infrastructure)

Update implementation: the original Chinese disease + Chinese herbal medicine + randomised/clinical-trial logic was retained in title/keyword/abstract fields and filtered to 2026.

(

胃癌前病变

OR 慢性萎缩性胃炎

OR 胃黏膜萎缩

OR 肠上皮化生

OR 胃肠上皮化生

OR 胃异型增生

OR 胃黏膜异型增生

OR 胃上皮内瘤变

OR 胃低级别上皮内瘤变

)

AND

(

中药

OR 中药治疗

OR 中西医结合

OR 中药联合西药

OR 中药加用西药

)

AND

(

随机

OR 随机对照

OR 随机分组

OR 临床试验

)

Implementation note: CNKI Advanced Search used the original TI/KY/AB field logic; the original exclusion logic for animal, mechanism, review/meta-analysis, cervical disease, acupuncture and related records was retained.

## 3.6 6. Wanfang Data

主题:

(

(胃癌前病变

OR 慢性萎缩性胃炎

OR 胃黏膜萎缩

OR 肠上皮化生

OR 胃肠上皮化生

OR 胃异型增生

OR 胃黏膜异型增生

OR 胃上皮内瘤变

OR 胃低级别上皮内瘤变)

AND

(中药

OR 中药治疗

OR 中西医结合

OR 中药联合西药

OR 中药加用西药)

AND

(随机

OR 随机对照

OR 随机分组

OR 临床试验)

)

NOT 主题:(小鼠 OR 大鼠 OR 动物实验 OR 细胞 OR 细胞实验)

NOT 主题:(机制 OR 作用机制 OR 分子机制 OR 信号通路)

NOT 主题:(Meta分析 OR 荟萃分析 OR 系统评价 OR 系统综述 OR 综述)

NOT 主题:(宫颈 OR 宫颈上皮内瘤变 OR CIN)

NOT 主题:(

针灸 OR 电针 OR 艾灸 OR 穴位贴敷 OR 穴位注射

OR 耳穴压豆 OR 耳穴 OR 耳穴贴压

OR 穴位埋线 OR 穴位埋针 OR 推拿

OR 按摩 OR 拔罐 OR 刮痧 OR 穴位刺激 OR 梅花针

)

Operational record: records were filtered to 2026 and exported.

## 3.7 7. VIP (Chinese Science and Technology Journal Database)

(

(T=胃癌前病变 OR K=胃癌前病变 OR R=胃癌前病变)

OR

(T=慢性萎缩性胃炎 OR K=慢性萎缩性胃炎 OR R=慢性萎缩性胃炎)

OR

(T=胃黏膜萎缩 OR K=胃黏膜萎缩 OR R=胃黏膜萎缩)

OR

(T=肠上皮化生 OR K=肠上皮化生 OR R=肠上皮化生)

OR

(T=胃肠上皮化生 OR K=胃肠上皮化生 OR R=胃肠上皮化生)

OR

(T=胃异型增生 OR K=胃异型增生 OR R=胃异型增生)

OR

(T=胃黏膜异型增生 OR K=胃黏膜异型增生 OR R=胃黏膜异型增生)

OR

(T=胃上皮内瘤变 OR K=胃上皮内瘤变 OR R=胃上皮内瘤变)

OR

(T=胃低级别上皮内瘤变 OR K=胃低级别上皮内瘤变 OR R=胃低级别上皮内瘤变)

)

AND

(

(T=中药 OR K=中药 OR R=中药)

OR

(T=中药治疗 OR K=中药治疗 OR R=中药治疗)

OR

(T=中西医结合 OR K=中西医结合 OR R=中西医结合)

OR

(T=中药联合西药 OR K=中药联合西药 OR R=中药联合西药)

OR

(T=中药加用西药 OR K=中药加用西药 OR R=中药加用西药)

)

AND

(

(T=随机 OR K=随机 OR R=随机)

OR

(T=随机对照 OR K=随机对照 OR R=随机对照)

OR

(T=随机分组 OR K=随机分组 OR R=随机分组)

OR

(T=临床试验 OR K=临床试验 OR R=临床试验)

)

Implementation note: the original NOT blocks for animals, mechanisms, reviews/meta-analyses, cervical disease, acupuncture and related records were retained; the update was filtered to 2026.

## 3.8 8. SinoMed (Chinese Biomedical Literature Database)

(

胃癌前病变

OR 慢性萎缩性胃炎

OR 胃黏膜萎缩

OR 肠上皮化生

OR 胃肠上皮化生

OR 胃异型增生

OR 胃黏膜异型增生

OR 胃上皮内瘤变

OR 胃低级别上皮内瘤变

)

AND

(

中药

OR 中药治疗

OR 中西医结合

OR 中药联合西药

OR 中药加用西药

)

AND

(

随机

OR 随机对照

OR 随机分组

OR 临床试验

)

NOT

(

小鼠 OR 大鼠 OR 动物实验 OR 细胞 OR 细胞实验

)

NOT

(

针灸 OR 电针 OR 艾灸 OR 穴位贴敷

OR 穴位注射 OR 耳穴压豆 OR 耳穴

OR 推拿 OR 按摩 OR 拔罐 OR 刮痧

)

NOT

(

Meta分析

OR 荟萃分析

OR 系统评价

OR 系统综述

OR 综述

OR 网络药理

OR 网络药理学

OR 数据挖掘

OR 知识图谱

OR 研究进展

)

Operational record: the 2026 update identified one record: "柴归汤辅助治疗气虚血瘀型慢性萎缩性胃炎36例临床研究".

## 3.9 9. ClinicalTrials.gov

(

"atrophic gastritis"

OR "intestinal metaplasia"

OR "gastric dysplasia"

OR "gastric intraepithelial neoplasia"

)

AND

(

"Chinese herbal medicine"

OR herbal

OR decoction

)

Operational record: no date or status restriction was applied. Four records were retrieved: NCT05388890, NCT06324825, NCT00920712, and NCT05808010.

## 3.10 10. WHO ICTRP

Update implementation: consistent with the original strategy, separate title-term searches were used rather than a complex Boolean expression.

atrophic gastritis

intestinal metaplasia

gastric dysplasia

gastric precancerous lesions

Operational record: no date or status restriction was applied; results from the four searches were deduplicated by TrialID, yielding 145 unique Trial IDs.

## 3.11 11. ChiCTR (Chinese Clinical Trial Registry)

Update implementation: disease terms were searched separately in Public title and Scientific title; status was set to All and no date restriction was applied.

慢性萎缩性胃炎

胃癌前病变

胃黏膜萎缩

肠上皮化生

胃异型增生

胃上皮内瘤变

低级别异型增生

低级别上皮内瘤变

chronic atrophic gastritis

gastric precancerous lesions

intestinal metaplasia

gastric dysplasia

gastric intraepithelial neoplasia

Intervention-field manual screening terms:

中药

中医药

中药复方

中药汤剂

中成药

中西医结合

中药联合西药

Chinese herbal medicine

herbal medicine

decoction

Deduplication rule: final results were deduplicated by ChiCTR registration number; hit counts from the 13 separate searches were not summed.

# 4 Frozen principles for the revision-stage search audit

The search strategy was not redesigned: the update was based on the originally submitted Supplementary Appendix 1 and records only the update searches actually executed and necessary platform-syntax adaptations.

Bibliographic databases primarily used a 2026 update window. PubMed and CENTRAL used explicit 2026-04-01 to 2026-08-16 limits; Chinese databases were filtered to 2026.

ClinicalTrials.gov, WHO ICTRP, and ChiCTR were rerun across the full registry without date restrictions.

The CENTRAL NEXT substitution was a platform-syntax adaptation rather than a change in search concepts.

Embase used the verified Emtree term 'atrophic gastritis'/exp.

Registry results were deduplicated by registration identifier; in particular, results from repeated WHO ICTRP and ChiCTR keyword searches were not summed.

For the revision record, original searches (through 31 March 2026) and updated searches (through 16 August 2026) are reported separately.

# 5 Recommended search-log fields
