# Supplementary material for: Chinese herbal medicine combined with conventional therapy for gastric precancerous lesions: a systematic review and meta-analysis
Source: Oncol Rev. 2026 Oct 19;20:1947905. doi: 10.3389/or.2026.1947905 (PMC13724460; doi:10.3389/or.2026.1947905)
Supplement: Supplementary file 4 [file Supplementaryfile2.docx]

Supplementary Methods and Audit Tables

Final 69-study evidence-base audit

Date: 23 August 2026

Status of these supplementary audit tables

These integrated supplementary audit tables provide the current source-verified study identity, pooled estimates, sensitivity memberships, small-study-effect assessments, GRADE judgements, and protocol/analysis-decision documentation for the 69-study evidence base. Historical screening counts are preserved where needed for audit transparency, while current retrieval status is reported separately. A companion intervention-reproducibility workbook provides source-reported composition/dose evidence; Supplementary Table S1 separately provides editorially standardised botanical/Latin names.

# SR1. Search update and PRISMA eligibility audit

The final integrated PRISMA 2020 flow reports the final retrieval status: 3,080 database records plus 15 registry records; 1,265 duplicates removed; 1,830 records screened; 1,267 records excluded; 563 reports sought; 11 reports remained unavailable after additional retrieval attempts; 552 reports assessed; 483 full-text exclusions (454 historical/original, 15 later-retrieved historical reports assessed and found ineligible, and 14 update-search exclusions); and 69 included independent studies. The historical snapshot of 26 reports originally not retrieved is retained separately in SR13, where 15 are documented as subsequently obtained and assessed and 11 as remaining unavailable.

| Stage | Count | Arithmetic/source | Audit interpretation |
| --- | --- | --- | --- |
| Database records identified | 3,080 | 3,052 original + 28 update records | Combined original + update searches |
| Registry records identified | 15 | Trial registries | No additional eligible registry-only trial |
| Duplicates removed | 1,265 | 3,095 - 1,830 | Integrated final flow |
| Records screened | 1,830 | 3,095 - 1,265 | Title/abstract screening |
| Records excluded | 1,267 | 1,830 - 563 | Title/abstract exclusions |
| Reports sought for retrieval | 563 | 1,830 - 1,267 | Integrated original + update flow |
| Reports not retrieved at final status | 11 | 11 unavailable | Final retrieval status |
| Reports assessed for eligibility | 552 | 563 - 11 | Integrated final full-text assessment |
| Full-text reports excluded | 483 | 454 historical/original + 15 later-retrieved historical + 14 update | One primary exclusion reason per report is in Supplementary Data 1 |
| Studies included in review | 69 | 552 - 483 | 47 retained from the prior eligible set + 20 historically missed + 2 update-eligible |
| Historical not-retrieved snapshot | 26 | 15 later obtained/assessed + 11 unavailable | Historical screening state preserved separately in SR13 |

# SR2. Final 69-study evidence-base characteristics and intervention reproducibility

Companion source-level intervention audit: Supplementary Data 2 (Intervention Reproducibility) preserves source-reported formula composition and dose text where reliably extractable from the supplied full text and records preparation, modification, and manufacturer/quality-control fields, retaining NR when information was not reported or not reliably recoverable. Supplementary Table S1 provides detailed characteristics of all 69 included studies across Panels A-E. In Panel C, Chinese ingredient names and quantities are source-reported, whereas the dedicated 'Botanical/Latin name (editorially standardized)' field was standardized from those source-reported Chinese terms using the 2025 Chinese Pharmacopoeia and accepted taxonomic nomenclature. These botanical/Latin names were not necessarily printed verbatim in the original reports. NR was retained when composition was not reported or not reliably recoverable; no undocumented component, dose, manufacturer, or quality-control information was reconstructed.

NR means not reported in the source or not reliably recoverable from the source audit. No botanical composition, manufacturer, batch, or quality-control detail was inferred from external knowledge.

| **Study** | **Author / year** | **Disease / population** | **Randomised T/C** | **Experimental intervention** | **Control** | **Duration** | **Preparation / formulation** | **Modification** | **Manufacturer / QC** |
| --- | --- | --- | --- | --- | --- | --- | --- | --- | --- |
| S1 | Wei / 2025 | CAG | 40/40 | Modified Banxia Xiexin decoction + conventional therapy | conventional therapy | 4 weeks | NR | Fixed or NR | NR |
| S2 | Xi / 2025 | CAG with IM | 30/30 | Syndrome-differentiated Chinese herbal medicine + conventional therapy | conventional therapy | 3 months | NR | Modified/individualized | NR |
| S3 | Zhang / 2022 | CAG | 35/35 | Modified Buxu Yangwei decoction + conventional therapy | conventional therapy | 3 months | NR | Modified/individualized | NR |
| S4 | Shen / 2017 | PLGC | 60/60 | Shenzhi Xiaowei decoction + teprenone | Teprenone | 4 months | NR | Fixed or NR | NR |
| S5 | Yuan / 2026 | CAG | 60/60 | Modified Chaihu Jia Longgu Muli decoction + omeprazole and colloidal bismuth pectin | Omeprazole and colloidal bismuth pectin | 8 weeks | NR | Fixed or NR | NR |
| S6 | You / 2021 | PLGC | 35/33 | Chaipu decoction + Huatan Xiaoyu formula + conventional therapy | conventional therapy | 6 months | NR | Fixed or NR | NR |
| S7 | Gao / 2022 | PLGC | 40/40 | Fuwei Kangyi decoction + Weimeisu capsules | Weimeisu capsules | 3 months | NR | Fixed or NR | NR |
| S8 | Yi / 2022 | CAG with dysplasia | 35/35 | Fuwei Xiaowei decoction granules + conventional therapy | conventional therapy | 8 weeks | NR | Fixed or NR | NR |
| S9 | Zhang / 2016 | CAG with bile reflux | 62/65 | Modified Huatan Xiaoyu formula + ursodeoxycholic acid and hydrotalcite | Ursodeoxycholic acid + hydrotalcite | 24 weeks | NR | Modified/individualized | NR |
| S10 | Zhang / 2020 | CAG with erosion | 50/50 | Modified Huatan Xiaoyu formula + conventional therapy | conventional therapy | 6 months | NR | Modified/individualized | NR |
| S11 | Huang / 2020 | H. pylori-positive CAG | 45/45 | Huangqi Jianzhong decoction + Liangfu pills + conventional therapy | conventional therapy | 4 weeks | NR | Fixed or NR | NR |
| S13 | Liu / 2005 | CAG | 50/50 | Guanxinning injection + weimeisu tablets + aluminum sulfate tablets | Weimeisu tablets and aluminum sulfate tablets | 14 days + 6-month follow-up | NR | Fixed or NR | NR |
| S14 | Liu / 2017 | CAG | 50/50 | Modified Fuzi Lizhong decoction + teprenone | Teprenone | 4 months | NR | Fixed or NR | NR |
| S15 | Wang / 2025 | H. pylori-related CAG | 59/59 | Modified Sanren decoction + triple therapy | Triple therapy | 10 days | Decoction | Fixed or NR | NR |
| S16 | Zhuang / 2011 | CAG | 50/50 | Jianpi Huatan formula + conventional therapy | conventional therapy | 3 months | NR | Modified/individualized | NR |
| S17 | Wang / 2021 | CAG | 60/60 | Jianpi Huatan Chinese herbal medicine + conventional therapy | conventional therapy | 3 months | NR | Modified/individualized | NR |
| S18 | Zhang / 2013 | CAG | 30/30 | Jianpi Huoxue formula + conventional therapy | conventional therapy | 8 weeks | NR | Modified/individualized | NR |
| S19 | Shen / 2021 | CAG | 49/49 | Jianpi Huoxue Xiaoji formula + conventional therapy | conventional therapy | 8 weeks | NR | Fixed or NR | NR |
| S20 | Wang / 2023 | CAG with erosion | 43/43 | Jianpi Liqi Huayu formula + conventional therapy | conventional therapy | 12 weeks | Decoction | Modified/individualized | NR |
| S21 | Zhang / 2025 | H. pylori-positive CAG | 53/53 | Jianpi Tongluo prescription + bismuth quadruple therapy | Bismuth quadruple therapy | 8 weeks | NR | Fixed or NR | NR |
| S22 | Jia / 2021 | H. pylori-positive CAG | 55/55 | Jianpi Yiqi decoction + quadruple therapy | Quadruple therapy | 12 weeks | NR | Fixed or NR | NR |
| S24 | Yang / 2024 | H. pylori-positive CAG | 50/50 | Liqi Huayu Jianwei decoction + quadruple therapy | Quadruple therapy | 8 weeks | NR | Fixed or NR | NR |
| S26 | Wang / 2018 | CAG with primary bile reflux | 42/36 | Liuwei Anxiao capsules + mosapride + hydrotalcite | Mosapride + hydrotalcite | 4 weeks | NR | Fixed or NR | NR |
| S27 | Ning / 2016 | CAG | 55/55 | Manwei San + vitacoenzyme tablets | Vitacoenzyme tablets | 3 months | NR | Fixed or NR | NR |
| S28 | Qi / 2024 | H. pylori-positive CAG | 34/34 | Self-made Chenpi formula + quadruple therapy | Quadruple therapy | 3 months | NR | Fixed or NR | NR |
| S29 | Dong / 2024 | CAG | 43/43 | Piwei Peiyuan decoction + folic acid tablets | Folic acid tablets | 18 weeks | NR | Fixed or NR | NR |
| S30 | Yang / 2017 | H. pylori-positive CAG in older adults | 38/38 | Shugan Hewei Tang + triple therapy | Triple therapy | 2 months | NR | Fixed or NR | NR |
| S31 | Xu / 2018 | H. pylori-positive CAG | 120/120 | Weifuchun tablets + symptomatic conventional therapy | Symptomatic conventional therapy | 2 months | NR | Fixed or NR | NR |
| S32 | Liu / 2010 | CAG with IM | 50/50 | Weishu decoction + H. pylori eradication therapy | H. pylori eradication therapy | 6 months | NR | Fixed or NR | NR |
| S33 | Xing / 2004 | CAG with IM | 93/89 | Weite'an tablets + triple therapy | Triple therapy | 3 months | NR | Fixed or NR | NR |
| S34 | Jiang / 2025 | CAG | 30/30 | Wenyang Jianpi ointment + conventional therapy | conventional therapy | 12 weeks | NR | Fixed or NR | NR |
| S35 | Zhang / 2021 | CAG | 30/30 | Wenyang Jianpi decoction + conventional therapy | conventional therapy | 2 months | NR | Modified/individualized | NR |
| S37 | Bu / 2019 | CAG | 57/57 | Xiaohua Funing decoction + rebamipide | Rebamipide | 3 months | NR | Fixed or NR | NR |
| S38 | Yu / 2023 | PLGC | 37/37 | Yiqi Huoxue prescription + conventional therapy | conventional therapy | 1 month | NR | Fixed or NR | NR |
| S39 | Xu / 2021 | PLGC | 51/51 | Yiwei Huazhuo Jiedu decoction + vitacoenzyme tablets | Vitacoenzyme tablets | 3 months | Hospital decoction | Fixed or NR | Hospital pharmacy |
| S40 | Li / 2019 | CAG | 42/42 | Yiwei Jianpi decoction + conventional therapy | conventional therapy | 8 weeks | NR | Fixed or NR | NR |
| S42 | Sun / 2017 | PLGC | 64/32 | Radix Curcumae granule + folic acid; Notoginseng powder + folic acid | Folic acid | 6 months | NR | Fixed or NR | NR |
| S43 | Wang / 2010 | CAG | 84/42 | Zhenqi Fuzheng capsules + conventional therapy | conventional therapy | 6 months; 3 courses of 2 months with 2–3-week intervals | NR | Fixed or NR | NR |
| S44 | Zhang / 2008 | CAG | 36/34 | Danshen Yin + Chai-Shao-Liujunzi decoction + conventional therapy | conventional therapy | 2–5 courses (30 days/course) | NR | Modified/individualized | NR |
| S45 | Zhang / 2004 | H. pylori-positive CAG | 36/34 | Yangwei decoction + conventional therapy | conventional therapy | 8 weeks | NR | Fixed or NR | NR |
| S46 | Fu / 2020 | CAG | 40/40 | Jianpi Tongluo decoction + conventional therapy | conventional therapy | 6 months | NR | Modified/individualized | NR |
| S47 | Wang / 2003 | H. pylori-related CAG | 58/27 | Xiangsha Liujunzi decoction + conventional therapy | conventional therapy | 6 months | Decoction | Fixed or NR | NR |
| S48 | Sun / 2020 | H. pylori-positive CAG | 120/60 | Modified Yiwei decoction + conventional therapy; Modified Yiwei decoction + folic acid + conventional therapy | conventional therapy | 3 months | NR | Modified/individualized | NR |
| S49 | Wu / 2017 | CAG with IM | 49/49 | Modified Zisheng decoction + famotidine | Famotidine | 30 days | NR | Modified/individualized | NR |
| S50 | Lin / 2023 | CAG | 40/40 | Self-formulated Baidan Weiwei decoction + omeprazole | Omeprazole | 3 months | NR | Fixed or NR | NR |
| S51 | Wang / 2021 | CAG | 55/55 | Self-formulated Tiaowei decoction + conventional therapy | conventional therapy | 4 weeks | NR | Modified/individualized | NR |
| S52 | Zhang / 2023 | H. pylori-positive chronic active gastritis with atrophy | 38/38 | Weiwei granules + quadruple therapy | Quadruple therapy | 6 months; two 3-month courses | NR | Fixed or NR | NR |
| H01 | Cai / 2016 | NR | 40/40 | Shugan Hewei formula + vitacoenzyme | Vitacoenzyme | 3 months | Decoction | Modified/individualized | NR |
| H02 | Shen / 2007 | NR | 69/63 | NR | NR | 3 months | NR | Fixed or NR | NR |
| H03 | Ding / 2019 | NR | 51/52 | Nuangan decoction + triple therapy | Triple therapy | 30 days | Decoction | Fixed or NR | NR |
| H04 | Du / 2015 | NR | 48/48 | NR | NR | 8 weeks | NR | Fixed or NR | NR |
| H05 | Feng / 2011 | NR | 54/51 | Moluodan + folic acid | Matching TCM placebo + folic acid | 3 months | Patent pill | Fixed or NR | Handan Pharmaceutical; QC: Product approval number reported |
| H06 | Guo / 2023 | NR | 45/45 | NR | NR | NR | NR | Fixed or NR | NR |
| H07 | Hu / 2016 | NR | 40/40 | NR | NR | 4 months | NR | Fixed or NR | NR |
| H08 | Hu / 2019 | NR | 60/60 | NR | NR | 3 months | NR | Fixed or NR | NR |
| H09 | Jin / 2012 | NR | 73/61 | NR | NR | 12 weeks | NR | Fixed or NR | NR |
| H10 | Ke / 2025 | NR | 35/35 | Weixu Yuzu prescription + conventional therapy | Conventional therapy | 3 months | Hospital decoction | Fixed or NR | Dehua County TCM Hospital decoction room |
| H11 | Peng / 2022 | NR | 89/89 | Shengyang Yiwei decoction + quadruple therapy | Quadruple therapy | 3 months | Decoction | Fixed or NR | NR |
| H12 | Wang / 2012 | NR | 42/41 | Qifang Weitong granules + conventional treatment | Conventional treatment | 8 weeks | Dispensing granules | Fixed or NR | Jiangyin Tianjiang Pharmaceutical |
| H13 | Xiang / 2007 | NR | 80/50 | Wushen decoction + sucralfate/mosapride/folic acid | Sucralfate + mosapride + folic acid | 3 months | Decoction | Fixed or NR | NR |
| H14 | Xu / 2021 | NR | 53/53 | Wanfu Qutong decoction + esomeprazole | Esomeprazole | 3 months | Decoction | Fixed or NR | NR |
| H15 | Ye / 2024 | NR | 40/40 | Yiweining capsules + rebamipide | Rebamipide | 12 weeks | Hospital capsule | Fixed or NR | Second People's Hospital Affiliated to Fujian University of TCM pharmacy |
| H16 | Ye / 2020 | NR | 100/100 | Erchen Pingwei powder decoction + conventional therapy | Conventional therapy | 24 weeks | Decoction | Fixed or NR | NR |
| H17 | Zhu / 2009 | NR | 28/22 | Yangwei granules + multienzyme + vitamin B6 | Multienzyme + vitamin B6 | 6 months | Granules | Fixed or NR | Hangzhou Zhengda Qingchunbao |
| H18 | Zhu / 2010 | NR | 30/30 | NR | NR | 6 treatment courses; exact duration unclear | NR | Fixed or NR | NR |
| H19 | Zhu / 2021 | NR | 35/35 | NR | NR | 12 weeks | NR | Fixed or NR | NR |
| H20 | Zou / 2024 | CAG with moderate-to-severe atrophy and/or IM, with/without low-grade dysplasia; H. pylori negative | 84/168 | Moluodan + folic acid | Folic acid | 12 months | Patent medicine sachet | Fixed or NR | Handan Pharmaceutical Co., Ltd.; QC: blinded simulants and approved product reported |
| U02 | Xu / 2026 | NR | 38/38 | Chaigui decoction + teprenone + folic acid | Teprenone + folic acid | 12 weeks | Hospital decoction | Fixed or NR | Wuxi Xishan District TCM Hospital pharmacy |
| U04 | Wang / 2026 | NR | 50/50 | Shuwei formula + Deanxit + mucosal protection | Deanxit + mucosal protection | 12 weeks | Hospital decoction | Fixed or NR | NR |

# SR3. Result-level RoB 2 summary for audited outcome families

Companion source-level audits: Supplementary Data 3 (Full RoB 2 Signalling-Question Audit) now provides complete signalling-question responses, result-level domain judgements, source locators, and study-specific rationales for all 102 final result/outcome-family assessments. The previous 87-row audit was supplemented with 15 secondary result/outcome-family records that were already present in the final analysis lock but lacked full signalling-question rows; the added records reproduce the locked domain and overall judgements and do not alter outcome membership or pooled effects. Among the original 87 rows, the RoB 2 rebuild identified domain-level changes in six result/outcome-family assessments (eight individual domain cells) but no overall RoB 2 judgement. Supplementary Data 4 (Denominator and Analysis-Population Audit) reports randomised, completed/endpoint, analysed, pathology, lesion-positive, H. pylori, and safety denominators and identifies ITT/FAS/PPS/completer-type analyses where supported by the source. A subsequent final statistical-method quality check replaced conventional Egger testing for SMD outcomes with the SMD-specific Pustejovsky-Rodgers test. The principal pooled estimates were unchanged, but publication/dissemination-bias judgements and GRADE were re-adjudicated accordingly. Supplementary Data 5 provides the study-level forest plots and method-corrected contour-enhanced funnel plots for the five principal outcomes. Supplementary Data 6 provides the active method-corrected R scripts, exported inputs, control workbooks and validation targets, while superseded Phase 6 outputs are retained only as clearly labelled historical audit files. Exact software versions used for the earlier archived analyses are not retrospectively inferred because the preserved archive did not contain sessionInfo() output or a package lockfile. The final method-corrected Pustejovsky-Rodgers analyses and corrected dysplasia/IEN baseline-balanced sensitivity were independently rerun and verified in R 4.5.2 with meta version 8.2-1; sessionInfo(), inputs, logs, and outputs are archived in Supplementary Data 6.

| **Study** | **Outcome family** | **D1** | **D2** | **D3** | **D4** | **D5** | **Overall** | **Key rationale** |
| --- | --- | --- | --- | --- | --- | --- | --- | --- |
| H06 | Lesion-specific continuous pathology | Some concerns | Some concerns | Low | Some concerns | Some concerns | Some concerns | Scale-result inconsistency handled as data-quality/strict-comparability issue; not automatically upgraded to D4 High. |
| H08 | Lesion-specific continuous pathology | Some concerns | Some concerns | Low | Some concerns | Some concerns | Some concerns | All reported data forms retained; no outcome selected by statistical significance. |
| H14 | Lesion-specific continuous pathology | Some concerns | Some concerns | Low | Some concerns | Some concerns | Some concerns | All reported data forms retained; no outcome selected by statistical significance. |
| S1 | Lesion-specific continuous pathology | Some concerns | Some concerns | Low | Some concerns | Some concerns | Some concerns | Treatment group: Modified Banxia Xiexin decoction + conventional therapy; control group: conventional therapy; post-treatment values extracted \| Frozen adjudication: study-reported lesion statistics retained without rescaling; strict-comparability analysis excluded. |
| S2 | Lesion-specific continuous pathology | Some concerns | Some concerns | Low | Some concerns | Some concerns | Some concerns | Treatment group: Syndrome-differentiated Chinese herbal medicine + conventional therapy; control group: conventional therapy; post-treatment values extracted |
| S3 | Lesion-specific continuous pathology | Some concerns | Some concerns | Low | Some concerns | Some concerns | Some concerns | Treatment group: Modified Buxu Yangwei decoction + conventional therapy; control group: conventional therapy; post-treatment values extracted |
| S4 | Lesion-specific continuous pathology | Some concerns | Some concerns | Low | Some concerns | Some concerns | Some concerns | Treatment group: Shenzhi Xiaowei decoction + Teprenone; control group: Teprenone; post-treatment values extracted |
| S5 | Lesion-specific continuous pathology | Some concerns | Some concerns | Low | Some concerns | Some concerns | Some concerns | Treatment group: Modified Chaihu Jia Longgu Muli decoction + Omeprazole and colloidal bismuth pectin; control group: Omeprazole and colloidal bismuth pectin; post-treatment values extracted |
| S7 | Lesion-specific continuous pathology | Some concerns | Some concerns | Low | Some concerns | Some concerns | Some concerns | Treatment group: Fuwei Kangyi decoction + Weimeisu; control group: Weimeisu; post-treatment values extracted |
| S8 | Lesion-specific continuous pathology | Some concerns | Some concerns | Low | Some concerns | Some concerns | Some concerns | Treatment group: Fuwei Xiaowei decoction + conventional therapy; control group: conventional therapy; post-treatment values extracted |
| S10 | Lesion-specific continuous pathology | Some concerns | Some concerns | Low | Some concerns | Some concerns | Some concerns | Treatment group: Modified Huatan Xiaoyu formula + conventional therapy; control group: conventional therapy; post-treatment values extracted |
| S15 | Lesion-specific continuous pathology | Some concerns | Some concerns | Low | Some concerns | Some concerns | Some concerns | Treatment group: Modified Sanren decoction + triple therapy; control group: triple therapy; post-treatment values extracted |
| S19 | Lesion-specific continuous pathology | Some concerns | Some concerns | Low | Some concerns | Some concerns | Some concerns | Treatment group: Jianpi Huoxue Xiaoji formula + conventional therapy; control group: conventional therapy; post-treatment values extracted |
| S20 | Lesion-specific continuous pathology | High | Some concerns | Low | Some concerns | Some concerns | High | Multiple strong directionally consistent baseline pathology imbalances plus conflict with generic baseline P>0.05 statement; allocation concealment not reported. |
| S21 | Lesion-specific continuous pathology | Some concerns | Some concerns | Low | Some concerns | Some concerns | Some concerns | Original item: 腺体萎缩. |
| S22 | Lesion-specific continuous pathology | Some concerns | Some concerns | Low | Some concerns | Some concerns | Some concerns | Original item: 萎缩. |
| S24 | Lesion-specific continuous pathology | Some concerns | Some concerns | Low | Some concerns | Some concerns | Some concerns | Original item: 腺体萎缩. |
| S27 | Lesion-specific continuous pathology | Some concerns | Some concerns | Low | Some concerns | Some concerns | Some concerns | Original item: 腺体减少. |
| S28 | Lesion-specific continuous pathology | Some concerns | Some concerns | Low | Some concerns | Some concerns | Some concerns | Original item: 萎缩. |
| S29 | Lesion-specific continuous pathology | Some concerns | Some concerns | Low | Some concerns | Some concerns | Some concerns | Original item: 萎缩. |
| S31 | Lesion-specific continuous pathology | Some concerns | Some concerns | Low | Some concerns | Some concerns | Some concerns | Original item: 萎缩. \| Preserve reported values and source wording; no silent correction of the scale text. |
| S37 | Lesion-specific continuous pathology | Some concerns | Some concerns | Low | Some concerns | Some concerns | Some concerns | Original item: 腺体萎缩. |
| S38 | Lesion-specific continuous pathology | Some concerns | Some concerns | Low | Some concerns | Some concerns | Some concerns | Original item: 萎缩. |
| S39 | Lesion-specific continuous pathology | Some concerns | Some concerns | Low | Some concerns | Some concerns | Some concerns | Original item: 黏膜萎缩. \| Full-text verification completed. The article defines pathology-variable scores: atrophy/IM/dysplasia 0/3/6/9 and chronic inflammation/activity 0/1/2/3. The supplied full text does NOT report the previously migrated five-site biopsy scheme, five specimens, or two pathologists for the pathology score. Only the Ki-67 method explicitly states gastroscopic gastric-mucosa tissue collection. Table 2 prints control baseline chronic-inflammation SD=2.88 on a bounded 0–3 scale; this is a source-reported mathematical inconsistency and must be retained verbatim, not corrected. |
| S40 | Lesion-specific continuous pathology | Some concerns | Some concerns | Low | Some concerns | Some concerns | Some concerns | Original item: 胃黏膜萎缩积分. |
| S42 | Lesion-specific continuous pathology | Some concerns | Some concerns | Some concerns | Some concerns | Some concerns | Some concerns | 12/96 randomized participants missing from pathology analysis; reasons and missing-data handling not reported. |
| S46 | Lesion-specific continuous pathology | Some concerns | Some concerns | Low | Some concerns | Some concerns | Some concerns | Original item: 萎缩. |
| S50 | Lesion-specific continuous pathology | Some concerns | Some concerns | Low | Some concerns | Some concerns | Some concerns | Original item: 萎缩. |
| S51 | Lesion-specific continuous pathology | Some concerns | Some concerns | Low | Some concerns | Some concerns | Some concerns | Scale-result inconsistency handled as data-quality/strict-comparability issue; not automatically upgraded to D4 High. |
| S52 | Lesion-specific continuous pathology | Some concerns | Some concerns | Low | Some concerns | Some concerns | Some concerns | Original item: atrophy. |
| S49 | Lesion-specific continuous pathology | Some concerns | Some concerns | Some concerns | Some concerns | Some concerns | Some concerns | FAS/PPS and prose/table group-label denominator inconsistency; table-based 46 vs 47 retained. |
| S18 | Binary pathology | Some concerns | Some concerns | Low | Some concerns | Some concerns | Some concerns | Allocation concealment/blinding/protocol reporting generally incomplete; no domain-level evidence supporting High risk identified. |
| U04 | Binary pathology | Some concerns | Some concerns | Low | Low | Some concerns | Some concerns | Same pathologist and assessor explicitly blinded to group assignment; all 50/50 analysed. |
| S33 | Binary pathology | Some concerns | Some concerns | Low | Some concerns | Some concerns | Some concerns | Allocation concealment/blinding/protocol reporting generally incomplete; no domain-level evidence supporting High risk identified. |
| S13 | Binary pathology | Some concerns | Some concerns | Low | Some concerns | Some concerns | Some concerns | Allocation concealment/blinding/protocol reporting generally incomplete; no domain-level evidence supporting High risk identified. |
| S32 | Binary pathology | Some concerns | Some concerns | Low | Some concerns | Some concerns | Some concerns | Allocation concealment/blinding/protocol reporting generally incomplete; no domain-level evidence supporting High risk identified. |
| S9 | Binary pathology | Some concerns | Some concerns | Low | Some concerns | Some concerns | Some concerns | Allocation concealment/blinding/protocol reporting generally incomplete; no domain-level evidence supporting High risk identified. |
| S16 | Binary pathology | Some concerns | Some concerns | Low | Some concerns | Some concerns | Some concerns | Allocation concealment/blinding/protocol reporting generally incomplete; no domain-level evidence supporting High risk identified. |
| S17 | Binary pathology | Some concerns | Some concerns | Low | Some concerns | Some concerns | Some concerns | Allocation concealment/blinding/protocol reporting generally incomplete; no domain-level evidence supporting High risk identified. |
| H07 | Binary pathology | Some concerns | Some concerns | Low | Some concerns | Some concerns | Some concerns | Allocation concealment/blinding/protocol reporting generally incomplete; no domain-level evidence supporting High risk identified. |
| S43 | Binary pathology | Some concerns | Some concerns | Low | Some concerns | Some concerns | Some concerns | Allocation concealment/blinding/protocol reporting generally incomplete; no domain-level evidence supporting High risk identified. |
| H19 | Binary pathology | Some concerns | Some concerns | Low | Some concerns | Some concerns | Some concerns | Allocation concealment/blinding/protocol reporting generally incomplete; no domain-level evidence supporting High risk identified. |
| S47 | Binary pathology | Some concerns | Some concerns | Some concerns | Some concerns | Some concerns | Some concerns | Three baseline IM-positive control participants absent from response denominator without explanation. |
| S14 | Binary pathology | Some concerns | Some concerns | Some concerns | Some concerns | Some concerns | Some concerns | Randomized 50/50; 4/6 participants dropped out and only 46/44 were analysed. Reasons for dropout were not reported. Allocation concealment, assessor blinding, and an auditable pre-specified analysis plan were not reported. |
| H03 | H. pylori | Some concerns | Some concerns | Low | Some concerns | Some concerns | Some concerns | Allocation concealment/blinding/protocol reporting generally incomplete; no domain-level evidence supporting High risk identified. |
| H04 | H. pylori | Some concerns | Some concerns | Some concerns | Some concerns | Some concerns | Some concerns | Allocation concealment/blinding/protocol reporting generally incomplete; no domain-level evidence supporting High risk identified. |
| H05 | H. pylori | Some concerns | Some concerns | Low | Some concerns | Some concerns | Some concerns | Allocation concealment/blinding/protocol reporting generally incomplete; no domain-level evidence supporting High risk identified. |
| H11 | H. pylori | Some concerns | Some concerns | Low | Some concerns | Some concerns | Some concerns | Allocation concealment/blinding/protocol reporting generally incomplete; no domain-level evidence supporting High risk identified. |
| H12 | H. pylori | Some concerns | Some concerns | Low | Some concerns | Some concerns | Some concerns | Allocation concealment/blinding/protocol reporting generally incomplete; no domain-level evidence supporting High risk identified. |
| H13 | H. pylori | Some concerns | Some concerns | Low | Some concerns | Some concerns | Some concerns | Allocation concealment/blinding/protocol reporting generally incomplete; no domain-level evidence supporting High risk identified. |
| H18 | H. pylori | Some concerns | Some concerns | Low | Low | Some concerns | Some concerns | Allocation concealment/blinding/protocol reporting generally incomplete; no domain-level evidence supporting High risk identified. |
| S15 | H. pylori | Some concerns | Some concerns | Low | Low | Some concerns | Some concerns | Allocation concealment/blinding/protocol reporting generally incomplete; no domain-level evidence supporting High risk identified. |
| S21 | H. pylori | Some concerns | Some concerns | Low | Low | Some concerns | Some concerns | Allocation concealment/blinding/protocol reporting generally incomplete; no domain-level evidence supporting High risk identified. |
| S28 | H. pylori | Some concerns | Some concerns | Low | Low | Some concerns | Some concerns | Allocation concealment/blinding/protocol reporting generally incomplete; no domain-level evidence supporting High risk identified. |
| S31 | H. pylori | Some concerns | Some concerns | Low | Some concerns | Some concerns | Some concerns | Source inconsistency between C13 label and 14C procedure retained; no silent correction. |
| S33 | H. pylori | Some concerns | Some concerns | Low | Some concerns | Some concerns | Some concerns | Allocation concealment/blinding/protocol reporting generally incomplete; no domain-level evidence supporting High risk identified. |
| S37 | H. pylori | Some concerns | Some concerns | Low | Low | Some concerns | Some concerns | Allocation concealment/blinding/protocol reporting generally incomplete; no domain-level evidence supporting High risk identified. |
| S45 | H. pylori | Some concerns | Some concerns | Low | Some concerns | Some concerns | Some concerns | Allocation concealment/blinding/protocol reporting generally incomplete; no domain-level evidence supporting High risk identified. |
| S52 | H. pylori | Some concerns | Some concerns | Low | Low | Some concerns | Some concerns | Allocation concealment/blinding/protocol reporting generally incomplete; no domain-level evidence supporting High risk identified. |
| S32 | H. pylori | Some concerns | Some concerns | Low | Some concerns | Some concerns | Some concerns | Allocation concealment/blinding/protocol reporting generally incomplete; no domain-level evidence supporting High risk identified. |
| S49 | H. pylori | Some concerns | Some concerns | Some concerns | Some concerns | Some concerns | Some concerns | FAS/PPS/SS exclude post-randomisation participants and the prose/table group labels conflict (notably 42 vs 47 for the non-combined groups); outcome-specific missingness cannot be fully reconstructed. Allocation concealment and an auditable pre-specified analysis plan were not reported. |
| S50 | H. pylori | Some concerns | Some concerns | Low | Some concerns | Some concerns | Some concerns | Allocation concealment/blinding/protocol reporting generally incomplete; no domain-level evidence supporting High risk identified. |
| S42 | H. pylori | Some concerns | Some concerns | Some concerns | Some concerns | Some concerns | Some concerns | Three arms randomized 32 each; dropouts were 4, 3 and 5, leaving 28/29/27 completers. The frozen eligible comparison therefore has 57/27 completers versus 64/32 randomized; intervention blinding and an auditable pre-specified analysis plan were not reported. |
| S2 | Safety | Some concerns | Some concerns | Low | Some concerns | Some concerns | Some concerns | Adverse event symptoms included loss of appetite, nausea/vomiting, and somnolence |
| S3 | Safety | Some concerns | Some concerns | Low | Some concerns | Some concerns | Some concerns | Treatment group: Modified Buxu Yangwei decoction + conventional therapy; control group: conventional therapy; adverse event symptoms included headache, loss of appetite, somnolence, and nausea/vomiting |
| S4 | Safety | Some concerns | Some concerns | Low | Some concerns | Some concerns | Some concerns | Treatment group: Shenzhi Xiaowei decoction + Teprenone; control group: Teprenone; adverse event symptoms included constipation, abdominal pain, and dry mouth |
| S9 | Safety | Some concerns | Some concerns | Low | Some concerns | Some concerns | Some concerns | No adverse events occurred in either group during treatment |
| S10 | Safety | Some concerns | Some concerns | Low | Some concerns | Some concerns | Some concerns | Treatment group: Modified Huatan Xiaoyu formula + conventional therapy; control group: conventional therapy; adverse event symptoms included nausea, vomiting, and gastrointestinal discomfort |
| S15 | Safety | Some concerns | Some concerns | Low | Some concerns | Some concerns | Some concerns | Treatment group: Modified Sanren decoction + triple therapy; control group: triple therapy; adverse reactions included nausea, vomiting, and rash |
| S16 | Safety | Some concerns | Some concerns | Low | Some concerns | Some concerns | Some concerns | No obvious adverse reactions were observed in either group |
| S24 | Safety | Some concerns | Some concerns | Low | Some concerns | Some concerns | Some concerns | Observation group: constipation 3, nausea 1, dry mouth 2; control group: constipation 2, nausea 1, dry mouth 0. |
| S29 | Safety | Some concerns | Some concerns | Low | Some concerns | Some concerns | Some concerns | Observation group: dry mouth 1, sleep disturbance 1; control group: nausea/vomiting 1, slow response 1. Between-group difference was not statistically significant. |
| S38 | Safety | Some concerns | Some concerns | Low | Some concerns | Some concerns | Some concerns | Observation group: nausea/vomiting 2; control group: nausea/vomiting 1, rash 1, headache 1. |
| S48 | Safety | Some concerns | Some concerns | Low | Some concerns | Some concerns | Some concerns | Adverse events mainly included abdominal pain, vomiting, and constipation. |
| S49 | Safety | Some concerns | Some concerns | Some concerns | Some concerns | Some concerns | Some concerns | Composite safety outcome and FAS denominator/reporting inconsistency. |
| S51 | Safety | Some concerns | Some concerns | Low | Some concerns | Some concerns | Some concerns | Treatment group: nausea/vomiting 3, insomnia 1, abdominal pain/diarrhea 2; control group: nausea/vomiting 2, abdominal pain/diarrhea 1, dizziness/headache 1. |
| S52 | Safety | Some concerns | Some concerns | Low | Some concerns | Some concerns | Some concerns | Observation group: rash 2, diarrhea 3, dry mouth 2, abdominal pain 1, lethargy 1; control group: rash 1, diarrhea 2, dry mouth 2, abdominal pain 1, lethargy 1. |
| S14 | Safety | Some concerns | Some concerns | Some concerns | Some concerns | Some concerns | Some concerns | Randomized 50/50; 4/6 participants dropped out and only 46/44 were analysed. Reasons for dropout were not reported. Adverse-event ascertainment was clinically appropriate but participant/clinician blinding and an auditable pre-specified analysis plan were not reported. |
| S30 | Safety | Some concerns | Some concerns | Low | Some concerns | Some concerns | Some concerns | Allocation concealment/blinding/protocol reporting generally incomplete; no domain-level evidence supporting High risk identified. |
| S40 | Safety | Some concerns | Some concerns | Low | Some concerns | Some concerns | Some concerns | Allocation concealment/blinding/protocol reporting generally incomplete; no domain-level evidence supporting High risk identified. |
| S43 | Safety | Some concerns | Some concerns | Low | Some concerns | Some concerns | Some concerns | Allocation concealment/blinding/protocol reporting generally incomplete; no domain-level evidence supporting High risk identified. |
| H03 | Safety | Some concerns | Some concerns | Low | Some concerns | Some concerns | Some concerns | Allocation concealment/blinding/protocol reporting generally incomplete; no domain-level evidence supporting High risk identified. |
| H06 | Safety | Some concerns | Some concerns | Low | Some concerns | Some concerns | Some concerns | Allocation concealment/blinding/protocol reporting generally incomplete; no domain-level evidence supporting High risk identified. |
| H08 | Safety | Some concerns | Some concerns | Low | Some concerns | Some concerns | Some concerns | Allocation concealment/blinding/protocol reporting generally incomplete; no domain-level evidence supporting High risk identified. |
| H15 | Safety | Some concerns | Some concerns | Low | Some concerns | Some concerns | Some concerns | Allocation concealment/blinding/protocol reporting generally incomplete; no domain-level evidence supporting High risk identified. |
| H20 | Safety | Low | Low | Low | Low | Some concerns | Some concerns | Independent biostatistician-generated blocked randomisation, independent drug numbering/distribution, and double blinding support low risk for D1 and D2. For the eligible add-on comparison, the safety set was 84/84 for MLD-FA and 166/168 for FA; adverse events were monitored by blinded physicians. D5 remains Some concerns because the article alone does not fully verify a pre-unblinding analysis plan for every reported result. |
| H20 | H20 long-term pathology | Low | Low | Some concerns | Some concerns | Some concerns | Some concerns | Independent biostatistician-generated blocked randomisation and independent allocation/drug handling support low risk for D1. The eligible add-on comparison randomized 84/168 (MLD-FA vs FA); 12-month pathology efficacy used FAS 81/156 (PPS 72/141), with losses/discontinuations documented. Pathologist treatment-awareness and a complete pre-unblinding analysis plan were not fully verifiable from the article. |

# SR4. Primary pathology architecture and sensitivity results

Companion study-level audit: Supplementary Data 7 (Pathology Measurement, Baseline and Outcome-Selection Audit) documents pathology scale and score direction, biopsy site/protocol, source-supported baseline pathology information, selected endpoint/site/scale, the result-selection rationale, Broad/Restricted status, baseline-balanced sensitivity membership, and exclusion/restriction reasons. Contributing scales included ordinal 0–3 or 0/1/2/3 grades, 0/3/6/9 grades, and source-defined or multi-item totals; sampling ranged from explicit antrum/body/angulus or multi-site biopsy to incompletely reported sites. The final audit contains 80 principal lesion-specific effects, of which 61 meet the baseline-balanced rule and 19 do not. Final source-level quality assurance restored omitted numeric baseline fields and corrected one demonstrable membership error: S38 dysplasia/IEN had directly auditable source baseline values but had previously been flagged out. The affected dysplasia/IEN baseline-balanced sensitivity was therefore rerun from k=15 to k=16; all other primary/sensitivity memberships remained unchanged. All full-text-verification and rule-adjudication items are closed, with zero placeholder or open-adjudication statuses. NR is retained where the source did not support a value.

Frozen result-selection hierarchy: (1) end of treatment; (2) lesion-specific constructs kept separate; (3) source-declared overall lesion value; (4) source-declared primary site/scale when explicit; (5) otherwise preserve all eligible candidates and apply the frozen shared-study/shared-control construction without selecting by effect size or significance. Anatomical-site and scale criteria were used only to define the additional Restricted clinical-comparability sensitivity.

| **Outcome** | **Analysis** | **k** | **n** | **Effect (95% CI)** | **I²** | **95% PI** | **Interpretation** |
| --- | --- | --- | --- | --- | --- | --- | --- |
| Atrophy | Broad | 30 | 2937 | SMD -1.63 (-2.03 to -1.24) | 92.7% | -3.73 to 0.46 | Exploratory Broad analysis |
| Atrophy | Restricted | 4 | 356 | SMD -0.82 (-1.79 to 0.15) | high | wide; crosses 0 | Additional sensitivity; imprecise |
| Atrophy | Baseline-balanced | 23 | 2165 | SMD -1.65 (-2.12 to -1.18) | high | crosses 0 | Direction preserved |
| Atrophy | Exclude High RoB | 29 | 2851 | SMD -1.67 (-2.07 to -1.27) | high | crosses 0 | S20 excluded |
| IM | Broad | 29 | 2846 | SMD -1.48 (-1.90 to -1.06) | 90.5% | -3.61 to 0.64 | Exploratory Broad analysis |
| IM | Restricted | 3 | 272 | SMD -0.40 (-1.13 to 0.34) | moderate/high | crosses 0 | Additional sensitivity; imprecise |
| IM | Baseline-balanced | 22 | 2074 | SMD -1.37 (-1.83 to -0.92) | high | crosses 0 | Direction preserved |
| IM | Exclude High RoB | 28 | 2760 | SMD -1.48 (-1.92 to -1.05) | high | crosses 0 | S20 excluded |
| Dysplasia/IEN | Broad | 21 | 2006 | SMD -1.25 (-1.51 to -0.99) | 82.4% | -2.36 to -0.13 | PI below null |
| Dysplasia/IEN | Restricted | 3 | 272 | SMD -0.82 (-2.55 to 0.92) | 87.1% | wide | Additional sensitivity; imprecise |
| Dysplasia/IEN | Baseline-balanced | 16 | 1560 | SMD -1.13 (-1.43 to -0.83) | 82.2% | -2.25 to -0.01 | Direction preserved; PI narrowly below 0 after documented S38 source-data correction |

# SR5. Clinically interpretable pathological outcomes and H20 standalone add-on evidence

# Final source-level comparison adjudication confirmed that H20 remains eligible because the four-arm trial contains Moluodan plus folic acid versus folic acid alone, which satisfies the review's add-on PICOS. Earlier standalone calculations based on Moluodan monotherapy versus folic acid were retired. The table below uses only the eligible add-on comparison; this correction does not affect the 69-study count or the principal Broad/Restricted meta-analyses.

| **Construct** | **Studies** | **Participants** | **Effect** | **Pooling status** | **Notes** |
| --- | --- | --- | --- | --- | --- |
| Pure atrophy histological improvement | 2 | 158 | RR 1.32 (0.30 to 5.87) | Meta-analysis | S18 + U04; imprecise |
| Pure IM histological improvement | 3 | 291 | RR 1.68 (0.74 to 3.84) | Meta-analysis | S18 + S33 + U04; imprecise |
| Pure dysplasia/IEN improvement | 1 | NR | Source-reported only | Narrative | S18 only |
| Atrophy normalisation | 1 | 100 | 21/50 vs 6/50 | Narrative | Separate construct |
| IM resolution | 1 | 100 | 8/50 vs 3/50 | Narrative sensitivity | Separate resolution sensitivity construct |
| H20 global staging improvement | 1 | 237 | RR 0.85 (0.58 to 1.24) | Standalone | 12-month FAS; Moluodan + folic acid vs folic acid |
| H20 global histological scoring improvement | 1 | 237 | RR 0.95 (0.74 to 1.23) | Standalone | 12-month FAS; eligible add-on comparison |
| H20 low-grade dysplasia disappearance | 1 | 57 | RR 1.13 (0.71 to 1.81) | Standalone | 12-month FAS dysplasia subset; eligible add-on comparison |

# SR6. H. pylori and safety analysis hierarchy

Companion study-level H. pylori audit: Supplementary Data 8 records baseline-positive denominators, baseline and follow-up diagnostic methods, confirmation timing, antibiotic/bismuth and PPI/P-CAB washout information, accompanying anti-H. pylori regimens, events/denominators, and final strict test-of-cure classification. The guideline-aligned strict subset required a baseline-positive denominator; a follow-up urea breath test, fecal antigen test, or biopsy-based test; testing at least four weeks after completion of H. pylori therapy; PPI/P-CAB withheld for at least two weeks and antibiotics/bismuth for at least four weeks before testing; and source-reported or unambiguously calculable timing/washout. Unsupported assumptions were not permitted. Applying this corrected rule did not change membership: S21, S52 and H18 remain the only strict members. The evidence sets remain Broad k=15/n=1,511, defined anti-H. pylori regimen k=12/n=1,291, and strict test-of-cure k=3/n=242, with no effect-size recalculation triggered.

| **Domain** | **Analysis** | **k evidence** | **k contributing** | **n** | **Effect (95% CI)** | **Interpretation** |
| --- | --- | --- | --- | --- | --- | --- |
| H. pylori | Broad baseline-positive | 15 | 15 | 1511 | RR 1.25 (1.15 to 1.37) | Clearance/negative conversion; not labelled confirmed eradication |
| H. pylori | Defined anti-H. pylori regimen | 12 | 12 | 1291 | RR 1.22 (1.13 to 1.32) | Additional sensitivity subset |
| H. pylori | Strict test-of-cure | 3 | 3 | 242 | RR 1.19 (0.91 to 1.56) | Imprecise |
| Safety | Broad RR | 14 | 12 | 1407 | RR 0.93 (0.58 to 1.50) | Two double-zero studies retained but non-contributory to RR |
| Safety | Clinical-AE-only RR | 13 | 11 | 1314 | RR 1.10 (0.73 to 1.68) | Excludes S49 composite |
| Safety | Broad RD | 14 | 14 | 1407 | RD -0.001 (-0.028 to 0.025) | Retains double-zero studies |
| Safety | Clinical-AE-only RD | 13 | 13 | 1314 | RD 0.002 (-0.020 to 0.024) | Retains double-zero studies |

# SR7. Subgroup and meta-regression audit

Twenty of 21 specified subgroup interaction tests and eight of nine specified univariable meta-regressions were estimable under the minimum-size/variation rules. Benjamini-Hochberg adjustment was applied separately across the 20 estimable subgroup interaction tests and across the eight estimable meta-regression tests; non-estimable analyses were not included in the corresponding multiplicity family. No result remained statistically significant after adjustment. Dysplasia/IEN by H. pylori status was not formally analysed because of insufficient classifiable studies/categories.

| **Analysis** | **Outcome/covariate** | **Estimate or raw P** | **BH-adjusted P** | **Conclusion** |
| --- | --- | --- | --- | --- |
| Subgroup interaction | IM - broad conventional comparator | raw P=0.057 | 0.893 | Closest nominal subgroup signal; not significant |
| Meta-regression | IM - conventional comparator | beta -0.873 (-1.774 to 0.029); P=0.057 | 0.458 | Not significant |
| Meta-regression | Atrophy - duration / 4 weeks | beta -0.066 (-0.520 to 0.387) | 0.938 | Not significant |
| Meta-regression | IM - duration / 4 weeks | beta 0.084 (-0.378 to 0.546) | 0.938 | Not significant |
| Meta-regression | Dysplasia/IEN - duration / 4 weeks | beta -0.010 (-0.294 to 0.274) | 0.938 | Not significant |

# SR8. Small-study-effect tests

# The three principal SMD-specific Pustejovsky-Rodgers tests were independently rerun and verified in R 4.5.2 with meta version 8.2-1; sessionInfo(), inputs, logs, and outputs are archived in Supplementary Data 6.

| Outcome | Test | k tested | P value | Interpretation |
| --- | --- | --- | --- | --- |
| Atrophy | Pustejovsky-Rodgers SMD test | 30 | 0.443 | No statistical asymmetry signal; absence of a signal does not exclude dissemination bias |
| Intestinal metaplasia | Pustejovsky-Rodgers SMD test | 29 | 0.405 | No statistical asymmetry signal |
| Dysplasia/IEN | Pustejovsky-Rodgers SMD test | 21 | 0.837 | No statistical asymmetry signal |
| H. pylori clearance/negative conversion | Harbord | 15 | 0.052 | Suggestive at the P<0.10 threshold; not proof of dissemination bias |
| Reported adverse events | Harbord | 12 RR-contributing | 0.385 | No statistical asymmetry signal |

# SR9. GRADE evidence profile

| Outcome | RoB | Inconsistency | Indirectness | Imprecision | Dissemination bias | Effect | Certainty | Rationale summary |
| --- | --- | --- | --- | --- | --- | --- | --- | --- |
| Atrophy | Serious | Serious | Not serious | Not serious | Not downgraded | SMD -1.63 (-2.03 to -1.24) | Low | Serious risk of bias and serious inconsistency; PI crosses null and heterogeneity is high. Pustejovsky-Rodgers P=0.443. No additional dissemination-bias downgrade was applied; absence of asymmetry does not exclude selective dissemination. Measurement/biopsy heterogeneity was addressed under inconsistency and the Restricted sensitivity rather than double-counted as indirectness. |
| IM | Serious | Serious | Not serious | Not serious | Not downgraded | SMD -1.48 (-1.90 to -1.06) | Low | Serious risk of bias and serious inconsistency; PI crosses null and heterogeneity is high. Pustejovsky-Rodgers P=0.405. No additional dissemination-bias downgrade was applied; absence of asymmetry does not exclude selective dissemination. Measurement/biopsy heterogeneity was addressed under inconsistency and the Restricted sensitivity rather than double-counted as indirectness. |
| Dysplasia/IEN | Serious | Serious | Not serious | Not serious | Not downgraded | SMD -1.25 (-1.51 to -0.99) | Low | Serious risk of bias and serious inconsistency despite a PI below null; effect magnitudes remain heterogeneous. Pustejovsky-Rodgers P=0.837. No additional dissemination-bias downgrade was applied; absence of asymmetry does not exclude selective dissemination. Measurement/biopsy heterogeneity was addressed under inconsistency and the Restricted sensitivity rather than double-counted as indirectness. |
| H. pylori | Serious | Not serious | Not serious | Not serious | Suspected | RR 1.25 (1.15 to 1.37) | Low | Harbord P=0.052 plus small/incompletely registered evidence base; strict test-of-cure sensitivity is imprecise |
| Adverse events | Serious | Not serious | Not serious | Serious | Not downgraded | RR 0.93 (0.58 to 1.50) | Low | Serious risk of bias and serious imprecision; CI/PI are compatible with meaningful decrease or increase. Harbord P=0.385; no additional dissemination-bias downgrade was applied. |

# SR10. Published PROSPERO record and analysis-decision audit

- Published PROSPERO chronology: the original database search cutoff was 31 March 2026. The published PROSPERO record CRD420261442138 reports a review start date of 1 March 2026, first submission on 5 July 2026, and registration on 9 July 2026 (published Version 1.0). The record therefore was not prospective to the original search. At registration, formal searching/study identification was recorded as completed and screening as started, whereas data extraction, risk-of-bias assessment, and data synthesis were recorded as not started. The published record also states that no separate full protocol had been written; the published registration record is therefore the reference document for the comparison below.
- Published registration-to-final-method comparison: the core population, intervention, and randomised-study eligibility framework was retained. The intervention field already specified Chinese herbal medicine added to conventional therapy with the same conventional therapy alone as control, whereas the comparator free-text field was broader and also mentioned placebo/non-Chinese-herbal controls; the final review operationalised the stricter same-background add-on comparison. The registered pathological outcome framework was broader, including histopathological scores, pathological response/reversal measures, and OLGA/OLGIM staging; the final revision separated the three lesion-specific principal constructs from composite or stage-based outcomes and added the Restricted clinically comparable pathology sensitivity. The registered effect-measure families (RR, MD, SMD) were retained, but the statistical model was refined from a heterogeneity-dependent fixed/random approach to a consistent REML random-effects model with Hartung-Knapp inference. The registered reporting-bias plan specified funnel plots and conventional Egger testing when at least 10 studies were available; the final revision used Pustejovsky-Rodgers testing for SMD outcomes and Harbord testing for RR outcomes. Restricted pathology, baseline-balanced, High-RoB-exclusion, H. pylori regimen/test-of-cure, clinical-adverse-event-only, RD, and other robustness analyses were added during revision and are not described as prespecified.

| **Methodological item** | **Published PROSPERO record** | **Final revised review** | **Classification** |
| --- | --- | --- | --- |
| Chronology / review stage | Review start 1 Mar 2026; first submission 5 Jul 2026; registered 9 Jul 2026. Search completed; screening started; data extraction, RoB and synthesis not started. | Original search ended 31 Mar 2026; final update completed 16 Aug 2026; full review completed. | Registration not prospective to original search; chronology reported transparently. |
| Full protocol | No separate full protocol had been written. | Published PROSPERO record used as registration reference. | Clarified. |
| Population / study design | Gastric precancerous lesions including CAG, IM and dysplasia/IEN; RCTs only. | Same core population and RCT requirement. | Retained / clarified. |
| Intervention | Chinese herbal medicine + conventional Western medicine; intervention field states same conventional therapy alone as control. | Chinese herbal medicine added to the same conventional therapy. | Core add-on principle retained. |
| Comparator | Comparator free-text field additionally mentioned placebo + conventional therapy or other non-Chinese-herbal controls. | Eligibility operationalised to the stricter same-background conventional-therapy comparator. | Narrowed / operationalised. |
| Pathological outcomes | Broad pathological improvement: scores, response/reversal, OLGA/OLGIM when reported. | Three lesion-specific principal score outcomes; composite/stage evidence separated; Restricted sensitivity added. | Architecture refined during revision. |
| Synthesis model | Fixed-effect if heterogeneity low; otherwise random-effects considered. | Consistent REML random-effects with Hartung-Knapp inference. | Methodological refinement. |
| Small-study / reporting bias | Funnel plots + conventional Egger test when k≥10. | Pustejovsky-Rodgers for SMD; Harbord for RR. | Methodological refinement. |
| Additional robustness analyses | Not specified in the published record. | Restricted pathology, baseline-balanced, High-RoB-exclusion, H. pylori strict TOC, clinical-AE-only, RD and related sensitivities. | Revision-driven; not prespecified. |

- Timing of revision-stage analytical decisions: the end-of-treatment preference was present in the submitted Methods. The explicit site/scale tie-breakers, Restricted clinical-comparability criteria, baseline-balanced rule, and additional robustness analyses were finalised during revision before the corresponding pooled effects were re-estimated; no result was selected because it was statistically favourable. Final source-level quality assurance subsequently identified one demonstrable data-flag error (S38 dysplasia/IEN baseline membership); correcting that source-supported error required rerunning only the affected baseline-balanced sensitivity and did not reopen the underlying analytical rule.
- Search strategies: complete original strategies (inception to 31 March 2026) and the 2026 update strategies/audit (through 16 August 2026) are supplied as Supplementary Appendix 1. Platform-specific syntax adaptations are documented without changing the underlying search concepts.
- Historical 26 retrieval reconstruction: completed. Exact identities were reconstructed. Title- and author-based searches were repeated in CNKI, Wanfang Data, and VIP. Because the 26 full texts were still not directly accessible, original-article requests were submitted through Wanfang Library document delivery, through which 15 reports were obtained and assessed. For the remaining 11 reports, the relevant journal official websites and historical journal archives were searched further; the full texts remained unavailable. See Supplementary Table SR13.
- Included-study bibliography: synchronized to the final 69-study evidence base in the manuscript; study identity follows the Reference Master rather than archive filenames. U02 and U04 bibliographic metadata are closed in the final reference audit.
- Non-principal secondary outcomes: final integration completed. Statistical results, multiplicity checks, sensitivities, method-corrected small-study-test status, and GRADE profiles are provided in Supplementary Tables S2, S5, S6, and S7 below.
- Final figures: Figure 1 (study-selection/PRISMA eligibility audit), Figure 2 (RoB 2 distribution for primary continuous pathology), and Figure 3 (exploratory Broad estimates for the three principal pathological outcomes with confidence and prediction intervals) have been regenerated or verified against the final audit. The Figure 1 retrieval note now consistently reports 15 later retrieved/assessed and 11 remaining unavailable after additional retrieval attempts, with no pending report. These display updates do not reopen eligibility or the unchanged Broad analyses.

# S2. Final 69-study non-principal secondary-outcome meta-analysis results

These results supersede the historical 52-study secondary-outcome estimates. H. pylori and safety are retained in SR6/SR9 as principal outcomes and are not duplicated as secondary GRADE rows here.

| **Outcome** | **Measure** | **k** | **n** | **Effect (95% CI)** | **I²** | **95% PI** | **Small-study test** |
| --- | --- | --- | --- | --- | --- | --- | --- |
| Clinical total effective rate | RR | 33 | 3,287 | 1.23 (1.19 to 1.27) | 0.0% | 1.19 to 1.27 | Harbord P=0.424; no signal |
| Clinical symptom response | RR | 8 | 905 | 1.21 (1.13 to 1.30) | 0.0% | 1.13 to 1.30 | Not tested (k<10) |
| TCM symptom/syndrome effective rate | RR | 11 | 1,024 | 1.22 (1.15 to 1.30) | 0.0% | 1.15 to 1.30 | Harbord P=0.102; no signal |
| Total TCM symptom/syndrome score | SMD | 11 | 978 | -1.54 (-2.34 to -0.73) | 93.7% | -4.28 to 1.21 | Pustejovsky-Rodgers not re-estimated; not assessable from archived secondary inputs |
| Pure endoscopic response | RR | 5 | 693 | 1.36 (1.10 to 1.68) | 50.6% | 0.88 to 2.10 | Not tested (k<10) |
| Endoscopy + pathology joint response | RR | 3 | 302 | 1.53 (1.07 to 2.19) | 0.0% | 0.54 to 4.38 | Not tested (k<10) |
| PGI | SMD | 16 | 1,634 | 2.50 (1.60 to 3.41) | 94.8% | -1.08 to 6.08 | Pustejovsky-Rodgers not re-estimated; not assessable from archived secondary inputs |
| PGII | SMD | 13 | 1,312 | 0.42 (-0.30 to 1.15) | 95.6% | -2.25 to 3.10 | Pustejovsky-Rodgers not re-estimated; not assessable from archived secondary inputs |
| PGR | MD | 7 | 784 | 2.01 (1.15 to 2.86) | 90.3% | -0.28 to 4.29 | Not tested (k<10) |
| CRP | MD | 3 | 358 | -1.55 (-13.92 to 10.82) | 99.0% | -73.67 to 70.58 | Not tested (k<10); H11 source conflict flagged |
| IL-1beta | MD | 2 | 190 | -8.84 (-69.01 to 51.33) | 94.6% | Not estimable | Not tested (k<10) |
| IL-6 | MD | 4 | 396 | -7.56 (-13.84 to -1.28) | 96.3% | -26.15 to 11.02 | Not tested (k<10) |
| IL-8 | MD | 3 | 284 | -8.01 (-24.85 to 8.83) | 99.1% | -106.70 to 90.69 | Not tested (k<10) |
| TNF-alpha | SMD | 5 | 524 | -1.59 (-2.96 to -0.22) | 94.6% | -5.34 to 2.16 | Not tested (k<10) |
| Poor appetite | SMD | 13 | 1,373 | -1.20 (-1.55 to -0.84) | 87.8% | -2.45 to 0.05 | Pustejovsky-Rodgers not re-estimated; not assessable from archived secondary inputs |
| Fatigue/lassitude | SMD | 8 | 919 | -1.09 (-1.55 to -0.64) | 80.0% | -2.36 to 0.17 | Not tested (k<10) |
| Epigastric/gastric pain | SMD | 14 | 1,409 | -2.13 (-2.92 to -1.34) | 95.7% | -5.15 to 0.88 | Pustejovsky-Rodgers not re-estimated; not assessable from archived secondary inputs |
| Fullness/distension | SMD | 15 | 1,589 | -1.47 (-2.05 to -0.89) | 91.8% | -3.69 to 0.74 | Pustejovsky-Rodgers not re-estimated; not assessable from archived secondary inputs |
| Loose stool | SMD | 5 | 576 | -1.98 (-3.58 to -0.37) | 95.8% | -6.38 to 2.43 | Not tested (k<10) |
| Acid reflux/regurgitation | SMD | 5 | 440 | -1.40 (-2.91 to 0.11) | 94.4% | -5.53 to 2.72 | Not tested (k<10) |
| Dry mouth/bitter taste | SMD | 4 | 490 | -2.33 (-3.60 to -1.06) | 87.4% | Not estimable | Not tested (k<10) |
| Belching | SMD | 3 | 264 | -1.15 (-3.60 to 1.29) | 93.7% | Not estimable | Not tested (k<10) |
| Unsatisfactory stool | SMD | 3 | 433 | -0.88 (-1.82 to 0.05) | 76.8% | Not estimable | Not tested (k<10) |

# S5. Final 69-study secondary-outcome sensitivity analyses

Sensitivity memberships were frozen before result inspection. Analyses rendered invalid by the final eligibility reassessment are explicitly retired rather than silently retained.

| **Sensitivity** | **Status** | **k / n** | **Result** | **Interpretation** |
| --- | --- | --- | --- | --- |
| Clinical total, exclude H03 source inconsistency | Final | 32 | RR 1.23 (1.19 to 1.28) | Conclusion unchanged |
| Total TCM score, restricted explicitly labelled set | Final | 6 / 571 | SMD -1.30 (-1.99 to -0.60); I²=87.0%; PI -3.17 to 0.57 | Members S5, S6, S17, S49b, H19, U04 |
| Total TCM score, broader construct incl. S42 | Final | 12 / 1,062 | SMD -1.43 (-2.19 to -0.66); I²=93.7%; PI -4.13 to 1.27 | Broader-construct sensitivity |
| PGI, exclude S8 | Final | 15 / 1,564 | SMD 2.21 (1.53 to 2.89); I²=93.6%; PI -0.43 to 4.84 | Direction unchanged |
| PGR, leave-one-out | Final | 7 / 784 | All seven leave-one-out 95% CIs >0; pooled MD range 1.79 to 2.18 | No single study reversed direction |
| IL-6, add S24 using SMD | Final | 5 / 496 | SMD -2.41 (-3.87 to -0.95); I²=95.9%; PI -6.41 to 1.59 | Scale-harmonisation sensitivity |
| TNF-alpha, add S24 using SMD | Final | 6 / 624 | SMD -1.92 (-3.26 to -0.58); I²=95.8%; PI -5.67 to 1.84 | Scale-harmonisation sensitivity |
| Old same 0-21 TCM scale S40/S41 | Retired invalid | — | — | S41 is excluded from the final 69-study evidence base |
| Old PGII sensitivity including S23 | Retired invalid | — | — | S23 is excluded from the final 69-study evidence base |

# S6. Final 69-study small-study-effect and multiplicity checks

Formal small-study testing required k>=10 statistically independent contributing comparisons. RR outcomes used the Harbord modified score test. For SMD outcomes, the Pustejovsky-Rodgers test is the method-appropriate asymmetry test because conventional Egger regression can be biased by the mathematical association between an SMD and its standard error. Complete study-level asymmetry inputs were retained for the three principal pathology SMD outcomes and were re-analysed accordingly (SR8). For non-principal secondary SMD outcomes, those study-level asymmetry inputs were not preserved in the archive; therefore, their legacy conventional Egger P values are retired and no replacement formal P value is claimed. Two-sided P<0.10 is interpreted as suggestive only. Contour-enhanced funnel plots for the five principal outcomes are provided in Supplementary Data 5.

| Outcome | Method | k | P | Suggestive P<0.10? |
| --- | --- | --- | --- | --- |
| Clinical total effective rate | Harbord | 33 | 0.424 | No |
| TCM symptom/syndrome effective rate | Harbord | 11 | 0.102 | No |
| Total TCM symptom/syndrome score | Pustejovsky-Rodgers not re-estimated | 11 | NA | Not assessable |
| PGI | Pustejovsky-Rodgers not re-estimated | 16 | NA | Not assessable |
| PGII | Pustejovsky-Rodgers not re-estimated | 13 | NA | Not assessable |
| Poor appetite | Pustejovsky-Rodgers not re-estimated | 13 | NA | Not assessable |
| Epigastric/gastric pain | Pustejovsky-Rodgers not re-estimated | 14 | NA | Not assessable |
| Fullness/distension | Pustejovsky-Rodgers not re-estimated | 15 | NA | Not assessable |

## Benjamini-Hochberg adjustment across the nine individual symptom outcomes:

| **Symptom** | **BH-adjusted P** | **BH<0.05?** |
| --- | --- | --- |
| Poor appetite | 0.000079 | Yes |
| Fatigue/lassitude | 0.001732 | Yes |
| Epigastric/gastric pain | 0.000270 | Yes |
| Fullness/distension | 0.000271 | Yes |
| Loose stool | 0.040040 | Yes |
| Acid reflux/regurgitation | 0.068852 | No |
| Dry mouth/bitter taste | 0.017947 | Yes |
| Belching | 0.179718 | No |
| Unsatisfactory stool | 0.068852 | No |

# S7. Final 69-study GRADE profile for 23 non-principal secondary outcomes

The principal GRADE ratings for atrophy, intestinal metaplasia, dysplasia/IEN, H. pylori, and safety are reported in SR9. The table below is restricted to the 23 non-principal secondary outcomes. After method correction, nine are Low certainty and fourteen are Very low certainty; no non-principal secondary outcome is Moderate or High certainty.

| **Outcome** | **k / n** | **Effect (95% CI)** | **Heterogeneity / PI** | **Certainty** | **Key reason** |
| --- | --- | --- | --- | --- | --- |
| Clinical total effective rate | 33 / 3,287 | RR 1.23 (1.19 to 1.27) | I² 0.0%; PI 1.19 to 1.27 | Low | Serious risk of bias and serious indirectness from non-standard composite/subjective outcome definitions. |
| Clinical symptom response | 8 / 905 | RR 1.21 (1.13 to 1.30) | I² 0.0%; PI 1.13 to 1.30 | Low | Serious risk of bias and serious indirectness from subjective, generally unblinded response definitions. |
| TCM symptom/syndrome effective rate | 11 / 1,024 | RR 1.22 (1.15 to 1.30) | I² 0.0%; PI 1.15 to 1.30 | Low | Serious risk of bias and serious indirectness from heterogeneous TCM response definitions. |
| Total TCM symptom/syndrome score | 11 / 978 | SMD -1.54 (-2.34 to -0.73) | I² 93.7%; PI -4.28 to 1.21 | Low | Serious risk of bias and serious inconsistency; the legacy conventional Egger result is retired and corrected SMD asymmetry testing is not assessable from archived secondary inputs. |
| Pure endoscopic response | 5 / 693 | RR 1.36 (1.10 to 1.68) | I² 50.6%; PI 0.88 to 2.10 | Very low | Serious risk of bias, inconsistency and indirectness; the prediction interval crosses the null. |
| Endoscopy + pathology joint response | 3 / 302 | RR 1.53 (1.07 to 2.19) | I² 0.0%; PI 0.54 to 4.38 | Very low | Serious risk of bias, indirectness and imprecision for a non-standard joint endoscopy-pathology response. |
| PGI | 16 / 1,634 | SMD 2.50 (1.60 to 3.41) | I² 94.8%; PI -1.08 to 6.08 | Very low | Serious risk of bias, inconsistency and indirectness; corrected SMD asymmetry testing is not assessable from archived secondary inputs. |
| PGII | 13 / 1,312 | SMD 0.42 (-0.30 to 1.15) | I² 95.6%; PI -2.25 to 3.10 | Very low | Serious risk of bias, very serious inconsistency, indirectness and imprecision; corrected SMD asymmetry testing is not assessable. |
| PGR | 7 / 784 | MD 2.01 (1.15 to 2.86) | I² 90.3%; PI -0.28 to 4.29 | Very low | Serious risk of bias, inconsistency and indirectness for a surrogate biomarker. |
| CRP | 3 / 358 | MD -1.55 (-13.92 to 10.82) | I² 99.0%; PI -73.67 to 70.58 | Very low | Serious risk of bias, very serious inconsistency, indirectness and imprecision; H11 has a documented source-direction conflict. |
| IL-1beta | 2 / 190 | MD -8.84 (-69.01 to 51.33) | I² 94.6%; PI Not estimable | Very low | Serious risk of bias and indirectness, with serious inconsistency and very serious imprecision based on only two studies. |
| IL-6 | 4 / 396 | MD -7.56 (-13.84 to -1.28) | I² 96.3%; PI -26.15 to 11.02 | Very low | Serious risk of bias and indirectness with very serious inconsistency; the prediction interval crosses the null widely. |
| IL-8 | 3 / 284 | MD -8.01 (-24.85 to 8.83) | I² 99.1%; PI -106.70 to 90.69 | Very low | Serious risk of bias and indirectness with very serious inconsistency and serious imprecision. |
| TNF-alpha | 5 / 524 | SMD -1.59 (-2.96 to -0.22) | I² 94.6%; PI -5.34 to 2.16 | Very low | Serious risk of bias and indirectness with very serious inconsistency; the prediction interval crosses the null widely. |
| Poor appetite | 13 / 1,373 | SMD -1.20 (-1.55 to -0.84) | I² 87.8%; PI -2.45 to 0.05 | Low | Serious risk of bias and inconsistency for a subjective symptom; corrected SMD asymmetry testing is not assessable from archived secondary inputs. |
| Fatigue/lassitude | 8 / 919 | SMD -1.09 (-1.55 to -0.64) | I² 80.0%; PI -2.36 to 0.17 | Low | Serious risk of bias and inconsistency for a subjective symptom; formal asymmetry testing was not used because k<10. |
| Epigastric/gastric pain | 14 / 1,409 | SMD -2.13 (-2.92 to -1.34) | I² 95.7%; PI -5.15 to 0.88 | Low | Serious risk of bias and inconsistency; the legacy conventional Egger signal is retired and corrected SMD asymmetry testing is not assessable from archived secondary inputs. |
| Fullness/distension | 15 / 1,589 | SMD -1.47 (-2.05 to -0.89) | I² 91.8%; PI -3.69 to 0.74 | Low | Serious risk of bias and inconsistency; the legacy conventional Egger signal is retired and corrected SMD asymmetry testing is not assessable from archived secondary inputs. |
| Loose stool | 5 / 576 | SMD -1.98 (-3.58 to -0.37) | I² 95.8%; PI -6.38 to 2.43 | Very low | Serious risk of bias with very serious inconsistency; the prediction interval is very wide. |
| Acid reflux/regurgitation | 5 / 440 | SMD -1.40 (-2.91 to 0.11) | I² 94.4%; PI -5.53 to 2.72 | Very low | Serious risk of bias, very serious inconsistency and serious imprecision; confidence and prediction intervals cross the null. |
| Dry mouth/bitter taste | 4 / 490 | SMD -2.33 (-3.60 to -1.06) | I² 87.4%; PI Not estimable | Low | Serious risk of bias and inconsistency; only four studies contributed and formal asymmetry testing was not appropriate. |
| Belching | 3 / 264 | SMD -1.15 (-3.60 to 1.29) | I² 93.7%; PI Not estimable | Very low | Serious risk of bias, very serious inconsistency and serious imprecision based on only three studies. |
| Unsatisfactory stool | 3 / 433 | SMD -0.88 (-1.82 to 0.05) | I² 76.8%; PI Not estimable | Very low | Serious risk of bias, inconsistency and imprecision based on only three studies. |

# SR13. Historical 26 reports recorded as not retrieved: reconstructed citation list and current status

The historical 26 are retained as the original screening-stage not-retrieved set. During the retrieval reassessment, title-, author-, and bibliographic-information searches were repeated in CNKI, Wanfang Data, and VIP. Because all 26 full texts remained not directly accessible, the original articles were requested through Wanfang Library document delivery. Fifteen reports were obtained through this route and reassessed using the unchanged PICOS criteria. For the remaining eleven reports, the relevant journal official websites and historical journal archives were searched further, but the full texts could not be obtained. Their continued absence could theoretically contribute to retrieval bias, so they were not assumed to be ineligible without full-text verification. None changes the final eligibility set.

| **Historical PRISMA no.** | **Citation** | **Current status** |
| --- | --- | --- |
| 2 | 边海溪. 中西医结合治疗慢性萎缩性胃炎42例. 2010. 中外健康文摘 7(15):290-1. 10.3969/j.issn.1672-5085.2010.15.282 | Later retrieved and assessed |
| 23 | 陈生良, 陈志原. 30例慢性萎缩性胃炎患者中西医结合治疗法治疗临床分析. 2015. 大家健康（下旬版） 2:318. 10.3969/j.issn.1009-6019.2015.02.412 | Later retrieved and assessed |
| 25 | 陈松富. 益气养阴汤剂治疗慢性萎缩性胃炎疗效分析. 2012. 按摩与康复医学 3(21). | Later retrieved and assessed |
| 27 | 陈曦光, 孙瑞. 白拟胃炎冲剂抗幽门螺杆菌感染37例临床观察. 2009. 国际中医中药杂志 31(3). | Later retrieved and assessed |
| 32 | 陈志. 中西医结合治疗慢性萎缩性胃炎的临床疗效分析. 2015. 中国实用乡村医生杂志 23. | Later retrieved and assessed |
| 34 | 单茜, 陈晓晖. 慢性萎缩性胃炎54例中医辨证体会. 2014. 中国保健营养（下旬刊） 6:3072-3. | Later retrieved and assessed |
| 40 | 邓照华. 益气健脾汤治疗慢性萎缩性胃炎33例疗效观察. 2006. 丹东医药 4. | Later retrieved and assessed |
| 62 | 冯乐琴. 慢性萎缩性胃炎的中西医结合方法治疗探究. 2020. 特别健康 29:41-2. | Still unavailable after additional retrieval attempts |
| 84 | 葛梅凌. 中西医结合治疗60例慢性萎缩性胃炎疗效观察. 2012. 婚育与健康·实用诊疗 10:63-4. | Still unavailable after additional retrieval attempts |
| 95 | 韩婷, 李燕, 马莉. 慢性萎缩性胃炎的中西医结合治疗效果. 2016. 世界最新医学信息文摘 16(69):265. | Later retrieved and assessed |
| 106 | 胡景春, 唐婷. 在Hp阳性慢性萎缩性胃炎中采用加味半夏泻心汤的治疗效果与应用价值分析. 2019. 特别健康 24:36,39. | Still unavailable after additional retrieval attempts |
| 109 | 胡新云. 观察中西药物联用治疗慢性萎缩性胃炎的临床疗效. 2014. 中外健康文摘 22:157-9. | Later retrieved and assessed |
| 126 | 江湘柳. 中西医结合治疗萎缩性胃炎80例临床与病理观察. 2015. 延边医学 4:168-9. | Later retrieved and assessed |
| 136 | 孔振. 观察益气活血养阴类中药配伍组方治疗慢性萎缩胃炎的临床疗效. 2013. 健康之路 12:477. | Later retrieved and assessed |
| 140 | 赖长春. 补益化瘀治疗慢性萎缩性胃炎疗效观察. 2007. 中国当代医学 6(13). | Still unavailable after additional retrieval attempts |
| 153 | 李国霞. 失笑散治疗慢性萎缩性胃炎. 2013. 中国农村卫生 04Z. | Still unavailable after additional retrieval attempts |
| 164 | 李明会. 中西医结合治疗慢性萎缩性胃炎. 2003. 中华综合临床医学杂志 5(9):79-80. | Still unavailable after additional retrieval attempts |
| 168 | 李少华. 中西医结合治疗慢性萎缩性胃炎60例疗效观察. 2009. 中华实用中西医杂志 22(9). | Still unavailable after additional retrieval attempts |
| 174 | 李蔚. 中西医结合治疗慢性萎缩性胃炎60例的临床观察. 2002. 中华综合临床医学杂志 4(6):41-2. | Still unavailable after additional retrieval attempts |
| 183 | 梁丽文, 梁华超, 陈文英. 中西医结合治疗慢性萎缩性胃炎的临床研究. 2021. 特别健康 25:130. | Still unavailable after additional retrieval attempts |
| 243 | 马得元. 中西医结合治疗慢性萎缩性胃炎的临床观察. 2011. 中外健康文摘 8(34):283. | Later retrieved and assessed |
| 342 | 魏六栓, 左丽萍. 自拟健胃汤治疗慢性萎缩性胃炎临床疗效观察. 2015. 医学与社会 28(B06). | Still unavailable after additional retrieval attempts |
| 370 | 徐梅. 中西医结合治疗慢性胃炎疗效观察. 2009. 医学信息（下旬刊） 1(9):158. | Later retrieved and assessed |
| 444 | 岳维建. 中西医结合治疗幽门螺杆菌感染性慢性萎缩性胃炎58例. 2003. 中国基层医药 10(11). | Later retrieved and assessed |
| 448 | 张浩. 中西医结合治疗慢性萎缩性胃炎45例的临床疗效评价. 2010. 按摩与康复医学（下旬刊） 1(8):81. | Still unavailable after additional retrieval attempts |
| 453 | 张加旺, 何斌. 抗菌三联合活血化瘀法治疗慢性萎缩性胃炎. 1999. 中国中西医结合脾胃杂志 7(2). | Later retrieved and assessed |

# SR14. Full-text exclusion audit and update-search audit

A separate supplementary spreadsheet, Supplementary Data 1 (Full-text Eligibility Audit), provides the report-level eligibility audit, one primary exclusion reason per excluded report, PICOS fields, and the formal 2026 update-search screening table. Supplementary Appendix 1 provides the complete original and 2026 update search strategies. The official final PRISMA 2020 flow reports the current integrated retrieval status, while SR13 separately preserves the historical 26-report not-retrieved snapshot and documents the later 15 retrieved/assessed and 11 still-unavailable status after the additional journal-website and historical-archive searches.

# SR15. Impact of evidence-base reassessment on the five principal outcomes

This table compares the archived historical 52-study principal results with the current 69-study results. It is a documentary before/after comparison only. Pooled effects were not rerun for this table; the current certainty column incorporates the method-corrected SMD small-study-effect assessment. The average direction of the five principal outcomes was not reversed, but study membership, heterogeneity, prediction intervals, and some certainty judgements changed.

| Outcome | Historical 52-study | Current 69-study | Historical heterogeneity / PI / GRADE | Current heterogeneity / PI / GRADE | Interpretation of evidence-base change |
| --- | --- | --- | --- | --- | --- |
| Gastric mucosal atrophy | k=36; n=3,391; SMD -1.53 (95% CI -1.88 to -1.18) | k=30; n=2,937; SMD -1.63 (95% CI -2.03 to -1.24) | I²=92.6%; PI -3.58 to 0.52; Very low | I²=92.7%; PI -3.73 to 0.46; Low | Average effect slightly larger; heterogeneity and PI interpretation are essentially unchanged. Method-appropriate SMD asymmetry testing removed the statistical dissemination-bias downgrade, so current certainty is Low. |
| Intestinal metaplasia | k=32; n=3,087; SMD -1.46 (95% CI -1.89 to -1.03) | k=29; n=2,846; SMD -1.48 (95% CI -1.90 to -1.06) | I²=92.6%; PI -3.80 to 0.89; Very low | I²=90.5%; PI -3.61 to 0.64; Low | Average estimate is nearly unchanged; heterogeneity remains high and the PI still crosses the null. Method-appropriate SMD asymmetry testing removed the statistical dissemination-bias downgrade, so current certainty is Low. |
| Dysplasia/IEN | k=25; n=2,301; SMD -1.35 (95% CI -1.67 to -1.03) | k=21; n=2,006; SMD -1.25 (95% CI -1.51 to -0.99) | I²=89.3%; PI -2.88 to 0.19; Very low | I²=82.4%; PI -2.36 to -0.13; Low | Average effect attenuated modestly and heterogeneity decreased; the current PI remains below the null. Serious inconsistency persists, but method-appropriate SMD asymmetry testing removed the statistical dissemination-bias downgrade, so current certainty is Low. |
| H. pylori clearance/negative conversion | k=9; n=982; RR 1.18 (95% CI 1.11 to 1.25) | k=15; n=1,511; RR 1.25 (95% CI 1.15 to 1.37) | I²=0%; PI 1.10 to 1.26; Moderate | I²=48.0%; PI 1.01 to 1.56; Low | Evidence set expanded and RR increased modestly, while heterogeneity appeared; source-level definition audit led to a more cautious Low-certainty rating. |
| Reported adverse events | k=15; n=1,466; RR 1.06 (95% CI 0.72 to 1.55) | Evidence set k=14; n=1,407; RR-contributing k=12; RR 0.93 (95% CI 0.58 to 1.50) | I²=11.4%; PI 0.49 to 2.27; Low | I²=34.6%; PI 0.31 to 2.85; Low | Point estimate moved from above to below 1, but both confidence intervals cross 1; the conclusion remains inconclusive and does not establish safety equivalence. |

# SR16. Original Chinese titles and provenance of English reference titles

To improve accessibility for international readers, Chinese-language references are presented in English in the main manuscript reference list. This table preserves the original Chinese article titles to support source verification and retrieval in Chinese bibliographic databases, and documents the provenance of the English title used in the main reference list. English titles printed in the supplied source articles were used preferentially. When no English title was available in the source article, official publisher or journal metadata were checked when readily identifiable. If no published English title could be verified, an author-prepared English translation is provided in square brackets in this supplementary table. The original Chinese title is retained here as the source-language record.

| **Ref.** | **Original Chinese title** | **English title** | **English-title source** |
| --- | --- | --- | --- |
| 22 | 半夏泻心汤加减联合常规西药治疗慢性萎缩性胃炎（肝胃不和证）临床观察 | [Clinical observation of modified Banxia Xiexin Decoction combined with conventional Western medicine for chronic atrophic gastritis (liver–stomach disharmony syndrome)] | Author-prepared translation |
| 23 | 辨证分型中药治疗慢性萎缩性胃炎伴肠上皮化生的临床疗效 | [Clinical efficacy of syndrome-differentiated Chinese herbal medicine for chronic atrophic gastritis with intestinal metaplasia] | Author-prepared translation |
| 24 | 补虚养胃汤加减治疗慢性萎缩性胃炎疗效及对患者胃肠功能的影响 | [Efficacy of modified Buxu Yangwei Decoction for chronic atrophic gastritis and its effects on gastrointestinal function] | Author-prepared translation |
| 25 | 参枳消萎汤治疗慢性萎缩性胃炎癌前病变疗效及对三叶因子3、胃泌素-17的影响 | [Efficacy of Shenzhi Xiaowei Decoction for precancerous lesions of chronic atrophic gastritis and its effects on trefoil factor 3 and gastrin-17] | Author-prepared translation |
| 26 | 柴胡加龙骨牡蛎汤加味治疗慢性萎缩性胃炎临床研究 | Clinical Study on Supplemented Bupleurum Decoction Plus Dragon Bones and Oyster Shell in the Treatment of Chronic Atrophic Gastritis | Original article |
| 27 | 柴朴汤联合化痰消瘀方治疗胃癌前病变伴焦虑抑郁 | Chaipu Decoction Combined with Huatan Xiaoyu Decoction in Treatment of Precancerous Lesions of Gastric Cancer with Anxiety and Depression | Original article |
| 28 | 复萎抗异汤对胃癌前病变患者胃黏膜P16、bcl-2、COX-2、survivin蛋白表达影响的研究 | [Effects of Fuwei Kangyi Decoction on gastric mucosal expression of P16, Bcl-2, COX-2, and survivin in patients with gastric precancerous lesions] | Author-prepared translation |
| 29 | 复胃消萎汤治疗脾虚湿热型慢性萎缩性胃炎伴异型增生临床观察 | Clinical observation of Fuwei Xiaowei decoction on chronic atrophic gastritis with dysplasia and syndrome of spleen deficiency and damp-heat | Original article |
| 30 | 化痰消瘀方加减治疗萎缩性胃炎伴胆汁反流疗效观察 | [Efficacy of modified Huatan Xiaoyu Formula for atrophic gastritis with bile reflux] | Author-prepared translation |
| 31 | 化痰消瘀方治疗慢性萎缩性胃炎伴糜烂的疗效分析 | Clinical effect of Huatan Xiaoyu decoction on chronic atrophic gastritis with erosion | Original article |
| 32 | 黄芪建中汤合良附丸对慢性萎缩性胃炎患者胃黏膜血流量及血清氧化应激指标的影响 | [Effects of Huangqi Jianzhong Decoction combined with Liangfu Pill on gastric mucosal blood flow and serum oxidative-stress indicators in patients with chronic atrophic gastritis] | Author-prepared translation |
| 33 | 活血化瘀法治疗慢性萎缩性胃炎的疗效及对血液流变学的影响 | [Efficacy of blood-activating and stasis-resolving therapy for chronic atrophic gastritis and its effects on haemorrheology] | Author-prepared translation |
| 34 | 加味附子理中汤治疗脾胃虚寒型慢性萎缩性胃炎临床研究 | Effects of Modified Fuzi Lizhong Decoction on Chronic Atrophic Gastritis of Deficient Cold in Spleen and Stomach | Original article |
| 35 | 加味三仁汤联合三联疗法治疗幽门螺杆菌相关慢性萎缩性胃炎的临床研究 | Clinical study on modified Sanren Decoction combined with triple therapy in treating Helicobacter pylori-related chronic atrophic gastritis | Original article |
| 36 | 健脾化痰方治疗慢性萎缩性胃炎临床观察 | [Clinical observation of Jianpi Huatan Formula for chronic atrophic gastritis] | Author-prepared translation |
| 37 | 健脾化痰中药干预治疗慢性萎缩性胃炎的效果及对HSP70表达水平的影响 | Effect of Jianpi Huatan traditional Chinese medicine intervention in the treatment of chronic atrophic gastritis and its influence on HSP70 expression level | Original article |
| 38 | 健脾活血方治疗慢性萎缩性胃炎气虚血瘀型30例 | [Jianpi Huoxue Formula for 30 cases of chronic atrophic gastritis with qi deficiency and blood stasis] | Author-prepared translation |
| 39 | 健脾活血消积方联合西药治疗慢性萎缩性胃炎疗效及作用机制探讨 | [Efficacy and potential mechanisms of Jianpi Huoxue Xiaoji Formula combined with Western medicine for chronic atrophic gastritis] | Author-prepared translation |
| 40 | 健脾理气化瘀方治疗脾虚气滞型慢性萎缩性胃炎伴糜烂临床效果 | [Clinical effects of Jianpi Liqi Huayu Formula for chronic atrophic gastritis with erosion and spleen-deficiency qi-stagnation syndrome] | Author-prepared translation |
| 41 | 健脾通络方联合四联疗法治疗HP阳性慢性萎缩性胃炎疗效及对CXCL5、Runx3的影响 | The therapeutic effect of Jianpi Tongluo prescription combined with quadruple therapy on HP positive chronic atrophic gastritis and its impact on CXCL5 and Runx3 | Official journal metadata |
| 42 | 健脾益气方治疗慢性萎缩性胃炎临床疗效 | Effect of Jianpi Yiqi Decoction on chronic atrophic gastritis | Original article |
| 43 | 理气化瘀健胃汤联合四联疗法治疗Hp阳性慢性萎缩性胃炎的疗效 | [Efficacy of Liqi Huayu Jianwei Decoction combined with quadruple therapy for H. pylori-positive chronic atrophic gastritis] | Author-prepared translation |
| 44 | 六味安消联合西药治疗萎缩性慢性胃炎伴原发性胆汁反流的临床研究 | Clinical research of chronic atrophic gastritis with primary bile regurgitation treated with liuwei anxiao capsules and western medicine | Original article |
| 45 | 慢萎散治疗胃阴不足证慢性萎缩性胃炎的临床分析 | Clinical Analysis of Manwei San in Treating Stomach Yin Deficiency Syndrome of Chronic Atrophic Gastritis | Original article |
| 46 | 慢性萎缩性胃炎合并Hp感染的中西医结合治疗效果分析 | Analysis of curative effect of integrated traditional Chinese medicine and western medicine on chronic atrophic gastritis complicated with Hp infection | Original article |
| 47 | 脾胃培源方联合叶酸片治疗慢性萎缩性胃炎脾胃虚弱证临床研究 | Clinical Study on Piwei Peiyuan Decoction Combined with Folic Acid Tablets in the Treatment of Chronic Atrophic Gastritis with Spleen-stomach Deficiency | Original article |
| 48 | 疏肝和胃汤治疗老年慢性萎缩性胃炎临床研究 | Clinical Study on Shugan Hewei Tang in the Treatment of Senile Chronic Atrophic Gastritis | Original article |
| 49 | 胃复春治疗慢性萎缩性胃炎临床研究 | Clinical Study on Weifuchun in Treating for Chronic Atrophic Gastritis | Original article |
| 50 | 胃舒煎剂结合西药治疗慢性萎缩性胃炎肠腺化生的临床研究 | Clinical study on combined “Weishu Decoction” and western medicine in treating intestinal metaplasia of chronic atrophic gastritis | Original article |
| 51 | 胃特安片合西药治疗萎缩性胃炎肠上皮化生93例 | [Weite’an Tablets combined with Western medicine for 93 cases of atrophic gastritis with intestinal metaplasia] | Author-prepared translation |
| 52 | 温阳健脾膏治疗脾胃虚弱型慢性萎缩性胃炎的临床效果 | Clinical effect of Wenyang Jianpi ointment in the treatment of chronic atrophic gastritis of spleen stomach weakness | Original article |
| 53 | 温阳健脾汤治疗脾胃虚弱型慢性萎缩性胃炎的临床疗效研究 | [Clinical efficacy of Wenyang Jianpi Decoction for chronic atrophic gastritis with spleen–stomach deficiency] | Author-prepared translation |
| 54 | 消化复宁汤联合瑞巴派特治疗慢性萎缩性胃炎肝郁脾虚证疗效及对患者胃镜组织学和H | [Efficacy of Xiaohua Funing Decoction combined with rebamipide for chronic atrophic gastritis with liver-depression and spleen-deficiency syndrome and its effects on gastroscopic histology and H. pylori clearance] | Author-prepared translation |
| 55 | 益气活血法对慢性萎缩性胃炎癌前病变Ki-67和肿瘤标记物的影响 | Effect of Therapy of Replenishing Qi and Activating Blood on Ki-67 and Tumor Markers in Precancerous Lesions of Chronic Atrophic Gastritis | Original article |
| 56 | 益胃化浊解毒方联合维酶素片治疗慢性萎缩性胃炎胃癌前病变疗效及对Ki-67蛋白表达的影响 | Curative effect of Yiwei Huazhuo Jiedu Decoction and vitacoenzyme tablets on precancerous lesions of chronic atrophic gastritis and its effect on the expression of Ki-67 protein | Original article |
| 57 | 益胃健脾汤联合西药治疗脾胃虚弱型慢性萎缩性胃炎42例 | [Yiwei Jianpi Decoction combined with Western medicine for 42 cases of chronic atrophic gastritis with spleen–stomach deficiency] | Author-prepared translation |
| 58 | 郁金、三七分别联合叶酸治疗胃癌前病变的疗效观察 | Effects of Radix Curcumae and Notoginseng combined with Folic Acid for the treatment of premalignant gastric lesions | Original article |
| 59 | 贞芪扶正胶囊治疗慢性萎缩性胃炎疗效观察 | [Efficacy of Zhenqi Fuzheng Capsules for chronic atrophic gastritis] | Author-prepared translation |
| 60 | 中西医结合治疗慢性萎缩性胃炎36例 | [Integrated Chinese and Western medicine for 36 cases of chronic atrophic gastritis] | Author-prepared translation |
| 61 | 中西医结合治疗慢性萎缩性胃炎36例 | [Integrated Chinese and Western medicine for 36 cases of chronic atrophic gastritis] | Author-prepared translation |
| 62 | 中西医结合治疗脾胃虚弱型慢性萎缩性胃炎40例临床观察 | [Clinical observation of integrated Chinese and Western medicine for 40 cases of chronic atrophic gastritis with spleen–stomach deficiency] | Author-prepared translation |
| 63 | 中西医结合治疗幽门螺杆菌感染性慢性萎缩性胃炎58例 | [Integrated Chinese and Western medicine for 58 cases of chronic atrophic gastritis with H. pylori infection] | Author-prepared translation |
| 64 | 中医综合疗法联合叶酸片治疗慢性萎缩性胃炎的疗效研究 | [Efficacy of comprehensive traditional Chinese medicine therapy combined with folic acid tablets for chronic atrophic gastritis] | Author-prepared translation |
| 65 | 资生汤加减治疗慢性萎缩性胃炎伴肠上皮化生的临床研究 | Clinical therapy of Zisheng decoction recipe for chronic atrophic gastritis with intestinal metaplasia | Original article |
| 66 | 自拟百丹胃萎汤治疗慢性萎缩性胃炎的临床疗效 | Clinical efficacy of self-formulated Baidan Weiwei decoction in the treatment of chronic atrophic gastritis | Original article |
| 67 | 自拟调胃汤对慢性萎缩性胃炎患者MTL和SS的影响 | [Effects of a self-formulated Tiaowei Decoction on motilin and somatostatin in patients with chronic atrophic gastritis] | Author-prepared translation |
| 69 | 疏肝和胃法联合西药治疗慢性萎缩性胃炎的现代临床研究 | Modern Clinical Research of TCM Dispersing Stagnated Liver Qi for Regulating Stomach Method combined with Western Medicine in the Treatment of Chronic Atrophic Gastritis | Original article |
| 70 | 中医活血化瘀法联合西药治疗慢性萎缩性胃炎 | [Blood-activating and stasis-resolving traditional Chinese medicine therapy combined with Western medicine for chronic atrophic gastritis] | Author-prepared translation |
| 71 | 中西医结合治疗脾胃虚寒型慢性萎缩性胃炎合并HP感染临床观察 | Efficiency observation of integrated therapy of traditional Chinese and western medicine on chronic atrophic gastritis with HP infection of deficiency cold of spleen and stomach | Original article |
| 72 | 中西医结合（养阴活胃合剂与标准根除Hp治疗）治疗慢性萎缩性胃炎129例的临床研究 | [Clinical study of integrated Chinese and Western medicine (Yangyin Huowei mixture plus standard H. pylori eradication therapy) for 129 cases of chronic atrophic gastritis] | Author-prepared translation |
| 73 | 摩罗丹联合叶酸治疗慢性萎缩性胃炎伴异型增生的临床研究 | Clinical effect of Moluodan combined with folic acid on chronic atrophic gastritis with atypical hyperplasia | Original article |
| 74 | 柴胡疏肝散合清中汤加味治疗肝胃不和型慢性萎缩性胃炎临床研究 | Clinical Study on Bupleurum Liver-Soothing Powder Combined with Supplemented Decoction for Clearing Middle Energizer in Treating Chronic Atrophic Gastritis with Liver-Stomach Disharmony Syndrome | Original article |
| 75 | 雷贝拉唑联合柴胡舒肝丸治疗老年慢性萎缩性胃炎疗效及对血清胃泌素和内皮素的影响 | [Efficacy of rabeprazole combined with Chaihu Shugan Pill for chronic atrophic gastritis in older adults and its effects on serum gastrin and endothelin] | Author-prepared translation |
| 76 | 化痰消瘀方对慢性萎缩性胃炎合并肠上皮化生患者的疗效及对胃镜病理的影响 | [Efficacy of Huatan Xiaoyu Formula for chronic atrophic gastritis with intestinal metaplasia and its effects on gastroscopic pathology] | Author-prepared translation |
| 77 | 中药联合埃索美拉唑治疗慢性萎缩性胃炎临床观察 | [Clinical observation of Chinese herbal medicine combined with esomeprazole for chronic atrophic gastritis] | Author-prepared translation |
| 78 | 自拟胃虚瘀阻方治疗慢性萎缩性胃炎临床观察 | Self-made Weixu Yuzu Prescription in the Treatment of Chronic Atrophic Gastritis | Original article |
| 79 | 升阳益胃汤治疗幽门螺杆菌阳性胃癌前病变临床观察 | Clinical observation on Shengyang Yiwei Decoction in the treatment of Helicobacter pylori positive gastric precancerous lesions | Original article |
| 80 | 七方胃痛颗粒对脾虚型萎缩性胃炎42例的疗效观察 | [Efficacy of Qifang Weitong Granules for 42 cases of spleen-deficiency-type atrophic gastritis] | Author-prepared translation |
| 81 | 中西医结合治疗慢性萎缩性胃炎临床观察 | [Clinical observation of integrated Chinese and Western medicine for chronic atrophic gastritis] | Author-prepared translation |
| 82 | 脘腹祛痛汤结合埃索美拉唑镁肠溶片治疗慢性萎缩性胃炎临床研究 | Clinical study on Wanfu-Qutong Decoction combined with esomeprazole tablets for atrophic gastritis | Original article |
| 83 | 益胃宁胶囊联合瑞巴派特片治疗慢性萎缩性胃炎脾胃气虚证临床研究 | Clinical Study on the Effects of Yiweining Capsules Combined with Rebamipide Tablet in Treating Chronic Atrophic Gastritis with Spleen-stomach Qi Deficiency Syndrome | Original article |
| 84 | 二陈平胃散治疗慢性萎缩性胃炎的疗效观察及其对血清胃泌素17、胃蛋白酶原的影响 | [Efficacy of Erchen Pingwei Powder for chronic atrophic gastritis and its effects on serum gastrin-17 and pepsinogen] | Author-prepared translation |
| 85 | 中西医结合治疗慢性萎缩性胃炎50例临床分析 | [Clinical analysis of integrated Chinese and Western medicine for 50 cases of chronic atrophic gastritis] | Author-prepared translation |
| 86 | “健胃消萎灵汤”治疗幽门螺旋杆菌阳性慢性萎缩性胃炎60例 | [“Jianwei Xiaoweiling Decoction” for 60 cases of H. pylori-positive chronic atrophic gastritis] | Author-prepared translation |
| 87 | 化痰消瘀方加味治疗胃食管反流病伴慢性萎缩性胃炎的疗效观察 | Curative effect observation of modified Huatan Xiaoyu decoction on treatment of gastroesophageal reflux disease with chronic atrophic gastritis | Original article |
| 89 | “柴归汤”辅助治疗气虚血瘀型慢性萎缩性胃炎36例临床研究 | [Clinical study of “Chaigui Decoction” as adjunctive therapy for 36 cases of chronic atrophic gastritis with qi deficiency and blood stasis] | Author-prepared translation |
| 90 | 舒胃方联合黛力新治疗慢性萎缩性胃炎伴焦虑抑郁状态的效果研究 | [Effects of Shuwei Formula combined with Deanxit on chronic atrophic gastritis with anxiety and depression] | Author-prepared translation |

Source labels: “Original article” indicates that an English title was printed in the supplied full-text source; “Official journal metadata” indicates a title verified on an official journal/publisher source; “Author-prepared translation” indicates a translation prepared when no verifiable published English title was identified. Square brackets are used only in this supplementary table to identify author-prepared translations; the main manuscript reference list presents the corresponding English titles without brackets for consistent Frontiers-style readability.
