# Supplementary material for: Chinese herbal medicine combined with conventional therapy for gastric precancerous lesions: a systematic review and meta-analysis
Source: Oncol Rev. 2026 Oct 19;20:1947905. doi: 10.3389/or.2026.1947905 (PMC13724460; doi:10.3389/or.2026.1947905)
Supplement: Supplementary file 5 [file Table1.docx]

**Supplementary Table S1. Detailed characteristics of all 69 included studies**

*Reconstructed and source-verified from the supplied 69 full-text records; original panel architecture and column headers retained.*

**Panel A. Study and report characteristics**

| **Study ID** | **First author** | **Year** | **Journal/source** | **Country** | **Multicenter** | **Funding** | **Registration** | **Ethics approval** |
| --- | --- | --- | --- | --- | --- | --- | --- | --- |
| S1 | Wei | 2025 | Chinese Typical Case Database | China | NR | NR | NR | NR |
| S2 | Xi | 2025 | The Medical Journal of Thepresent Clinical | China | NR | NR | NR | NR |
| S3 | Zhang | 2022 | Shaanxi Journal of Traditional Chinese Medicine | China | NR | Shaanxi Administration of Traditional Chinese Medicine (JCPT033) | NR | NR |
| S4 | Shen | 2017 | Modern Journal of Integrated Traditional Chinese and Western Medicine | China | NR | NR | NR | NR |
| S5 | Yuan | 2026 | Henan Traditional Chinese Medicine | China | NR | Henan TCM Inheritance and Innovation Talent Program (2021 grants) | NR | Reported (ethics approval stated; identifier NR) |
| S6 | You | 2021 | Acta Chinese Medicine | China | NR | Jiangsu Administration of Traditional Chinese Medicine (YB2015166) | NR | Reported (ethics approval stated; identifier NR) |
| S7 | Gao | 2022 | Inner Mongolia Journal of Traditional Chinese Medicine | China | NR | NR | NR | NR |
| S8 | Yi | 2022 | Shanxi Journal of Traditional Chinese Medicine | China | NR | Hunan Administration of Traditional Chinese Medicine (2021102) | NR | NR |
| S9 | Zhang | 2016 | Chinese Journal of Integrated Traditional and Western Medicine on Digestion | China | NR | Jiangsu Administration of Traditional Chinese Medicine (YB2015163) | NR | NR |
| S10 | Zhang | 2020 | Chinese Journal of Integrated Traditional and Western Medicine on Digestion | China | NR | NR | NR | NR |
| S11 | Huang | 2020 | Modern Journal of Integrated Traditional Chinese and Western Medicine | China | NR | NR | NR | NR |
| S13 | Liu | 2005 | Chinese Journal of Integrated Traditional and Western Medicine on Digestion | China | NR | NR | NR | NR |
| S14 | Liu | 2017 | Shandong Journal of Traditional Chinese Medicine | China | NR | NR | NR | NR |
| S15 | Wang | 2025 | International Journal of Traditional Chinese Medicine | China | NR | Shaanxi Administration of Traditional Chinese Medicine (SZY-KJCYC-2023-044) | NR | NR |
| S16 | Zhuang | 2011 | China Modern Medicine | China | NR | NR | NR | NR |
| S17 | Wang | 2021 | China Medical Herald | China | NR | Liaoning Natural Science Foundation guidance project (2019-ZD-0960) | NR | NR |
| S18 | Zhang | 2013 | Chinese Journal of Integrated Traditional and Western Medicine on Digestion | China | NR | NR | NR | NR |
| S19 | Shen | 2021 | Practical Clinical Journal of Integrated Traditional Chinese and Western Medicine | China | Yes | NR | NR | NR |
| S20 | Wang | 2023 | Shenzhen Journal of Integrated Traditional Chinese and Western Medicine | China | NR | NR | NR | NR |
| S21 | Zhang | 2025 | Chinese Journal of Integrated Traditional and Western Medicine on Digestion | China | NR | Huizhou Science and Technology Program (180525101740492) | NR | NR |
| S22 | Jia | 2021 | Chinese Journal of Integrated Traditional and Western Medicine on Digestion | China | NR | NR | NR | Reported (ethics approval stated; identifier NR) |
| S24 | Yang | 2024 | Practical Clinical Journal of Integrated Traditional Chinese and Western Medicine | China | Yes | NR | NR | NR |
| S26 | Wang | 2018 | World Journal of Integrated Traditional and Western Medicine | China | Yes | NR | NR | Reported (ethics approval stated; identifier NR) |
| S27 | Ning | 2016 | Chinese Journal of Experimental Traditional Medical Formulae | China | NR | Xining health research fund (2013ZYXD312) | NR | NR |
| S28 | Qi | 2024 | Fertility & Health | China | NR | NR | NR | Reported (ethics approval stated; identifier NR) |
| S29 | Dong | 2024 | Journal of Guangxi University of Chinese Medicine | China | Yes | Anhui collaborative TCM-Western medicine program (0708-2021) and related program support | NR | Reported (ethics approval stated; identifier NR) |
| S30 | Yang | 2017 | Acta Chinese Medicine | China | NR | Zhengzhou Science and Technology Program (X2013S0264) | NR | Reported (ethics approval stated; identifier NR) |
| S31 | Xu | 2018 | Acta Chinese Medicine | China | NR | Wuhan Science and Technology Program (2011610383414) | NR | NR |
| S32 | Liu | 2010 | Shanghai Journal of Traditional Chinese Medicine | China | NR | NR | NR | NR |
| S33 | Xing | 2004 | Chinese Journal of Integrated Traditional and Western Medicine on Digestion | China | NR | NR | NR | NR |
| S34 | Jiang | 2025 | Chinese Journal of Clinical Rational Drug Use | China | NR | Guangzhou TCM/integrative medicine project (20222A010079) and Panyu project (2021-Z04-019) | NR | Reported (ethics approval stated; identifier NR) |
| S35 | Zhang | 2021 | Pharmacy Weekly | China | NR | NR | NR | NR |
| S37 | Bu | 2019 | Modern Journal of Integrated Traditional Chinese and Western Medicine | China | NR | NR | NR | NR |
| S38 | Yu | 2023 | Journal of Guangzhou University of Traditional Chinese Medicine | China | NR | Shaanxi Natural Science Basic Research Program (2018JQ8009) and national TCM mentorship program | NR | Reported (ethics approval stated; identifier NR) |
| S39 | Xu | 2021 | Modern Journal of Integrated Traditional Chinese and Western Medicine | China | NR | National TCM clinical research base project and Hebei TCM research project (2018016) | NR | Reported (ethics approval stated; identifier NR) |
| S40 | Li | 2019 | TCM Research | China | NR | NR | NR | NR |
| S42 | Sun | 2017 | Chinese Journal of Integrated Traditional and Western Medicine on Digestion | China | NR | Ningbo Science and Technology Bureau social development project (2013C50045) | NR | NR |
| S43 | Wang | 2010 | Evaluation and Analysis of Drug-Use in Hospitals of China | China | NR | NR | NR | NR |
| S44 | Zhang | 2008 | TCM Research | China | NR | NR | NR | NR |
| S45 | Zhang | 2004 | Acta Academiae Medicinae Weifang | China | NR | NR | NR | NR |
| S46 | Fu | 2020 | Chinese Journal of Ethnomedicine and Ethnopharmacy | China | NR | NR | NR | Reported (ethics approval stated; identifier NR) |
| S47 | Wang | 2003 | Modern Journal of Integrated Traditional Chinese and Western Medicine | China | NR | NR | NR | NR |
| S48 | Sun | 2020 | Journal of Baotou Medical College | China | NR | NR | NR | NR |
| S49 | Wu | 2017 | China Journal of Chinese Materia Medica | China | NR | Henan Department of Education natural science project (2010A360011) | NR | NR |
| S50 | Lin | 2023 | Health Medicine Research and Practice | China | NR | NR | NR | Reported (ethics approval stated; identifier NR) |
| S51 | Wang | 2021 | Journal of Chinese Medicinal Materials | China | NR | NR | NR | NR |
| S52 | Zhang | 2023 | Histology and Histopathology | China | NR | NR | NR | Reported (ethics approval stated; identifier NR) |
| H01 | Cai | 2016 | Guangming Journal of Chinese Medicine | China | NR | NR | NR | NR |
| H02 | Shen | 2007 | Guangdong Medical Journal | China | NR | NR | NR | NR |
| H03 | Ding | 2019 | Shanxi Journal of Traditional Chinese Medicine | China | NR | NR | NR | NR |
| H04 | Du | 2015 | XINJIANG JOURNAL OF TRADITIONAL CHINESE MEDICINE | China | Yes | NR | NR | NR |
| H05 | Feng | 2011 | Hebei Journal of Traditional Chinese Medicine | China | NR | NR | NR | NR |
| H06 | Guo | 2023 | Henan Traditional Chinese Medicine | China | NR | Zhumadian Science and Technology Program (201800135) | NR | Reported (ethics approval stated; identifier NR) |
| H07 | Hu | 2016 | Modern Journal of Integrated Traditional Chinese and Western Medicine | China | NR | NR | NR | NR |
| H08 | Hu | 2019 | Journal of Chinese Medicinal Materials | China | NR | NR | NR | NR |
| H09 | Jin | 2012 | Jilin Journal of Traditional Chinese Medicine | China | NR | NR | NR | NR |
| H10 | Ke | 2025 | Chinese Medicine Modern Distance Education of China | China | NR | NR | NR | Reported (ethics approval stated; identifier NR) |
| H11 | Peng | 2022 | Modern Journal of Integrated Traditional Chinese and Western Medicine | China | Yes | Hebei Administration of Traditional Chinese Medicine (2018075) | NR | NR |
| H12 | Wang | 2012 | Modern Digestion & Intervention | China | NR | Guangxi Natural Science Foundation (0991156) | NR | NR |
| H13 | Xiang | 2007 | Practical Clinical Journal of Integrated Traditional Chinese and Western Medicine | China | NR | NR | NR | NR |
| H14 | Xu | 2021 | International Journal of Traditional Chinese Medicine | China | NR | Beijing Municipal Science and Technology Commission (Z161100000516120) | NR | Reported (ethics approval stated; identifier NR) |
| H15 | Ye | 2024 | Chinese Journal of Information on Traditional Chinese Medicine | China | NR | Fujian University of Traditional Chinese Medicine clinical project (XB2022032) | NR | Reported (ethics approval stated; identifier NR) |
| H16 | Ye | 2020 | Chinese Journal of Traditional Medical Science and Technology | China | NR | Wenzhou Science and Technology Program (Y20160217) | NR | Reported (ethics approval stated; identifier NR) |
| H17 | Zhu | 2009 | Chinese Journal of Primary Medicine and Pharmacy | China | NR | NR | NR | NR |
| H18 | Zhu | 2010 | Acta Chinese Medicine and Pharmacology | China | NR | Wenzhou Science and Technology Bureau project (Y20090227) | NR | NR |
| H19 | Zhu | 2021 | Chinese Journal of Integrated Traditional and Western Medicine on Digestion | China | NR | Jiangsu Administration of Traditional Chinese Medicine (YB2015166) | NR | NR |
| H20 | Zou | 2024 | J Dig Dis | China | Multicenter | NR | ChiCTR1800016087 | Reported (ethics approval stated; identifier NR) |
| U02 | Xu | 2026 | Jiangsu Journal of Traditional Chinese Medicine | China | NR | NR | NR | Reported (ethics approval stated; identifier NR) |
| U04 | Wang | 2026 | JIAN KANG SHI SHANG | China | NR | Xingtai key R&D self-funded project (identifier not fully reported in supplied text) | NR | NR |

**Panel B. Diagnostic and participant characteristics**

| **Study ID** | **First author** | **Year** | **Sample size calculation** | **Disease subtype / population** | **Diagnostic criteria** | **Randomised n (T)** | **Randomised n (C)** | **Mean age, years (T)** | **Mean age, years (C)** | **Male, n (T)** | **Male, n (C)** | **H. pylori status** |
| --- | --- | --- | --- | --- | --- | --- | --- | --- | --- | --- | --- | --- |
| S1 | Wei | 2025 | NR | CAG | Gastroscopy/endoscopy plus gastric mucosal histopathology/biopsy; source-reported diagnostic consensus/guideline where specified. | 40 | 40 | 75.79 ± 2.35 | 76.05 ± 2.51 | 24 | 22 | NR |
| S2 | Xi | 2025 | NR | CAG with IM | Gastroscopy/endoscopy plus gastric mucosal histopathology/biopsy; source-reported diagnostic consensus/guideline where specified. | 30 | 30 | 52.35 ± 6.56 | 51.98 ± 6.42 | 20 | 17 | NR |
| S3 | Zhang | 2022 | NR | CAG | Gastroscopy/endoscopy plus gastric mucosal histopathology/biopsy; source-reported diagnostic consensus/guideline where specified. | 35 | 35 | 50.06 ± 3.17 | 51.76 ± 6.24 | 18 | 21 | NR |
| S4 | Shen | 2017 | NR | PLGC | Gastroscopy/endoscopy plus gastric mucosal histopathology/biopsy; source-reported diagnostic consensus/guideline where specified. | 60 | 60 | 57.62 ± 6.73 | 57.48 ± 6.70 | 37 | 35 | NR |
| S5 | Yuan | 2026 | NR | CAG | Gastroscopy/endoscopy plus gastric mucosal histopathology/biopsy; source-reported diagnostic consensus/guideline where specified. | 60 | 60 | 56.79 ± 8.13 | 56.23 ± 8.67 | 38 | 35 | NR |
| S6 | You | 2021 | NR | PLGC | Gastroscopy/endoscopy plus gastric mucosal histopathology/biopsy; source-reported diagnostic consensus/guideline where specified. | 35 | 33 | 53.00 ± 10.06 | 51.00 ± 7.89 | 18 | 15 | NR |
| S7 | Gao | 2022 | NR | PLGC | Gastroscopy/endoscopy plus gastric mucosal histopathology/biopsy; source-reported diagnostic consensus/guideline where specified. | 40 | 40 | 54.48 ± 1.95 | 54.44 ± 1.91 | 23 | 24 | NR |
| S8 | Yi | 2022 | NR | CAG with dysplasia | Gastroscopy/endoscopy plus gastric mucosal histopathology/biopsy; source-reported diagnostic consensus/guideline where specified. | 35 | 35 | 45.61 ± 7.15 | 44.77 ± 6.96 | 16 | 17 | Positive |
| S9 | Zhang | 2016 | NR | CAG with bile reflux | Gastroscopy/endoscopy plus gastric mucosal histopathology/biopsy; source-reported diagnostic consensus/guideline where specified. | 62 | 65 | NR | NR | NR | NR | NR |
| S10 | Zhang | 2020 | NR | CAG with erosion | Gastroscopy/endoscopy plus gastric mucosal histopathology/biopsy; source-reported diagnostic consensus/guideline where specified. | 50 | 50 | 42.86 ± 5.33 | 42.38 ± 4.92 | 26 | 26 | NR |
| S11 | Huang | 2020 | NR | H. pylori-positive CAG | Gastroscopy/endoscopy plus gastric mucosal histopathology/biopsy; source-reported diagnostic consensus/guideline where specified. | 45 | 45 | 53.5 ± 4.7 | 54.3 ± 4.5 | 26 | 25 | Positive |
| S13 | Liu | 2005 | NR | CAG | Gastroscopy/endoscopy plus gastric mucosal histopathology/biopsy; source-reported diagnostic consensus/guideline where specified. | 50 | 50 | NR | NR | 31 | 33 | NR |
| S14 | Liu | 2017 | NR | CAG | Gastroscopy/endoscopy plus gastric mucosal histopathology/biopsy; source-reported diagnostic consensus/guideline where specified. | 50 | 50 | NR | NR | NR | NR | NR |
| S15 | Wang | 2025 | NR | H. pylori-related CAG | Gastroscopy/endoscopy plus gastric mucosal histopathology/biopsy; source-reported diagnostic consensus/guideline where specified. | 59 | 59 | 52.61 ± 8.55 | 52.30 ± 8.79 | 32 | 34 | Negative/negative subgroup reported |
| S16 | Zhuang | 2011 | NR | CAG | Gastroscopy/endoscopy plus gastric mucosal histopathology/biopsy; source-reported diagnostic consensus/guideline where specified. | 50 | 50 | 49 | 50 | 37 | 35 | NR |
| S17 | Wang | 2021 | NR | CAG | Gastroscopy/endoscopy plus gastric mucosal histopathology/biopsy; source-reported diagnostic consensus/guideline where specified. | 60 | 60 | 58.60 ± 7.24 | 59.48 ± 8.06 | 37 | 35 | NR |
| S18 | Zhang | 2013 | NR | CAG | Gastroscopy/endoscopy plus gastric mucosal histopathology/biopsy; source-reported diagnostic consensus/guideline where specified. | 30 | 30 | 54.00 ± 8.20 | 55.43 ± 9.20 | 10 | 10 | NR |
| S19 | Shen | 2021 | NR | CAG | Gastroscopy/endoscopy plus gastric mucosal histopathology/biopsy; source-reported diagnostic consensus/guideline where specified. | 49 | 49 | 56.10 ± 6.78 | 56.39 ± 6.82 | 32 | 29 | NR |
| S20 | Wang | 2023 | NR | CAG with erosion | Gastroscopy/endoscopy plus gastric mucosal histopathology/biopsy; source-reported diagnostic consensus/guideline where specified. | 43 | 43 | 59.33 ± 2.16 | 59.29 ± 2.15 | 24 | 23 | NR |
| S21 | Zhang | 2025 | NR | H. pylori-positive CAG | Gastroscopy/endoscopy plus gastric mucosal histopathology/biopsy; source-reported diagnostic consensus/guideline where specified. | 53 | 53 | 48.11 ± 9.35 | 47.83 ± 9.08 | 32 | 29 | Positive |
| S22 | Jia | 2021 | NR | H. pylori-positive CAG | Gastroscopy/endoscopy plus gastric mucosal histopathology/biopsy; source-reported diagnostic consensus/guideline where specified. | 55 | 55 | 48.6 ± 10.4 | 48.2 ± 10.7 | 30 | 32 | Positive |
| S24 | Yang | 2024 | NR | H. pylori-positive CAG | Gastroscopy/endoscopy plus gastric mucosal histopathology/biopsy; source-reported diagnostic consensus/guideline where specified. | 50 | 50 | 50.85 ± 5.03 | 50.74 ± 5.12 | 28 | 26 | Positive |
| S26 | Wang | 2018 | NR | CAG with primary bile reflux | Gastroscopy/endoscopy plus gastric mucosal histopathology/biopsy; source-reported diagnostic consensus/guideline where specified. | 42 | 36 | 41 | 45.9 | 18 | 17 | NR |
| S27 | Ning | 2016 | NR | CAG | Gastroscopy/endoscopy plus gastric mucosal histopathology/biopsy; source-reported diagnostic consensus/guideline where specified. | 55 | 55 | 56.78 ± 6.12 | 55.43 ± 6.68 | 31 | 29 | NR |
| S28 | Qi | 2024 | NR | H. pylori-positive CAG | Gastroscopy/endoscopy plus gastric mucosal histopathology/biopsy; source-reported diagnostic consensus/guideline where specified. | 34 | 34 | 51.44 ± 9.22 | 51.25 ± 9.36 | 17 | 18 | Positive |
| S29 | Dong | 2024 | NR | CAG | Gastroscopy/endoscopy plus gastric mucosal histopathology/biopsy; source-reported diagnostic consensus/guideline where specified. | 43 | 43 | 48.12 ± 18.67 | 50.21 ± 16.78 | 25 | 23 | NR |
| S30 | Yang | 2017 | NR | H. pylori-positive CAG in older adults | Gastroscopy/endoscopy plus gastric mucosal histopathology/biopsy; source-reported diagnostic consensus/guideline where specified. | 38 | 38 | 70.07 ± 4.19 | 69.27 ± 4.25 | 19 | 18 | Positive |
| S31 | Xu | 2018 | NR | H. pylori-positive CAG | Gastroscopy/endoscopy plus gastric mucosal histopathology/biopsy; source-reported diagnostic consensus/guideline where specified. | 120 | 120 | 44.32 ± 4.54 | 45.11 ± 4.76 | 75 | 76 | Positive |
| S32 | Liu | 2010 | NR | CAG with IM | Gastroscopy/endoscopy plus gastric mucosal histopathology/biopsy; source-reported diagnostic consensus/guideline where specified. | 50 | 50 | 46.45 ± 4.78 | 45.77 ± 5.02 | 30 | 29 | NR |
| S33 | Xing | 2004 | NR | CAG with IM | Gastroscopy/endoscopy plus gastric mucosal histopathology/biopsy; source-reported diagnostic consensus/guideline where specified. | 93 | 89 | NR | NR | NR | NR | NR |
| S34 | Jiang | 2025 | NR | CAG | Gastroscopy/endoscopy plus gastric mucosal histopathology/biopsy; source-reported diagnostic consensus/guideline where specified. | 30 | 30 | 49.00 ± 3.80 | 47.50 ± 3.75 | 18 | 17 | NR |
| S35 | Zhang | 2021 | NR | CAG | Gastroscopy/endoscopy plus gastric mucosal histopathology/biopsy; source-reported diagnostic consensus/guideline where specified. | 30 | 30 | 49.41 ± 3.26 | 49.67 ± 3.31 | 17 | 18 | NR |
| S37 | Bu | 2019 | NR | CAG | Gastroscopy/endoscopy plus gastric mucosal histopathology/biopsy; source-reported diagnostic consensus/guideline where specified. | 57 | 57 | 48.97 ± 6.38 | 49.32 ± 6.46 | 24 | 26 | Positive |
| S38 | Yu | 2023 | NR | PLGC | Gastroscopy/endoscopy plus gastric mucosal histopathology/biopsy; source-reported diagnostic consensus/guideline where specified. | 37 | 37 | 54.01 ± 2.12 | 53.19 ± 1.78 | 16 | 19 | NR |
| S39 | Xu | 2021 | NR | PLGC | Gastroscopy/endoscopy plus gastric mucosal histopathology/biopsy; source-reported diagnostic consensus/guideline where specified. | 51 | 51 | 58.2 ± 5.6 | 59.1 ± 6.1 | 35 | 34 | NR |
| S40 | Li | 2019 | NR | CAG | Gastroscopy/endoscopy plus gastric mucosal histopathology/biopsy; source-reported diagnostic consensus/guideline where specified. | 42 | 42 | 39.62 ± 2.54 | 39.50 ± 2.42 | 24 | 25 | NR |
| S42 | Sun | 2017 | NR | PLGC | Gastroscopy/endoscopy plus gastric mucosal histopathology/biopsy; source-reported diagnostic consensus/guideline where specified. | 64 | 32 | 54.87 ± 10.38 (Curcuma arm); 52.25 ± 9.33 (Notoginseng arm) | 53.09 ± 10.54 | 21; 18 | 20 | NR |
| S43 | Wang | 2010 | NR | CAG | Gastroscopy/endoscopy plus gastric mucosal histopathology/biopsy; source-reported diagnostic consensus/guideline where specified. | 84 | 42 | 45.5 | 45.2 | 43 | 22 | NR |
| S44 | Zhang | 2008 | NR | CAG | Gastroscopy/endoscopy plus gastric mucosal histopathology/biopsy; source-reported diagnostic consensus/guideline where specified. | 36 | 34 | NR | NR | NR | NR | NR |
| S45 | Zhang | 2004 | NR | H. pylori-positive CAG | Gastroscopy/endoscopy plus gastric mucosal histopathology/biopsy; source-reported diagnostic consensus/guideline where specified. | 36 | 34 | 48 | 47 | 22 | 20 | Positive |
| S46 | Fu | 2020 | NR | CAG | Gastroscopy/endoscopy plus gastric mucosal histopathology/biopsy; source-reported diagnostic consensus/guideline where specified. | 40 | 40 | 47.35 ± 2.81 | 47.32 ± 2.78 | 21 | 22 | NR |
| S47 | Wang | 2003 | NR | H. pylori-related CAG | Gastroscopy/endoscopy plus gastric mucosal histopathology/biopsy; source-reported diagnostic consensus/guideline where specified. | 58 | 27 | 53 | 52 | 35 | 14 | NR |
| S48 | Sun | 2020 | NR | H. pylori-positive CAG | Gastroscopy/endoscopy plus gastric mucosal histopathology/biopsy; source-reported diagnostic consensus/guideline where specified. | 120 | 60 | 44.2 (intervention I); 43.3 (intervention II) | 42.3 | 27; 24 | 22 | Positive |
| S49 | Wu | 2017 | NR | CAG with IM | Gastroscopy/endoscopy plus gastric mucosal histopathology/biopsy; source-reported diagnostic consensus/guideline where specified. | 49 | 49 | 44.3 ± 10.2 | 48.5 ± 13.7 | 22 | 24 | NR |
| S50 | Lin | 2023 | NR | CAG | Gastroscopy/endoscopy plus gastric mucosal histopathology/biopsy; source-reported diagnostic consensus/guideline where specified. | 40 | 40 | 46.23 ± 4.71 | 45.78 ± 4.65 | 23 | 21 | NR |
| S51 | Wang | 2021 | NR | CAG | Gastroscopy/endoscopy plus gastric mucosal histopathology/biopsy; source-reported diagnostic consensus/guideline where specified. | 55 | 55 | 44.74 ± 11.23 | 45.36 ± 12.25 | 35 | 33 | Positive |
| S52 | Zhang | 2023 | Reported | H. pylori-positive chronic active gastritis with atrophy | Gastroscopy/endoscopy plus gastric mucosal histopathology/biopsy; source-reported diagnostic consensus/guideline where specified. | 38 | 38 | 49.05 ± 6.62 | 48.79 ± 6.77 | 25 | 22 | Positive |
| H01 | Cai | 2016 | NR | NR | Gastroscopy/endoscopy plus gastric mucosal histopathology/biopsy; source-reported diagnostic consensus/guideline where specified. | 40 | 40 | 47.2 ± 14.8 | 45.2 ± 13.6 | 21 | 18 | NR |
| H02 | Shen | 2007 | NR | NR | Gastroscopy/endoscopy plus gastric mucosal histopathology/biopsy; source-reported diagnostic consensus/guideline where specified. | 69 | 63 | NR | NR | 42 | 40 | NR |
| H03 | Ding | 2019 | NR | NR | Gastroscopy/endoscopy plus gastric mucosal histopathology/biopsy; source-reported diagnostic consensus/guideline where specified. | 51 | 52 | 54.18 ± 5.48 | 55.44 ± 6.57 | 20 | 20 | NR |
| H04 | Du | 2015 | NR | NR | Gastroscopy/endoscopy plus gastric mucosal histopathology/biopsy; source-reported diagnostic consensus/guideline where specified. | 48 | 48 | 60.651 ± 6.862 | 60.674 ± 6.163 | 20 | 21 | Positive |
| H05 | Feng | 2011 | NR | NR | Gastroscopy/endoscopy plus gastric mucosal histopathology/biopsy; source-reported diagnostic consensus/guideline where specified. | 54 | 51 | 44 ± 5 | 43 ± 10 | 32 | 31 | NR |
| H06 | Guo | 2023 | NR | NR | Gastroscopy/endoscopy plus gastric mucosal histopathology/biopsy; source-reported diagnostic consensus/guideline where specified. | 45 | 45 | 45.32 ± 4.28 | 44.83 ± 4.36 | 28 | 26 | NR |
| H07 | Hu | 2016 | NR | NR | Gastroscopy/endoscopy plus gastric mucosal histopathology/biopsy; source-reported diagnostic consensus/guideline where specified. | 40 | 40 | 62.2 ± 5.9 | 63.1 ± 5.4 | 24 | 25 | NR |
| H08 | Hu | 2019 | NR | NR | Gastroscopy/endoscopy plus gastric mucosal histopathology/biopsy; source-reported diagnostic consensus/guideline where specified. | 60 | 60 | 55.02 ± 6.17 | 54.53 ± 7.42 | 37 | 34 | Positive |
| H09 | Jin | 2012 | NR | NR | Gastroscopy/endoscopy plus gastric mucosal histopathology/biopsy; source-reported diagnostic consensus/guideline where specified. | 73 | 61 | NR | NR | NR | NR | Positive |
| H10 | Ke | 2025 | NR | NR | Gastroscopy/endoscopy plus gastric mucosal histopathology/biopsy; source-reported diagnostic consensus/guideline where specified. | 35 | 35 | 53.35 ± 8.18 | 54.15 ± 8.01 | 16 | 15 | NR |
| H11 | Peng | 2022 | NR | NR | Gastroscopy/endoscopy plus gastric mucosal histopathology/biopsy; source-reported diagnostic consensus/guideline where specified. | 89 | 89 | 49.2 ± 10.4 | 48.7 ± 12.1 | 43 | 46 | Positive |
| H12 | Wang | 2012 | NR | NR | Gastroscopy/endoscopy plus gastric mucosal histopathology/biopsy; source-reported diagnostic consensus/guideline where specified. | 42 | 41 | 39.32 ± 7.62 | 41.62 ± 6.49 | 25 | 29 | NR |
| H13 | Xiang | 2007 | NR | NR | Gastroscopy/endoscopy plus gastric mucosal histopathology/biopsy; source-reported diagnostic consensus/guideline where specified. | 80 | 50 | 46 ± 10.4 | 47 ± 11.5 | 44 | 28 | NR |
| H14 | Xu | 2021 | NR | NR | Gastroscopy/endoscopy plus gastric mucosal histopathology/biopsy; source-reported diagnostic consensus/guideline where specified. | 53 | 53 | 57.15 ± 16.94 | 55.32 ± 14.73 | 30 | 29 | NR |
| H15 | Ye | 2024 | NR | NR | Gastroscopy/endoscopy plus gastric mucosal histopathology/biopsy; source-reported diagnostic consensus/guideline where specified. | 40 | 40 | 51.63 ± 4.52 | 52.86 ± 3.87 | 18 | 17 | Negative/negative subgroup reported |
| H16 | Ye | 2020 | NR | NR | Gastroscopy/endoscopy plus gastric mucosal histopathology/biopsy; source-reported diagnostic consensus/guideline where specified. | 100 | 100 | 56.59 ± 6.12 | 55.71 ± 5.43 | 54 | 58 | NR |
| H17 | Zhu | 2009 | NR | NR | Gastroscopy/endoscopy plus gastric mucosal histopathology/biopsy; source-reported diagnostic consensus/guideline where specified. | 28 | 22 | 53.2 ± 8.4 | 51.5 ± 11.3 | 12 | 10 | NR |
| H18 | Zhu | 2010 | NR | NR | Gastroscopy/endoscopy plus gastric mucosal histopathology/biopsy; source-reported diagnostic consensus/guideline where specified. | 30 | 30 | 48.25 | 50.42 | 12 | 16 | Positive |
| H19 | Zhu | 2021 | NR | NR | Gastroscopy/endoscopy plus gastric mucosal histopathology/biopsy; source-reported diagnostic consensus/guideline where specified. | 35 | 35 | 52.77 ± 13.58 | 57.26 ± 12.79 | 20 | 22 | NR |
| H20 | Zou | 2024 | Reported | CAG with moderate-to-severe atrophy and/or IM, with or without low-grade dysplasia; H. pylori negative at enrolment | Gastroscopy/endoscopy plus gastric mucosal histopathology/biopsy; source-reported diagnostic consensus/guideline where specified. | 84 | 168 | 56.49 ± 8.12 | 55.68 ± 8.59 | 37 | 88 | Negative at enrolment (including participants negative after prior eradication) |
| U02 | Xu | 2026 | Reported | NR | Gastroscopy/endoscopy plus gastric mucosal histopathology/biopsy; source-reported diagnostic consensus/guideline where specified. | 38 | 38 | 57.84 ± 9.79 | 55.45 ± 10.78 | 17 | 20 | NR |
| U04 | Wang | 2026 | NR | NR | Gastroscopy/endoscopy plus gastric mucosal histopathology/biopsy; source-reported diagnostic consensus/guideline where specified. | 50 | 50 | 55.1 ± 7.9 | 54.6 ± 8.2 | 26 | 28 | Positive |

Panel B notes: Demographic fields were reverified against all 69 source reports. NR is retained only when the required arm-specific value could not be verified from the source. Arm-specific mean age remained unavailable in 7/69 studies (S9, S13, S14, S33, S44, H02 and H09), and arm-specific male counts in 5/69 studies (S9, S14, S33, S44 and H09). For S27, H04, H10, H20 and U02, the source-reported demographic population differed from the full randomized sample: S27, 54/54 completers; H04, 43/43 completers; H10, 34/33 completers; H20, FAS 81/156; and U02, 36/35 completers. For H04, T/C demographics correspond to the eligible combined-treatment and Western-medicine arms. H10 male counts (16/15) were exactly derived from the source-reported male:female ratios (8:9 and 5:6, respectively) and the corresponding demographic denominators. H06 reported male/female counts of 28/27 and 26/29 despite stating n=45 per arm; the male counts were transcribed as reported, and this internal source inconsistency was not resolved by inference. For multi-arm studies S42 and S48, both eligible intervention arms are shown against their shared control.

**Panel C. Chinese herbal medicine intervention**

| **Study ID** | **First author** | **Year** | **Formula/intervention name** | **Formula type** | **Herbal composition (source-reported)** | **Botanical/Latin name (editorially standardized)** | **Symptom-based modification** | **Dose** | **Route / formulation** | **Frequency** | **Duration** |
| --- | --- | --- | --- | --- | --- | --- | --- | --- | --- | --- | --- |
| S1 | Wei | 2025 | Modified Banxia Xiexin decoction | Modified/individualized | 黄连 6 g; 大枣 10 g; 枳实 10 g; 半夏 12 g; 黄芩 12 g; 炙甘草 12 g; 蒲公英 15 g; 煅乌贼骨 15 g; 白花蛇舌草 15 g; 党参 16 g; 干姜 16 g | *黄连: Coptis chinensis Franch.; 大枣: Ziziphus jujuba Mill.; 枳实: Citrus aurantium L.; 半夏: Pinellia ternata (Thunb.) Makino; 黄芩: Scutellaria baicalensis Georgi; 甘草: Glycyrrhiza uralensis Fisch. ex DC.; 蒲公英: Taraxacum mongolicum Hand.-Mazz.; 乌贼骨: Sepiae Endoconcha (Sepia spp.; animal-derived); 白花蛇舌草: Scleromitrion diffusum (Willd.) R.J.Wang (syn. Hedyotis diffusa Willd.); 党参: Codonopsis pilosula (Franch.) Nannf.; 干姜: Zingiber officinale Roscoe* | Distension: 莱菔子 15 g and 砂仁 15 g; reflux/burning: 海螵蛸 15 g, 煅牡蛎 15 g, 煅瓦楞子 15 g and 龙胆草 10 g; pain: 延胡索 10 g and 白芍 10 g | 1 decoction/day; 400 mL reduced to 200 mL | Oral decoction | Twice daily | 4 weeks |
| S2 | Xi | 2025 | Syndrome-differentiated Chinese herbal medicine | Syndrome-differentiated | 脾胃虚弱方—党参 15 g; 黄芪 20 g; 白术 12 g; 茯苓 15 g; 陈皮 10 g; 木香 6 g; 砂仁 6 g; 炙甘草 6 g; 炒麦芽 15 g; 炒谷芽 15 g; 胃阴不足方—沙参 15 g; 麦冬 15 g; 玉竹 12 g; 生地黄 15 g; 白芍 15 g; 当归 10 g; 枸杞子 12 g; 川楝子 10 g; 炙甘草 6 g; 石斛 15 g; 炒麦芽 15 g; 浊毒内蕴方—黄连 6 g; 厚朴 10 g; 石菖蒲 10 g; 法半夏 10 g; 茯苓 15 g; 栀子 10 g; 芦根 20 g; 吴茱萸 3 g; 蒲公英 20 g; 白花蛇舌草 15 g; 炒莱菔子 15 g | *党参: Codonopsis pilosula (Franch.) Nannf.; 黄芪: Astragalus mongholicus Bunge; 白术: Atractylodes macrocephala Koidz.; 茯苓: Wolfiporia cocos (F.A.Wolf) Ryvarden & Gilb. (syn. Poria cocos); 陈皮: Citrus reticulata Blanco; 木香: Aucklandia costus Falc.; 砂仁: Wurfbainia villosa (Lour.) Skornick. & A.D.Poulsen (syn. Amomum villosum Lour.); 甘草: Glycyrrhiza uralensis Fisch. ex DC.; 麦芽: Hordeum vulgare L.; 谷芽: Oryza sativa L.; 沙参: Adenophora spp. (species not specified); 麦冬: Ophiopogon japonicus (Thunb.) Ker Gawl.; 玉竹: Polygonatum odoratum (Mill.) Druce; 地黄: Rehmannia glutinosa (Gaertn.) DC.; 白芍: Paeonia lactiflora Pall.; 当归: Angelica sinensis (Oliv.) Diels; 枸杞子: Lycium barbarum L.; 川楝子: Melia toosendan Siebold & Zucc.; 石斛: Dendrobium spp. (species not specified); 黄连: Coptis chinensis Franch.; 厚朴: Magnolia officinalis Rehder & E.H.Wilson; 石菖蒲: Acorus tatarinowii Schott; 半夏: Pinellia ternata (Thunb.) Makino; 栀子: Gardenia jasminoides J.Ellis; 芦根: Phragmites australis (Cav.) Trin. ex Steud.; 吴茱萸: Tetradium ruticarpum (A.Juss.) T.G.Hartley; 蒲公英: Taraxacum mongolicum Hand.-Mazz.; 白花蛇舌草: Scleromitrion diffusum (Willd.) R.J.Wang (syn. Hedyotis diffusa Willd.); 莱菔子: Raphanus sativus L.* | Prescription selected by syndrome | 1 decoction/day | Oral decoction | Twice daily, warm | 3 months |
| S3 | Zhang | 2022 | Modified Buxu Yangwei decoction | Modified/individualized | 党参 15 g; 砂仁 15 g; 黄芪 20 g; 熟附子 10 g; 丹参 10 g; 陈皮 10 g; 茯苓 10 g; 川石斛 8 g; 白术 8 g; 甘草 6 g | *党参: Codonopsis pilosula (Franch.) Nannf.; 砂仁: Wurfbainia villosa (Lour.) Skornick. & A.D.Poulsen (syn. Amomum villosum Lour.); 黄芪: Astragalus mongholicus Bunge; 附子: Aconitum carmichaelii Debeaux; 丹参: Salvia miltiorrhiza Bunge; 陈皮: Citrus reticulata Blanco; 茯苓: Wolfiporia cocos (F.A.Wolf) Ryvarden & Gilb. (syn. Poria cocos); 石斛: Dendrobium spp. (species not specified); 白术: Atractylodes macrocephala Koidz.; 甘草: Glycyrrhiza uralensis Fisch. ex DC.* | Blood stasis: 三七 6 g; severe pain: 木香 5 g and 延胡索 8 g; phlegm-dampness: 法半夏 8 g; acid regurgitation: 吴茱萸 10 g; poor appetite: 山楂 10 g, 神曲 10 g and 麦芽 10 g; distension: 枳壳 10 g | 1 decoction/day; 500 mL reduced to 300 mL | Oral decoction | Morning and evening | 3 months |
| S4 | Shen | 2017 | Shenzhi Xiaowei decoction | Fixed formula | 人参 20 g; 枳实 20 g; 茯苓 20 g; 当归 15 g; 陈皮 15 g; 木香 15 g; 柴胡 15 g; 丹参 15 g; 白术 15 g; 白芍 10 g; 法半夏 10 g; 白花蛇舌草 10 g; 炙甘草 8 g | *人参: Panax ginseng C.A.Mey.; 枳实: Citrus aurantium L.; 茯苓: Wolfiporia cocos (F.A.Wolf) Ryvarden & Gilb. (syn. Poria cocos); 当归: Angelica sinensis (Oliv.) Diels; 陈皮: Citrus reticulata Blanco; 木香: Aucklandia costus Falc.; 柴胡: Bupleurum chinense DC.; 丹参: Salvia miltiorrhiza Bunge; 白术: Atractylodes macrocephala Koidz.; 白芍: Paeonia lactiflora Pall.; 半夏: Pinellia ternata (Thunb.) Makino; 白花蛇舌草: Scleromitrion diffusum (Willd.) R.J.Wang (syn. Hedyotis diffusa Willd.); 甘草: Glycyrrhiza uralensis Fisch. ex DC.* | None reported | 1 decoction/day | Oral decoction | Twice daily | 4 months |
| S5 | Yuan | 2026 | Modified Chaihu Jia Longgu Muli decoction | Fixed formula | 海螵蛸 30 g; 丹参 30 g; 酸枣仁 30 g; 龙骨 20 g; 牡蛎 20 g; 法半夏 15 g; 茯苓 15 g; 葛根 15 g; 黄芩 12 g; 党参 12 g; 桂枝 12 g; 白术 12 g; 柴胡 10 g; 延胡索 10 g; 干姜 8 g; 砂仁 6 g; 白豆蔻 6 g; 甘草 6 g | *海螵蛸: Sepiae Endoconcha (Sepia spp.; animal-derived); 丹参: Salvia miltiorrhiza Bunge; 酸枣仁: Ziziphus jujuba Mill. var. spinosa (Bunge) Hu ex H.F.Chow; 龙骨: Fossilia Ossis Mastodi (fossil-derived); 牡蛎: Ostreae Concha (Ostrea spp.; animal-derived); 半夏: Pinellia ternata (Thunb.) Makino; 茯苓: Wolfiporia cocos (F.A.Wolf) Ryvarden & Gilb. (syn. Poria cocos); 葛根: Pueraria montana var. lobata (Willd.) Maesen & S.M.Almeida ex Sanjappa & Predeep; 黄芩: Scutellaria baicalensis Georgi; 党参: Codonopsis pilosula (Franch.) Nannf.; 桂枝: Cinnamomum cassia (L.) J.Presl; 白术: Atractylodes macrocephala Koidz.; 柴胡: Bupleurum chinense DC.; 延胡索: Corydalis yanhusuo W.T.Wang; 干姜: Zingiber officinale Roscoe; 砂仁: Wurfbainia villosa (Lour.) Skornick. & A.D.Poulsen (syn. Amomum villosum Lour.); 白豆蔻: Wurfbainia vera (Blackw.) Skornick. & A.D.Poulsen; 甘草: Glycyrrhiza uralensis Fisch. ex DC.* | None reported | 1 decoction/day; 150 mL per dose | Oral decoction | Three times daily | 8 weeks |
| S6 | You | 2021 | Chaipu decoction plus Huatan Xiaoyu formula | Modified/individualized | 陈皮 10 g; 法半夏 10 g; 猪苓 15 g; 莪术 10 g; 鸡内金 10 g; 丹参 15 g; 檀香 5 g; 砂仁 3 g; 吴茱萸 3 g; 黄连 3 g; 乌贼骨 20 g; 薏苡仁 30 g; 猫爪草 10 g; 仙鹤草 15 g; 柴胡 5 g; 黄芩 10 g; 党参 15 g; 甘草 3 g; 厚朴 10 g; 苏梗 10 g | *陈皮: Citrus reticulata Blanco; 半夏: Pinellia ternata (Thunb.) Makino; 猪苓: Polyporus umbellatus (Pers.) Fr.; 莪术: Curcuma spp. (species not specified); 鸡内金: Galli Gigerii Endothelium Corneum (animal-derived); 丹参: Salvia miltiorrhiza Bunge; 檀香: Santalum album L.; 砂仁: Wurfbainia villosa (Lour.) Skornick. & A.D.Poulsen (syn. Amomum villosum Lour.); 吴茱萸: Tetradium ruticarpum (A.Juss.) T.G.Hartley; 黄连: Coptis chinensis Franch.; 乌贼骨: Sepiae Endoconcha (Sepia spp.; animal-derived); 薏苡仁: Coix lacryma-jobi var. ma-yuen (Rom.Caill.) Stapf; 猫爪草: Ranunculus ternatus Thunb.; 仙鹤草: Agrimonia pilosa Ledeb.; 柴胡: Bupleurum chinense DC.; 黄芩: Scutellaria baicalensis Georgi; 党参: Codonopsis pilosula (Franch.) Nannf.; 甘草: Glycyrrhiza uralensis Fisch. ex DC.; 厚朴: Magnolia officinalis Rehder & E.H.Wilson; 苏梗: Perilla frutescens (L.) Britton* | Pain: 延胡索 10 g and 香附 10 g; reflux: 白及 10 g and 煅瓦楞子 20 g; distension/borborygmus: 防风 10 g and 木香 10 g; belching: 枳壳 10 g and 旋覆花 10 g; indigestion: 焦山楂 10 g and 焦神曲 10 g; insomnia: 酸枣仁 15 g and 夜交藤 20 g; red tongue: 麦冬 10 g and 生地黄 10 g; severe dysplasia: 藤梨根 20 g and 半枝莲 15 g | 1 decoction/day; concentrated decoction | Oral decoction after meals | Morning and evening | 6 months |
| S7 | Gao | 2022 | Fuwei Kangyi decoction | Fixed formula | 茯苓 20 g; 白花蛇舌草 20 g; 枳壳 20 g; 炒党参 15 g; 莪术 15 g; 陈皮 10 g; 半夏 10 g; 玄参 10 g; 石菖蒲 10 g; 鸡内金 10 g; 黄连 6 g; 甘草 6 g | *茯苓: Wolfiporia cocos (F.A.Wolf) Ryvarden & Gilb. (syn. Poria cocos); 白花蛇舌草: Scleromitrion diffusum (Willd.) R.J.Wang (syn. Hedyotis diffusa Willd.); 枳壳: Citrus aurantium L.; 党参: Codonopsis pilosula (Franch.) Nannf.; 莪术: Curcuma spp. (species not specified); 陈皮: Citrus reticulata Blanco; 半夏: Pinellia ternata (Thunb.) Makino; 玄参: Scrophularia ningpoensis Hemsl.; 石菖蒲: Acorus tatarinowii Schott; 鸡内金: Galli Gigerii Endothelium Corneum (animal-derived); 黄连: Coptis chinensis Franch.; 甘草: Glycyrrhiza uralensis Fisch. ex DC.* | None reported | 1 decoction/day; 150 mL per dose | Oral decoction | Twice daily | 3 months |
| S8 | Yi | 2022 | Fuwei Xiaowei decoction granules | Fixed formula | 党参 15 g; 茯苓 15 g; 蒲公英 15 g; 白术 12 g; 法半夏 9 g; 陈皮 10 g; 黄芩 10 g; 藿香 10 g; 木香 10 g; 砂仁 10 g; 半枝莲 10 g; 鸡内金 10 g; 灵芝 10 g; 黄连 5 g; 干姜 5 g; 甘草 5 g | *党参: Codonopsis pilosula (Franch.) Nannf.; 茯苓: Wolfiporia cocos (F.A.Wolf) Ryvarden & Gilb. (syn. Poria cocos); 蒲公英: Taraxacum mongolicum Hand.-Mazz.; 白术: Atractylodes macrocephala Koidz.; 半夏: Pinellia ternata (Thunb.) Makino; 陈皮: Citrus reticulata Blanco; 黄芩: Scutellaria baicalensis Georgi; 藿香: Pogostemon cablin (Blanco) Benth.; 木香: Aucklandia costus Falc.; 砂仁: Wurfbainia villosa (Lour.) Skornick. & A.D.Poulsen (syn. Amomum villosum Lour.); 半枝莲: Scutellaria barbata D.Don; 鸡内金: Galli Gigerii Endothelium Corneum (animal-derived); 灵芝: Ganoderma lingzhi Sheng H.Wu et al.; 黄连: Coptis chinensis Franch.; 干姜: Zingiber officinale Roscoe; 甘草: Glycyrrhiza uralensis Fisch. ex DC.* | None reported | 1 prescription/day | Oral granules after meals | Twice daily | 8 weeks |
| S9 | Zhang | 2016 | Modified Huatan Xiaoyu formula | Modified/individualized | 陈皮 12 g; 法半夏 10 g; 鸡内金 10 g; 紫丹参 15 g; 薏苡仁 30 g; 蒲黄粉 10 g; 半枝莲 15 g; 猫爪草 15 g; 仙鹤草 15 g | *陈皮: Citrus reticulata Blanco; 半夏: Pinellia ternata (Thunb.) Makino; 鸡内金: Galli Gigerii Endothelium Corneum (animal-derived); 丹参: Salvia miltiorrhiza Bunge; 薏苡仁: Coix lacryma-jobi var. ma-yuen (Rom.Caill.) Stapf; 蒲黄: Typha spp. (pollen); 半枝莲: Scutellaria barbata D.Don; 猫爪草: Ranunculus ternatus Thunb.; 仙鹤草: Agrimonia pilosa Ledeb.* | Liver–stomach disharmony: 柴胡, 黄连, 吴茱萸, 郁金, 旋覆花 and 香附; damp-heat: 藿香, 佩兰, 通草 and 车前子 | 1 decoction/day | Oral decoction after meals | Twice daily | 24 weeks |
| S10 | Zhang | 2020 | Modified Huatan Xiaoyu formula | Modified/individualized | 陈皮 10 g; 法半夏 10 g; 莪术 10 g; 鸡内金 10 g; 茯苓 15 g; 丹参 15 g; 半枝莲 15 g; 白花蛇舌草 15 g; 仙鹤草 15 g; 薏苡仁 30 g; 藤梨根 20 g | *陈皮: Citrus reticulata Blanco; 半夏: Pinellia ternata (Thunb.) Makino; 莪术: Curcuma spp. (species not specified); 鸡内金: Galli Gigerii Endothelium Corneum (animal-derived); 茯苓: Wolfiporia cocos (F.A.Wolf) Ryvarden & Gilb. (syn. Poria cocos); 丹参: Salvia miltiorrhiza Bunge; 半枝莲: Scutellaria barbata D.Don; 白花蛇舌草: Scleromitrion diffusum (Willd.) R.J.Wang (syn. Hedyotis diffusa Willd.); 仙鹤草: Agrimonia pilosa Ledeb.; 薏苡仁: Coix lacryma-jobi var. ma-yuen (Rom.Caill.) Stapf; 藤梨根: Actinidia chinensis Planch.* | Pain: 延胡索 10 g and 蒲黄粉 10 g; fullness: 檀香 5 g, 砂仁 3 g and 厚朴 10 g; reflux: 吴茱萸 3 g, 黄连 3 g, 乌贼骨 20 g and 瓦楞子 20 g; early satiety: 莱菔子 20 g and 神曲 10 g; belching: 旋覆花 10 g, 代赭石 30 g and 枇杷叶 10 g; yin deficiency: 北沙参 10 g and 麦冬 10 g | 1 decoction/day; 500 mL reduced to 300 mL | Oral decoction | Twice daily | 6 months |
| S11 | Huang | 2020 | Huangqi Jianzhong decoction plus Liangfu pill | Modified/individualized | 饴糖 30 g; 海螵蛸 20 g; 白芍 15 g; 黄芪 15 g; 桂枝 10 g; 高良姜 10 g; 香附 10 g; 炙甘草 10 g; 生姜 3 slices | *饴糖: Maltose (non-botanical); 海螵蛸: Sepiae Endoconcha (Sepia spp.; animal-derived); 白芍: Paeonia lactiflora Pall.; 黄芪: Astragalus mongholicus Bunge; 桂枝: Cinnamomum cassia (L.) J.Presl; 高良姜: Alpinia officinarum Hance; 香附: Cyperus rotundus L.; 甘草: Glycyrrhiza uralensis Fisch. ex DC.; 生姜: Zingiber officinale Roscoe* | Source reported syndrome-based additions; exact complete list not reliably recoverable | 1 decoction/day | Oral decoction | Twice daily | 4 weeks |
| S13 | Liu | 2005 | Guanxinning injection | Fixed formula | 丹参; 川芎 | *丹参: Salvia miltiorrhiza Bunge; 川芎: Conioselinum anthriscoides 'Chuanxiong' (syn. Ligusticum chuanxiong)* | None reported | 20 mL diluted in 250 mL 5% glucose-saline | Intravenous infusion | Once daily | 20 days |
| S14 | Liu | 2017 | Modified Fuzi Lizhong decoction | Fixed formula | 盐附子 15–51 g; 干姜 9 g; 红参 9 g; 麸炒白术 15 g; 炙甘草 3 g; 煨木香 15 g; 酒制白芍 15 g; 三七粉 3 g; 黄连 6 g | *附子: Aconitum carmichaelii Debeaux; 干姜: Zingiber officinale Roscoe; 红参: Panax ginseng C.A.Mey.; 白术: Atractylodes macrocephala Koidz.; 甘草: Glycyrrhiza uralensis Fisch. ex DC.; 木香: Aucklandia costus Falc.; 白芍: Paeonia lactiflora Pall.; 三七: Panax notoginseng (Burkill) F.H.Chen; 黄连: Coptis chinensis Franch.* | None reported | 1 decoction/day | Oral decoction after meals | Twice daily | 4 months |
| S15 | Wang | 2025 | Modified Sanren decoction | Modified/individualized | 薏苡仁 30 g; 豆蔻 10 g; 苦杏仁 10 g; 厚朴 12 g; 半夏 10 g; 车前草 15 g; 淡竹叶 12 g; 吴茱萸 3 g; 蒲公英 15 g; 砂仁 6 g; 黄芪 15 g; 莪术 10 g | *薏苡仁: Coix lacryma-jobi var. ma-yuen (Rom.Caill.) Stapf; 豆蔻: Wurfbainia vera (Blackw.) Skornick. & A.D.Poulsen; 苦杏仁: Prunus armeniaca L.; 厚朴: Magnolia officinalis Rehder & E.H.Wilson; 半夏: Pinellia ternata (Thunb.) Makino; 车前草: Plantago asiatica L.; 淡竹叶: Lophatherum gracile Brongn.; 吴茱萸: Tetradium ruticarpum (A.Juss.) T.G.Hartley; 蒲公英: Taraxacum mongolicum Hand.-Mazz.; 砂仁: Wurfbainia villosa (Lour.) Skornick. & A.D.Poulsen (syn. Amomum villosum Lour.); 黄芪: Astragalus mongholicus Bunge; 莪术: Curcuma spp. (species not specified)* | Pain: 延胡索 10 g; frequent acid regurgitation: 海螵蛸 15 g; distension: 枳实 10 g; indigestion: 神曲 10 g; dry mouth: 石斛 10 g; constipation: 火麻仁 15 g; irritability: 柴胡 10 g; insomnia: 酸枣仁 15 g | 1 decoction/day; 400 mL total | Oral decoction | Twice daily | 10 days |
| S16 | Zhuang | 2011 | Jianpi Huatan formula | Modified/individualized | 半夏 15 g; 党参 15 g; 白术 10 g; 厚朴 15 g; 柴胡 10 g; 浙贝母 15 g; 瓜蒌 15 g; 六神曲 10 g | *半夏: Pinellia ternata (Thunb.) Makino; 党参: Codonopsis pilosula (Franch.) Nannf.; 白术: Atractylodes macrocephala Koidz.; 厚朴: Magnolia officinalis Rehder & E.H.Wilson; 柴胡: Bupleurum chinense DC.; 浙贝母: Fritillaria thunbergii Miq.; 瓜蒌: Trichosanthes kirilowii Maxim.; 神曲: Massa Medicata Fermentata (fermented product)* | Add 黄连 10 g, 干姜 10 g or 杏仁 10 g as indicated; 厚朴 increased to 30 g for marked fullness | 1 decoction/day | Oral decoction | Twice daily | 3 months (4-week courses with 2-day intervals) |
| S17 | Wang | 2021 | Jianpi Huatan Chinese herbal medicine | Modified/individualized | 党参 15 g; 半夏 15 g; 厚朴 15 g; 浙贝母 15 g; 瓜蒌 15 g; 白术 10 g; 柴胡 10 g; 六神曲 10 g | *党参: Codonopsis pilosula (Franch.) Nannf.; 半夏: Pinellia ternata (Thunb.) Makino; 厚朴: Magnolia officinalis Rehder & E.H.Wilson; 浙贝母: Fritillaria thunbergii Miq.; 瓜蒌: Trichosanthes kirilowii Maxim.; 白术: Atractylodes macrocephala Koidz.; 柴胡: Bupleurum chinense DC.; 神曲: Massa Medicata Fermentata (fermented product)* | Add 黄连 10 g, 杏仁 10 g or 干姜 10 g as indicated; 厚朴 increased to 30 g for marked fullness | 1 decoction/day | Oral decoction | Twice daily | 3 months (three 4-week courses with 2-day intervals) |
| S18 | Zhang | 2013 | Jianpi Huoxue formula | Fixed formula | 炙黄芪 15 g; 党参 15 g; 枳壳 15 g; 八月札 15 g; 白术 12 g; 茯苓 15 g; 当归 12 g; 莪术 12 g | *黄芪: Astragalus mongholicus Bunge; 党参: Codonopsis pilosula (Franch.) Nannf.; 枳壳: Citrus aurantium L.; 八月札: Akebia trifoliata (Thunb.) Koidz.; 白术: Atractylodes macrocephala Koidz.; 茯苓: Wolfiporia cocos (F.A.Wolf) Ryvarden & Gilb. (syn. Poria cocos); 当归: Angelica sinensis (Oliv.) Diels; 莪术: Curcuma spp. (species not specified)* | None reported | 1 decoction/day; 150 mL per dose | Oral decoction after meals | Twice daily | 8 weeks |
| S19 | Shen | 2021 | Jianpi Huoxue Xiaoji formula | Fixed formula | 黄芪 30 g; 党参 20 g; 木香 20 g; 白术 20 g; 柴胡 20 g; 枳实 20 g; 当归 15 g; 陈皮 15 g; 丹参 15 g; 白芍 15 g; 法半夏 10 g; 甘草 6 g | *黄芪: Astragalus mongholicus Bunge; 党参: Codonopsis pilosula (Franch.) Nannf.; 木香: Aucklandia costus Falc.; 白术: Atractylodes macrocephala Koidz.; 柴胡: Bupleurum chinense DC.; 枳实: Citrus aurantium L.; 当归: Angelica sinensis (Oliv.) Diels; 陈皮: Citrus reticulata Blanco; 丹参: Salvia miltiorrhiza Bunge; 白芍: Paeonia lactiflora Pall.; 半夏: Pinellia ternata (Thunb.) Makino; 甘草: Glycyrrhiza uralensis Fisch. ex DC.* | None reported | 1 decoction/day | Oral decoction | Twice daily | 8 weeks |
| S20 | Wang | 2023 | Jianpi Liqi Huayu formula | Fixed formula | 丹参 12 g; 党参 12 g; 炒白术 10 g; 生黄芪 15 g; 厚朴 10 g; 枳壳 10 g; 莪术 10 g; 三棱 10 g; 延胡索 6 g; 乌药 6 g | *丹参: Salvia miltiorrhiza Bunge; 党参: Codonopsis pilosula (Franch.) Nannf.; 白术: Atractylodes macrocephala Koidz.; 黄芪: Astragalus mongholicus Bunge; 厚朴: Magnolia officinalis Rehder & E.H.Wilson; 枳壳: Citrus aurantium L.; 莪术: Curcuma spp. (species not specified); 三棱: Sparganium stoloniferum (Buch.-Ham. ex Graebn.) Buch.-Ham. ex Juz.; 延胡索: Corydalis yanhusuo W.T.Wang; 乌药: Lindera aggregata (Sims) Kosterm.* | None reported | 1 decoction/day; 500 mL reduced to 200 mL | Oral decoction | Twice daily | 12 weeks |
| S21 | Zhang | 2025 | Jianpi Tongluo prescription | Fixed formula | 炙黄芪 30 g; 炒白术 15 g; 党参 15 g; 炒薏苡仁 15 g; 赤芍 15 g; 蒲公英 15 g; 白花蛇舌草 15 g; 丹参 15 g; 陈皮 10 g; 柴胡 10 g; 升麻 10 g; 炙甘草 6 g | *黄芪: Astragalus mongholicus Bunge; 白术: Atractylodes macrocephala Koidz.; 党参: Codonopsis pilosula (Franch.) Nannf.; 薏苡仁: Coix lacryma-jobi var. ma-yuen (Rom.Caill.) Stapf; 赤芍: Paeonia lactiflora Pall. / Paeonia veitchii Lynch; 蒲公英: Taraxacum mongolicum Hand.-Mazz.; 白花蛇舌草: Scleromitrion diffusum (Willd.) R.J.Wang (syn. Hedyotis diffusa Willd.); 丹参: Salvia miltiorrhiza Bunge; 陈皮: Citrus reticulata Blanco; 柴胡: Bupleurum chinense DC.; 升麻: Actaea heracleifolia (Kom.) J.Compton; 甘草: Glycyrrhiza uralensis Fisch. ex DC.* | None reported | 1 decoction/day; 300 mL | Oral decoction | Twice daily | 8 weeks |
| S22 | Jia | 2021 | Jianpi Yiqi decoction | Modified/individualized | 黄芪 30 g; 茯苓 20 g; 白芍 20 g; 当归尾 20 g; 党参 15 g; 白术 15 g; 薏苡仁 10 g; 升麻 10 g; 柴胡 10 g; 半夏 10 g; 木香 10 g; 白花蛇舌草 10 g; 甘草 6 g; 草豆蔻 6 g | *黄芪: Astragalus mongholicus Bunge; 茯苓: Wolfiporia cocos (F.A.Wolf) Ryvarden & Gilb. (syn. Poria cocos); 白芍: Paeonia lactiflora Pall.; 当归: Angelica sinensis (Oliv.) Diels; 党参: Codonopsis pilosula (Franch.) Nannf.; 白术: Atractylodes macrocephala Koidz.; 薏苡仁: Coix lacryma-jobi var. ma-yuen (Rom.Caill.) Stapf; 升麻: Actaea heracleifolia (Kom.) J.Compton; 柴胡: Bupleurum chinense DC.; 半夏: Pinellia ternata (Thunb.) Makino; 木香: Aucklandia costus Falc.; 白花蛇舌草: Scleromitrion diffusum (Willd.) R.J.Wang (syn. Hedyotis diffusa Willd.); 甘草: Glycyrrhiza uralensis Fisch. ex DC.; 草豆蔻: Alpinia katsumadai Hayata* | Distension: 佛手 10 g; loose stool: 炒扁豆 10 g; poor appetite: 鸡内金 10 g | 1 decoction/day; 300 mL in 2 bags | Oral decoction | Twice daily | 12 weeks |
| S24 | Yang | 2024 | Liqi Huayu Jianwei decoction | Fixed formula | 薏苡仁 20 g; 山药 15 g; 炒白术 15 g; 黄芪 15 g; 党参 15 g; 山楂 10 g; 厚朴 10 g; 郁金 10 g; 枳壳 10 g; 陈皮 6 g; 砂仁 6 g; 丹参 6 g; 甘草 6 g; 三七粉 3 g | *薏苡仁: Coix lacryma-jobi var. ma-yuen (Rom.Caill.) Stapf; 山药: Dioscorea polystachya Turcz.; 白术: Atractylodes macrocephala Koidz.; 黄芪: Astragalus mongholicus Bunge; 党参: Codonopsis pilosula (Franch.) Nannf.; 山楂: Crataegus pinnatifida Bunge; 厚朴: Magnolia officinalis Rehder & E.H.Wilson; 郁金: Curcuma spp. (species not specified); 枳壳: Citrus aurantium L.; 陈皮: Citrus reticulata Blanco; 砂仁: Wurfbainia villosa (Lour.) Skornick. & A.D.Poulsen (syn. Amomum villosum Lour.); 丹参: Salvia miltiorrhiza Bunge; 甘草: Glycyrrhiza uralensis Fisch. ex DC.; 三七: Panax notoginseng (Burkill) F.H.Chen* | None reported | 1 decoction/day; 300 mL | Oral decoction | Twice daily | 8 weeks |
| S26 | Wang | 2018 | Liuwei Anxiao capsules | Proprietary product; source-reported ingredients | 土木香; 大黄; 山柰; 诃子; 寒水石; 碱花 | *土木香: Inula helenium L.; 大黄: Rheum palmatum L.; 山柰: Kaempferia galanga L.; 诃子: Terminalia chebula Retz.; 寒水石: Calcitum (mineral-derived); 碱花: Natrii Carbonas (mineral salt)* | None reported | 3 capsules per dose (0.5 g/capsule) | Oral capsules after meals | Three times daily | 4 weeks |
| S27 | Ning | 2016 | Manwei San | Fixed formula | 黄芪 30 g; 党参 30 g; 太子参 30 g; 山药 30 g; 蒲公英 30 g; 焦白术 30 g; 乌梅 20 g; 鸡内金 20 g; 炒山楂 20 g; 黄连 8 g; 甘草 10 g; 白芍 9 g; 神曲 10 g; 香橼 15 g; 莪术 10 g | *黄芪: Astragalus mongholicus Bunge; 党参: Codonopsis pilosula (Franch.) Nannf.; 太子参: Pseudostellaria heterophylla (Miq.) Pax; 山药: Dioscorea polystachya Turcz.; 蒲公英: Taraxacum mongolicum Hand.-Mazz.; 白术: Atractylodes macrocephala Koidz.; 乌梅: Prunus mume Siebold & Zucc.; 鸡内金: Galli Gigerii Endothelium Corneum (animal-derived); 山楂: Crataegus pinnatifida Bunge; 黄连: Coptis chinensis Franch.; 甘草: Glycyrrhiza uralensis Fisch. ex DC.; 白芍: Paeonia lactiflora Pall.; 神曲: Massa Medicata Fermentata (fermented product); 香橼: Citrus medica L.; 莪术: Curcuma spp. (species not specified)* | None reported | 9 g per dose | Oral powder | Three times daily | 3 months |
| S28 | Qi | 2024 | Self-made Chenpi formula | Fixed formula | 陈皮 20 g; 黄芪 20 g; 太子参 15 g; 白术 20 g; 茯苓 15 g; 香附 10 g; 肉豆蔻 10 g; 白芍 10 g; 当归 10 g; 丹参 10 g; 刘寄奴 10 g; 枳壳 20 g; 炙甘草 6 g | *陈皮: Citrus reticulata Blanco; 黄芪: Astragalus mongholicus Bunge; 太子参: Pseudostellaria heterophylla (Miq.) Pax; 白术: Atractylodes macrocephala Koidz.; 茯苓: Wolfiporia cocos (F.A.Wolf) Ryvarden & Gilb. (syn. Poria cocos); 香附: Cyperus rotundus L.; 肉豆蔻: Myristica fragrans Houtt.; 白芍: Paeonia lactiflora Pall.; 当归: Angelica sinensis (Oliv.) Diels; 丹参: Salvia miltiorrhiza Bunge; 刘寄奴: Artemisia anomala S.Moore; 枳壳: Citrus aurantium L.; 甘草: Glycyrrhiza uralensis Fisch. ex DC.* | None reported | 1 decoction/day; 1000 mL reduced to 150 mL | Oral decoction before meals | Three times daily | 3 months |
| S29 | Dong | 2024 | Piwei Peiyuan decoction | Fixed formula | 炙黄芪 18 g; 白术 20 g; 白芍 10 g; 石斛 15 g; 香附 10 g; 刘寄奴 6 g | *黄芪: Astragalus mongholicus Bunge; 白术: Atractylodes macrocephala Koidz.; 白芍: Paeonia lactiflora Pall.; 石斛: Dendrobium spp. (species not specified); 香附: Cyperus rotundus L.; 刘寄奴: Artemisia anomala S.Moore* | None reported | 1 decoction/day; 400 mL | Oral decoction | Twice daily | 18 weeks |
| S30 | Yang | 2017 | Shugan Hewei Tang | Modified/individualized | 法半夏 15 g; 枳壳 15 g; 厚朴 12 g; 柴胡 12 g; 党参 15 g; 炒白术 15 g; 茯苓 15 g; 炙甘草 6 g; 丹参 15 g; 仙鹤草 15 g | *半夏: Pinellia ternata (Thunb.) Makino; 枳壳: Citrus aurantium L.; 厚朴: Magnolia officinalis Rehder & E.H.Wilson; 柴胡: Bupleurum chinense DC.; 党参: Codonopsis pilosula (Franch.) Nannf.; 白术: Atractylodes macrocephala Koidz.; 茯苓: Wolfiporia cocos (F.A.Wolf) Ryvarden & Gilb. (syn. Poria cocos); 甘草: Glycyrrhiza uralensis Fisch. ex DC.; 丹参: Salvia miltiorrhiza Bunge; 仙鹤草: Agrimonia pilosa Ledeb.* | Add 高良姜 9 g and 香附 6 g; or 鸡内金 15 g and 焦山楂 15 g; or 海螵蛸 15 g and 浙贝母 15 g; or 薏苡仁 20 g according to symptoms | 1 decoction/day; 200 mL | Oral decoction | Twice daily | 2 months |
| S31 | Xu | 2018 | Weifuchun tablets | Proprietary product; source-reported ingredients | 香茶菜; 红参; 枳壳 | *香茶菜: Isodon amethystoides (Benth.) H.Hara; 红参: Panax ginseng C.A.Mey.; 枳壳: Citrus aurantium L.* | None reported | 4 tablets per dose (0.36 g/tablet) | Oral tablets | Three times daily | 2 months |
| S32 | Liu | 2010 | Weishu decoction | Fixed formula | 茯苓; 白芍; 柴胡; 苍术; 川楝子; 延胡索; 甘草; 枳壳; 谷芽; 熟大黄; 丹参; 蒲公英 | *茯苓: Wolfiporia cocos (F.A.Wolf) Ryvarden & Gilb. (syn. Poria cocos); 白芍: Paeonia lactiflora Pall.; 柴胡: Bupleurum chinense DC.; 苍术: Atractylodes lancea (Thunb.) DC.; 川楝子: Melia toosendan Siebold & Zucc.; 延胡索: Corydalis yanhusuo W.T.Wang; 甘草: Glycyrrhiza uralensis Fisch. ex DC.; 枳壳: Citrus aurantium L.; 谷芽: Oryza sativa L.; 熟大黄: Rheum palmatum L.; 丹参: Salvia miltiorrhiza Bunge; 蒲公英: Taraxacum mongolicum Hand.-Mazz.* | None reported | 1 decoction/day; 200 mL per dose | Oral decoction | Twice daily | 6 months |
| S33 | Xing | 2004 | Weite'an tablets | Proprietary hospital preparation; source-reported ingredients | 白术 45 g; 枳实 25 g; 苍术 15 g; 茯苓 35 g; 神曲 30 g; 半夏 30 g; 泽泻 30 g; 黄芪 30 g; 川芎 15 g; 海螵蛸 15 g; 草豆蔻 10 g; 黄芩 10 g; 莱菔子 10 g; 干姜 10 g; 陈皮 25 g; 制香附 15 g; 瓜蒌 15 g; 槟榔 15 g; 厚朴 10 g; 黄柏 15 g; 木香 10 g; 马兰 20 g | *白术: Atractylodes macrocephala Koidz.; 枳实: Citrus aurantium L.; 苍术: Atractylodes lancea (Thunb.) DC.; 茯苓: Wolfiporia cocos (F.A.Wolf) Ryvarden & Gilb. (syn. Poria cocos); 神曲: Massa Medicata Fermentata (fermented product); 半夏: Pinellia ternata (Thunb.) Makino; 泽泻: Alisma plantago-aquatica subsp. orientale (Sam.) Sam.; 黄芪: Astragalus mongholicus Bunge; 川芎: Conioselinum anthriscoides 'Chuanxiong' (syn. Ligusticum chuanxiong); 海螵蛸: Sepiae Endoconcha (Sepia spp.; animal-derived); 草豆蔻: Alpinia katsumadai Hayata; 黄芩: Scutellaria baicalensis Georgi; 莱菔子: Raphanus sativus L.; 干姜: Zingiber officinale Roscoe; 陈皮: Citrus reticulata Blanco; 香附: Cyperus rotundus L.; 瓜蒌: Trichosanthes kirilowii Maxim.; 槟榔: Areca catechu L.; 厚朴: Magnolia officinalis Rehder & E.H.Wilson; 黄柏: Phellodendron chinense C.K.Schneid.; 木香: Aucklandia costus Falc.; 马兰: Kalimeris indica (L.) Sch.Bip.* | None reported | 5 tablets per dose | Oral tablets | Three times daily | 3 months |
| S34 | Jiang | 2025 | Wenyang Jianpi ointment | Fixed formula | 桂枝 15 g; 白芍 30 g; 饴糖 30 g; 黄芪 30 g; 党参 30 g; 茯苓 30 g; 白术 30 g; 甘草 15 g; 干姜 15 g; 乌药 20 g; 陈皮 12 g; 升麻 12 g; 三棱 30 g; 莪术 30 g | *桂枝: Cinnamomum cassia (L.) J.Presl; 白芍: Paeonia lactiflora Pall.; 饴糖: Maltose (non-botanical); 黄芪: Astragalus mongholicus Bunge; 党参: Codonopsis pilosula (Franch.) Nannf.; 茯苓: Wolfiporia cocos (F.A.Wolf) Ryvarden & Gilb. (syn. Poria cocos); 白术: Atractylodes macrocephala Koidz.; 甘草: Glycyrrhiza uralensis Fisch. ex DC.; 干姜: Zingiber officinale Roscoe; 乌药: Lindera aggregata (Sims) Kosterm.; 陈皮: Citrus reticulata Blanco; 升麻: Actaea heracleifolia (Kom.) J.Compton; 三棱: Sparganium stoloniferum (Buch.-Ham. ex Graebn.) Buch.-Ham. ex Juz.; 莪术: Curcuma spp. (species not specified)* | None reported | 20 g per dose (paste prepared to about 140 g/bowl) | Oral paste | Once each morning | 12 weeks |
| S35 | Zhang | 2021 | Wenyang Jianpi decoction | Modified/individualized | 丹参 20 g; 白芍 20 g; 当归尾 20 g; 茯苓 20 g; 白术 15 g; 党参 15 g; 淫羊藿 12 g; 柴胡 10 g; 黄柏 10 g; 知母 10 g; 莪术 9 g; 甘草 9 g; 法半夏 9 g; 仙茅 6 g; 砂仁 6 g; 甘松 6 g; 蛇床子 6 g | *丹参: Salvia miltiorrhiza Bunge; 白芍: Paeonia lactiflora Pall.; 当归: Angelica sinensis (Oliv.) Diels; 茯苓: Wolfiporia cocos (F.A.Wolf) Ryvarden & Gilb. (syn. Poria cocos); 白术: Atractylodes macrocephala Koidz.; 党参: Codonopsis pilosula (Franch.) Nannf.; 淫羊藿: Epimedium spp. (species not specified); 柴胡: Bupleurum chinense DC.; 黄柏: Phellodendron chinense C.K.Schneid.; 知母: Anemarrhena asphodeloides Bunge; 莪术: Curcuma spp. (species not specified); 甘草: Glycyrrhiza uralensis Fisch. ex DC.; 半夏: Pinellia ternata (Thunb.) Makino; 仙茅: Curculigo orchioides Gaertn.; 砂仁: Wurfbainia villosa (Lour.) Skornick. & A.D.Poulsen (syn. Amomum villosum Lour.); 甘松: Nardostachys jatamansi (D.Don) DC.; 蛇床子: Cnidium monnieri (L.) Cusson* | Distension: 莱菔子 15 g, 厚朴 10 g and 枳实 10 g; belching: 旋覆花 15 g and 代赭石 15 g | 250 mL; 1 decoction every 2 days | Oral decoction | Every 2 days | 2 months |
| S37 | Bu | 2019 | Xiaohua Funing decoction | Fixed formula | 谷芽 30 g; 绿梅花 20 g; 枳壳 15 g; 姜半夏 12 g; 姜竹茹 10 g; 柴胡 10 g | *谷芽: Oryza sativa L.; 绿梅花: Prunus mume Siebold & Zucc.; 枳壳: Citrus aurantium L.; 半夏: Pinellia ternata (Thunb.) Makino; 竹茹: Bambusae Caulis in Taenias (Bambusa spp.); 柴胡: Bupleurum chinense DC.* | None reported | 1 decoction/day; 400 mL | Oral decoction after meals | Twice daily | 3 months |
| S38 | Yu | 2023 | Yiqi Huoxue prescription | Modified/individualized | 炒白术 20 g; 炒党参 20 g; 枳壳 15 g; 鸡内金 15 g; 桃仁 15 g; 半枝莲 15 g; 薏苡仁 15 g; 白芍 30 g; 白茯苓 30 g; 五灵脂 10 g; 丹参 10 g; 木香 10 g; 蒲黄 10 g; 姜半夏 10 g; 荷叶 10 g; 甘草 8 g; 红花 5 g | *白术: Atractylodes macrocephala Koidz.; 党参: Codonopsis pilosula (Franch.) Nannf.; 枳壳: Citrus aurantium L.; 鸡内金: Galli Gigerii Endothelium Corneum (animal-derived); 桃仁: Prunus persica (L.) Batsch; 半枝莲: Scutellaria barbata D.Don; 薏苡仁: Coix lacryma-jobi var. ma-yuen (Rom.Caill.) Stapf; 白芍: Paeonia lactiflora Pall.; 白茯苓: Wolfiporia cocos (F.A.Wolf) Ryvarden & Gilb.; 五灵脂: Trogopterus xanthipes Milne-Edwards (animal-derived); 丹参: Salvia miltiorrhiza Bunge; 木香: Aucklandia costus Falc.; 蒲黄: Typha spp. (pollen); 半夏: Pinellia ternata (Thunb.) Makino; 荷叶: Nelumbo nucifera Gaertn.; 甘草: Glycyrrhiza uralensis Fisch. ex DC.; 红花: Carthamus tinctorius L.* | Reflux: 海螵蛸 20 g; intestinal metaplasia: 白花蛇舌草 10 g | 1 decoction/day; 200 mL | Oral decoction on an empty stomach | Twice daily | 1 month |
| S39 | Xu | 2021 | Yiwei Huazhuo Jiedu decoction | Modified/individualized | 北沙参 12 g; 麦冬 12 g; 生地 20 g; 石菖蒲 12 g; 当归 12 g; 丹参 15 g; 香橼 9 g; 佛手 9 g; 鸡内金 12 g; 甘草 6 g; 白花蛇舌草 20 g | *北沙参: Glehnia littoralis F.Schmidt ex Miq.; 麦冬: Ophiopogon japonicus (Thunb.) Ker Gawl.; 生地: Rehmannia glutinosa (Gaertn.) DC.; 石菖蒲: Acorus tatarinowii Schott; 当归: Angelica sinensis (Oliv.) Diels; 丹参: Salvia miltiorrhiza Bunge; 香橼: Citrus medica L.; 佛手: Citrus medica var. sarcodactylis (Noot.) Swingle; 鸡内金: Galli Gigerii Endothelium Corneum (animal-derived); 甘草: Glycyrrhiza uralensis Fisch. ex DC.; 白花蛇舌草: Scleromitrion diffusum (Willd.) R.J.Wang (syn. Hedyotis diffusa Willd.)* | Acid regurgitation/burning: 海螵蛸 15 g and 牡蛎 12 g; distension/pain: 厚朴 12 g and 枳壳 12 g; dry constipation: 火麻仁 15 g and 生白术 30 g | 1 decoction/day; 300 mL | Oral decoction after meals | Twice daily | 3 months |
| S40 | Li | 2019 | Yiwei Jianpi decoction | Fixed formula | 炙甘草 6 g; 山楂 10 g; 莪术 10 g; 桂枝 10 g; 丹参 15 g; 白术 15 g; 白芍 15 g; 赤芍 15 g; 党参 20 g; 黄芪 20 g | *甘草: Glycyrrhiza uralensis Fisch. ex DC.; 山楂: Crataegus pinnatifida Bunge; 莪术: Curcuma spp. (species not specified); 桂枝: Cinnamomum cassia (L.) J.Presl; 丹参: Salvia miltiorrhiza Bunge; 白术: Atractylodes macrocephala Koidz.; 白芍: Paeonia lactiflora Pall.; 赤芍: Paeonia lactiflora Pall. / Paeonia veitchii Lynch; 党参: Codonopsis pilosula (Franch.) Nannf.; 黄芪: Astragalus mongholicus Bunge* | None reported | 1 decoction/day; 300 mL | Oral decoction | Morning and evening | 8 weeks |
| S42 | Sun | 2017 | Curcuma granules or Notoginseng powder (two intervention arms) | Two fixed single-herb intervention arms | 郁金; 三七 | *郁金: Curcuma spp. (species not specified); 三七: Panax notoginseng (Burkill) F.H.Chen* | Both intervention arms also received folic acid 5 mg three times daily | Curcuma: 1 g per dose; Notoginseng: 1.5 g per dose | Oral granules/powder | Twice daily | 6 months |
| S43 | Wang | 2010 | Zhenqi Fuzheng capsules | Proprietary product; source-reported ingredients | 黄芪; 女贞子 | *黄芪: Astragalus mongholicus Bunge; 女贞子: Ligustrum lucidum W.T.Aiton* | None reported | 2 capsules per dose (0.35 g/capsule) | Oral capsules | Twice daily | 6 months |
| S44 | Zhang | 2008 | Danshen Yin plus Chaishao Liujunzi decoction | Modified/individualized | 黄芪 30 g; 人参 6 g; 茯苓 12 g; 白术 6 g; 丹参 30 g; 柴胡 10 g; 黄芩 12 g; 甘草 3 g | *黄芪: Astragalus mongholicus Bunge; 人参: Panax ginseng C.A.Mey.; 茯苓: Wolfiporia cocos (F.A.Wolf) Ryvarden & Gilb. (syn. Poria cocos); 白术: Atractylodes macrocephala Koidz.; 丹参: Salvia miltiorrhiza Bunge; 柴胡: Bupleurum chinense DC.; 黄芩: Scutellaria baicalensis Georgi; 甘草: Glycyrrhiza uralensis Fisch. ex DC.* | Hp infection: 蒲公英, 连翘, 半枝莲 and 黄连; congestion/stasis: 川芎, 延胡索 and 三七; bleeding: 仙鹤草 and 地榆; low acid: 木瓜, 乌梅, 山楂 and 玄参; intestinal metaplasia: 薏苡仁, 白花蛇舌草 and 败酱草; dysplasia: 莪术, 仙鹤草, 旱莲草, 水红花子, 半枝莲, 灵芝 and 生薏苡仁; pain: 延胡索 and 木香; excess acid: 乌贼骨 and 瓦楞子 | 1 decoction/day | Oral decoction | Twice daily | 2–5 courses (30 days/course) |
| S45 | Zhang | 2004 | Yangwei decoction | Fixed formula | 黄芪 18 g; 白术 12 g; 党参 10 g; 黄连 12 g; 吴茱萸 10 g; 蒲公英 15 g; 枳壳 12 g; 砂仁 10 g; 白芷 10 g; 莪术 10 g; 丹参 12 g; 甘草 10 g | *黄芪: Astragalus mongholicus Bunge; 白术: Atractylodes macrocephala Koidz.; 党参: Codonopsis pilosula (Franch.) Nannf.; 黄连: Coptis chinensis Franch.; 吴茱萸: Tetradium ruticarpum (A.Juss.) T.G.Hartley; 蒲公英: Taraxacum mongolicum Hand.-Mazz.; 枳壳: Citrus aurantium L.; 砂仁: Wurfbainia villosa (Lour.) Skornick. & A.D.Poulsen (syn. Amomum villosum Lour.); 白芷: Angelica dahurica (Hoffm.) Benth. & Hook.f. ex Franch. & Sav.; 莪术: Curcuma spp. (species not specified); 丹参: Salvia miltiorrhiza Bunge; 甘草: Glycyrrhiza uralensis Fisch. ex DC.* | None reported | 1 decoction/day; 600 mL reduced to 300 mL | Oral decoction | Twice daily; 6 days/week | 8 weeks |
| S46 | Fu | 2020 | Jianpi Tongluo decoction | Modified/individualized | 党参 30 g; 丹参 20 g; 茯苓 20 g; 炒白芍 15 g; 干姜 15 g; 炒枳壳 15 g; 大枣 10 g; 炙甘草 6 g; 黄连 5 g | *党参: Codonopsis pilosula (Franch.) Nannf.; 丹参: Salvia miltiorrhiza Bunge; 茯苓: Wolfiporia cocos (F.A.Wolf) Ryvarden & Gilb. (syn. Poria cocos); 白芍: Paeonia lactiflora Pall.; 干姜: Zingiber officinale Roscoe; 枳壳: Citrus aurantium L.; 大枣: Ziziphus jujuba Mill.; 甘草: Glycyrrhiza uralensis Fisch. ex DC.; 黄连: Coptis chinensis Franch.* | Belching: 生赭石 20 g and 旋覆花 15 g; dry mouth: 天冬 20 g and 乌梅 15 g; poor appetite: 鸡内金 15 g and 山楂 15 g; distension: 佛手 15 g and 香橼 10 g | 1 decoction/day; 300 mL | Oral decoction 30 min after meals | Morning and evening | 6 months |
| S47 | Wang | 2003 | Modified Xiangsha Liujunzi decoction | Modified/individualized | 香附 15 g; 砂仁 10 g; 人参 15 g; 白术 20 g; 茯苓 15 g; 甘草 10 g; 陈皮 10 g; 制半夏 10 g | *香附: Cyperus rotundus L.; 砂仁: Wurfbainia villosa (Lour.) Skornick. & A.D.Poulsen (syn. Amomum villosum Lour.); 人参: Panax ginseng C.A.Mey.; 白术: Atractylodes macrocephala Koidz.; 茯苓: Wolfiporia cocos (F.A.Wolf) Ryvarden & Gilb. (syn. Poria cocos); 甘草: Glycyrrhiza uralensis Fisch. ex DC.; 陈皮: Citrus reticulata Blanco; 半夏: Pinellia ternata (Thunb.) Makino* | Marked qi deficiency: 黄芪 30 g; gastric pain: Liangfu pill permitted | 1 decoction/day; 1000 mL reduced to 600 mL; 300 mL per dose | Oral decoction | Twice daily | 6 weeks |
| S48 | Sun | 2020 | Modified Yiwei decoction | Modified/individualized | 党参 15 g; 炒扁豆 15 g; 丹参 10 g; 麦冬 10 g; 白芍 10 g; 沙参 10 g; 当归 10 g; 龙葵 9 g; 半枝莲 9 g; 甘草 9 g; 枳壳 9 g; 姜半夏 9 g; 黄连 5 g | *党参: Codonopsis pilosula (Franch.) Nannf.; 白扁豆: Lablab purpureus (L.) Sweet; 丹参: Salvia miltiorrhiza Bunge; 麦冬: Ophiopogon japonicus (Thunb.) Ker Gawl.; 白芍: Paeonia lactiflora Pall.; 沙参: Adenophora spp. (species not specified); 当归: Angelica sinensis (Oliv.) Diels; 龙葵: Solanum nigrum L.; 半枝莲: Scutellaria barbata D.Don; 甘草: Glycyrrhiza uralensis Fisch. ex DC.; 枳壳: Citrus aurantium L.; 半夏: Pinellia ternata (Thunb.) Makino; 黄连: Coptis chinensis Franch.* | Bile reflux: 柴胡 8 g, 郁金 8 g and 竹茹 8 g; belching: 代赭石 20 g and 旋覆花 12 g; duodenal ulcer: 海螵蛸 10 g and 白芷 10 g; intestinal metaplasia: 莪术 8 g, 水蛭 10 g, 白花蛇舌草 30 g and 浙贝母 8 g | 1 decoction/day | Oral decoction 30 min before breakfast and dinner | Twice daily | 3 months |
| S49 | Wu | 2017 | Modified Zisheng decoction | Modified/individualized | 山药 30 g; 白术 20 g; 苍术 10 g; 黄连 15 g; 鸡内金 15 g; 石斛 10 g; 陈皮 8 g; 牛蒡子 8 g | *山药: Dioscorea polystachya Turcz.; 白术: Atractylodes macrocephala Koidz.; 苍术: Atractylodes lancea (Thunb.) DC.; 黄连: Coptis chinensis Franch.; 鸡内金: Galli Gigerii Endothelium Corneum (animal-derived); 石斛: Dendrobium spp. (species not specified); 陈皮: Citrus reticulata Blanco; 牛蒡子: Arctium lappa L.* | Pain: 郁金 10 g and 白术 increased to 30 g; distension: 木香 15 g and 陈皮 increased to 20 g; belching/acid: 乌贼骨 15 g and 旋覆花 10 g; insomnia: 合欢皮 10 g and 酸枣仁 10 g; distension/poor appetite: 厚朴 8 g and 苍术 increased to 20 g; severe intestinal metaplasia: 白花蛇舌草 15 g and 半枝莲 10 g | 150 mL per bag | Oral decoction after meals | Twice daily | 30 days |
| S50 | Lin | 2023 | Self-formulated Baidan Weiwei decoction | Fixed formula | 丹参 30 g; 百合 15 g; 乌药 10 g; 木香 10 g; 高良姜 6 g; 砂仁 6 g; 预知子 10 g; 胡黄连 10 g; 党参 15 g; 薏苡仁 15 g; 白术 15 g; 鸡内金 10 g | *丹参: Salvia miltiorrhiza Bunge; 百合: Lilium brownii var. viridulum Baker; 乌药: Lindera aggregata (Sims) Kosterm.; 木香: Aucklandia costus Falc.; 高良姜: Alpinia officinarum Hance; 砂仁: Wurfbainia villosa (Lour.) Skornick. & A.D.Poulsen (syn. Amomum villosum Lour.); 预知子: Akebia spp. (species not specified); 胡黄连: Picrorhiza scrophulariiflora Pennell; 党参: Codonopsis pilosula (Franch.) Nannf.; 薏苡仁: Coix lacryma-jobi var. ma-yuen (Rom.Caill.) Stapf; 白术: Atractylodes macrocephala Koidz.; 鸡内金: Galli Gigerii Endothelium Corneum (animal-derived)* | None reported | 1 decoction/day; 300 mL | Oral decoction 30 min after meals | Morning and evening | 3 months |
| S51 | Wang | 2021 | Self-formulated Tiaowei decoction | Modified/individualized | 黄芪 30 g; 党参 15 g; 莪术 15 g; 白术 12 g; 黄芩 12 g; 炙甘草 10 g; 百合 10 g; 佛手 10 g; 石斛 10 g; 黄连 6 g; 三七粉 8 g | *黄芪: Astragalus mongholicus Bunge; 党参: Codonopsis pilosula (Franch.) Nannf.; 莪术: Curcuma spp. (species not specified); 白术: Atractylodes macrocephala Koidz.; 黄芩: Scutellaria baicalensis Georgi; 甘草: Glycyrrhiza uralensis Fisch. ex DC.; 百合: Lilium brownii var. viridulum Baker; 佛手: Citrus medica var. sarcodactylis (Noot.) Swingle; 石斛: Dendrobium spp. (species not specified); 黄连: Coptis chinensis Franch.; 三七: Panax notoginseng (Burkill) F.H.Chen* | Acid regurgitation: 海螵蛸 10 g; severe pain: 延胡索 10 g and 川楝子 10 g; hypochondrial fullness/belching: 郁金 12 g; phlegm vomiting: 法半夏 12 g and reduce 石斛 and 百合 by 6 g each | 1 decoction/day; 400 mL | Oral decoction 30 min after meals | Morning and evening | 4 weeks |
| S52 | Zhang | 2023 | Weiwei granules | Fixed formula | 党参 15 g; 黄芪 15 g; 白术 7.5 g; 白花蛇舌草 7.5 g; 黄精 7.5 g; 枸杞子 7.5 g; 半枝莲 7.5 g; 麦冬 7.5 g; 石斛 7.5 g; 三七 5 g; 莪术 5 g; 三棱 5 g | *党参: Codonopsis pilosula (Franch.) Nannf.; 黄芪: Astragalus mongholicus Bunge; 白术: Atractylodes macrocephala Koidz.; 白花蛇舌草: Scleromitrion diffusum (Willd.) R.J.Wang (syn. Hedyotis diffusa Willd.); 黄精: Polygonatum spp. (species not specified); 枸杞子: Lycium barbarum L.; 半枝莲: Scutellaria barbata D.Don; 麦冬: Ophiopogon japonicus (Thunb.) Ker Gawl.; 石斛: Dendrobium spp. (species not specified); 三七: Panax notoginseng (Burkill) F.H.Chen; 莪术: Curcuma spp. (species not specified); 三棱: Sparganium stoloniferum (Buch.-Ham. ex Graebn.) Buch.-Ham. ex Juz.* | None reported | 20 g per dose | Oral granules in boiled water, 30 min before meals | Twice daily | 6 months |
| H01 | Cai | 2016 | Shugan Hewei formula | Modified/individualized | 枳壳 10 g; 川芎 10 g; 柴胡 12 g; 白芍 12 g; 香附 12 g; 延胡索 12 g; 佛手 12 g | *枳壳: Citrus aurantium L.; 川芎: Conioselinum anthriscoides 'Chuanxiong' (syn. Ligusticum chuanxiong); 柴胡: Bupleurum chinense DC.; 白芍: Paeonia lactiflora Pall.; 香附: Cyperus rotundus L.; 延胡索: Corydalis yanhusuo W.T.Wang; 佛手: Citrus medica var. sarcodactylis (Noot.) Swingle* | Acid regurgitation: 瓦楞子 30 g and 白及 10 g; qi deficiency: 人参 10 g and 白术 10 g; loose stool: 薏苡仁 30 g; upper abdominal pain: 三七粉 3 g | 1 decoction/day | Oral decoction | Morning and evening | 3 months |
| H02 | Shen | 2007 | Danshen Yin plus modified Shixiao San | Modified/individualized | 砂仁 10 g; 檀香 10 g; 苏梗 5 g; 丹参 30 g; 橘络 10 g; 炒延胡索 10 g; 大黄 10 g; 五灵脂 5 g; 蒲黄 5 g; 甘草 3 g | *砂仁: Wurfbainia villosa (Lour.) Skornick. & A.D.Poulsen (syn. Amomum villosum Lour.); 檀香: Santalum album L.; 苏梗: Perilla frutescens (L.) Britton; 丹参: Salvia miltiorrhiza Bunge; 橘络: Citrus reticulata Blanco; 延胡索: Corydalis yanhusuo W.T.Wang; 大黄: Rheum palmatum L.; 五灵脂: Trogopterus xanthipes Milne-Edwards (animal-derived); 蒲黄: Typha spp. (pollen); 甘草: Glycyrrhiza uralensis Fisch. ex DC.* | None reported | 1 decoction/day | Oral decoction | NR | 3 months |
| H03 | Ding | 2019 | Nuangan decoction | Fixed formula | 当归 15 g; 枸杞子 15 g; 茯苓 15 g; 乌药 2 g; 小茴香 20 g; 肉桂 10 g; 木香 5 g; 生姜 3 slices | *当归: Angelica sinensis (Oliv.) Diels; 枸杞子: Lycium barbarum L.; 茯苓: Wolfiporia cocos (F.A.Wolf) Ryvarden & Gilb. (syn. Poria cocos); 乌药: Lindera aggregata (Sims) Kosterm.; 小茴香: Foeniculum vulgare Mill.; 肉桂: Cinnamomum cassia (L.) J.Presl; 木香: Aucklandia costus Falc.; 生姜: Zingiber officinale Roscoe* | None reported | 1 decoction/day | Oral decoction | Morning and evening | 30 days |
| H04 | Du | 2015 | Yangyin Huowei mixture | Fixed formula; source did not report individual doses | 芦根; 鸡内金; 莪术; 海螵蛸; 旋覆花; 丹皮; 白术; 茯苓; 甘草; 砂仁 | *芦根: Phragmites australis (Cav.) Trin. ex Steud.; 鸡内金: Galli Gigerii Endothelium Corneum (animal-derived); 莪术: Curcuma spp. (species not specified); 海螵蛸: Sepiae Endoconcha (Sepia spp.; animal-derived); 旋覆花: Inula japonica Thunb.; 丹皮: Paeonia suffruticosa Andrews; 白术: Atractylodes macrocephala Koidz.; 茯苓: Wolfiporia cocos (F.A.Wolf) Ryvarden & Gilb. (syn. Poria cocos); 甘草: Glycyrrhiza uralensis Fisch. ex DC.; 砂仁: Wurfbainia villosa (Lour.) Skornick. & A.D.Poulsen (syn. Amomum villosum Lour.)* | None reported | 1 packet dispersed in 400 mL boiling water | Oral instant-granule mixture 30 min after meals | Morning and evening | 8 weeks |
| H05 | Feng | 2011 | Moluodan | Proprietary product; partial source-reported ingredients | 百合; 麦冬; 石斛; 茯苓; 白术; 三七; 延胡索; 乌药; 鸡内金; 玄参; 当归; 川芎; 茵陈; 蒲黄; 白芍 | *百合: Lilium brownii var. viridulum Baker; 麦冬: Ophiopogon japonicus (Thunb.) Ker Gawl.; 石斛: Dendrobium spp. (species not specified); 茯苓: Wolfiporia cocos (F.A.Wolf) Ryvarden & Gilb. (syn. Poria cocos); 白术: Atractylodes macrocephala Koidz.; 三七: Panax notoginseng (Burkill) F.H.Chen; 延胡索: Corydalis yanhusuo W.T.Wang; 乌药: Lindera aggregata (Sims) Kosterm.; 鸡内金: Galli Gigerii Endothelium Corneum (animal-derived); 玄参: Scrophularia ningpoensis Hemsl.; 当归: Angelica sinensis (Oliv.) Diels; 川芎: Conioselinum anthriscoides 'Chuanxiong' (syn. Ligusticum chuanxiong); 茵陈: Artemisia capillaris Thunb.; 蒲黄: Typha spp. (pollen); 白芍: Paeonia lactiflora Pall.* | The article states 18 ingredients but names 15 in the text; no individual doses reported | 1 pill per dose | Oral pill | Three times daily | 3 months |
| H06 | Guo | 2023 | Modified Chaihu Shugan San plus Qingzhong Tang | Modified/individualized | 柴胡 10 g; 枳壳 10 g; 川芎 10 g; 甘草 10 g; 白芍 30 g; 香附 12 g | *柴胡: Bupleurum chinense DC.; 枳壳: Citrus aurantium L.; 川芎: Conioselinum anthriscoides 'Chuanxiong' (syn. Ligusticum chuanxiong); 甘草: Glycyrrhiza uralensis Fisch. ex DC.; 白芍: Paeonia lactiflora Pall.; 香附: Cyperus rotundus L.* | Severe qi stagnation: 青皮 15 g and 郁金 15 g; hypochondrial pain: 川楝子 12 g and 延胡索 15 g; frequent belching: 沉香 6 g and 旋覆花 15 g; acid regurgitation: 乌贼骨 15 g and 煅瓦楞子 20 g | 1 decoction/day; 220 mL reduced to 180 mL | Oral decoction on an empty stomach | Morning and evening | NR |
| H07 | Hu | 2016 | Chaihu Shugan pill | Proprietary product; source-reported ingredients | 白芍; 柴胡; 党参; 半夏; 枳壳; 赤芍; 白术; 黄连; 川楝子; 川芎; 炙甘草 | *白芍: Paeonia lactiflora Pall.; 柴胡: Bupleurum chinense DC.; 党参: Codonopsis pilosula (Franch.) Nannf.; 半夏: Pinellia ternata (Thunb.) Makino; 枳壳: Citrus aurantium L.; 赤芍: Paeonia lactiflora Pall. / Paeonia veitchii Lynch; 白术: Atractylodes macrocephala Koidz.; 黄连: Coptis chinensis Franch.; 川楝子: Melia toosendan Siebold & Zucc.; 川芎: Conioselinum anthriscoides 'Chuanxiong' (syn. Ligusticum chuanxiong); 甘草: Glycyrrhiza uralensis Fisch. ex DC.* | None reported | 1 pill per dose | Oral pill | Twice daily | 4 months |
| H08 | Hu | 2019 | Huatan Xiaoyu formula | Fixed formula | 陈皮 12 g; 法半夏 10 g; 鸡内金 10 g; 紫丹参 15 g; 薏苡仁 30 g; 蒲黄粉 10 g; 半枝莲 15 g; 仙鹤草 15 g; 猫爪草 15 g | *陈皮: Citrus reticulata Blanco; 半夏: Pinellia ternata (Thunb.) Makino; 鸡内金: Galli Gigerii Endothelium Corneum (animal-derived); 丹参: Salvia miltiorrhiza Bunge; 薏苡仁: Coix lacryma-jobi var. ma-yuen (Rom.Caill.) Stapf; 蒲黄: Typha spp. (pollen); 半枝莲: Scutellaria barbata D.Don; 仙鹤草: Agrimonia pilosa Ledeb.; 猫爪草: Ranunculus ternatus Thunb.* | None reported | 1 decoction/day; 200 mL | Oral decoction | Morning and evening | 3 months |
| H09 | Jin | 2012 | Shugan Heluo granules | Fixed formula | 橘核 10 g; 赤芍 10 g; 煅牡蛎 20 g; 泽兰 12 g; 柴胡 12 g; 青皮 10 g; 萆薢 10 g; 陈皮 10 g; 续断 10 g; 泽泻 10 g; 大枣 30 g; 白芍 10 g | *橘核: Citrus reticulata Blanco; 赤芍: Paeonia lactiflora Pall. / Paeonia veitchii Lynch; 牡蛎: Ostreae Concha (Ostrea spp.; animal-derived); 泽兰: Lycopus lucidus Turcz. ex Benth.; 柴胡: Bupleurum chinense DC.; 青皮: Citrus reticulata Blanco; 萆薢: Dioscorea spp. (species not specified); 陈皮: Citrus reticulata Blanco; 续断: Dipsacus asper Wall. ex C.B.Clarke; 泽泻: Alisma plantago-aquatica subsp. orientale (Sam.) Sam.; 大枣: Ziziphus jujuba Mill.; 白芍: Paeonia lactiflora Pall.* | None reported | 1 prescription/day | Oral granules in warm water, 2 h after meals | Twice daily | 12 weeks |
| H10 | Ke | 2025 | Weixu Yuzu prescription | Fixed formula | 黄芪 30 g; 党参 10 g; 莪术 10 g; 木香 10 g; 郁金 10 g; 麦冬 15 g; 枳壳 10 g; 当归 15 g; 麸炒白术 15 g; 熟地黄 15 g; 石斛 20 g; 丹参 20 g; 三七粉 3 g | *黄芪: Astragalus mongholicus Bunge; 党参: Codonopsis pilosula (Franch.) Nannf.; 莪术: Curcuma spp. (species not specified); 木香: Aucklandia costus Falc.; 郁金: Curcuma spp. (species not specified); 麦冬: Ophiopogon japonicus (Thunb.) Ker Gawl.; 枳壳: Citrus aurantium L.; 当归: Angelica sinensis (Oliv.) Diels; 白术: Atractylodes macrocephala Koidz.; 地黄: Rehmannia glutinosa (Gaertn.) DC.; 石斛: Dendrobium spp. (species not specified); 丹参: Salvia miltiorrhiza Bunge; 三七: Panax notoginseng (Burkill) F.H.Chen* | None reported | 1 decoction/day; 150 mL | Oral decoction | Morning and evening | 3 months |
| H11 | Peng | 2022 | Shengyang Yiwei decoction | Modified/individualized | 黄芪 20 g; 半夏 15 g; 人参 15 g; 炙甘草 9 g; 白芍 15 g; 独活 9 g; 防风 9 g; 羌活 9 g; 橘皮 6 g; 茯苓 5 g; 柴胡 5 g; 泽泻 5 g; 白术 5 g; 黄连 1.5 g | *黄芪: Astragalus mongholicus Bunge; 半夏: Pinellia ternata (Thunb.) Makino; 人参: Panax ginseng C.A.Mey.; 甘草: Glycyrrhiza uralensis Fisch. ex DC.; 白芍: Paeonia lactiflora Pall.; 独活: Angelica biserrata (R.H.Shan & C.Q.Yuan) C.Q.Yuan & R.H.Shan; 防风: Saposhnikovia divaricata (Turcz.) Schischk.; 羌活: Notopterygium incisum K.C.Ting ex H.T.Chang; 橘皮: Citrus reticulata Blanco; 茯苓: Wolfiporia cocos (F.A.Wolf) Ryvarden & Gilb. (syn. Poria cocos); 柴胡: Bupleurum chinense DC.; 泽泻: Alisma plantago-aquatica subsp. orientale (Sam.) Sam.; 白术: Atractylodes macrocephala Koidz.; 黄连: Coptis chinensis Franch.* | Constipation: 淫羊藿 and 熟大黄; severe loose stool: 炒扁豆 and 炒薏苡仁; marked burning/acid: 煅瓦楞子 and 海螵蛸; distension: 厚朴 and 枳实 | 1 decoction/day; 300 mL | Oral decoction | Morning and evening | 3 months |
| H12 | Wang | 2012 | Qifang Weitong granules | Fixed formula | 党参 20 g; 白术 10 g; 黄芪 30 g; 茯苓 10 g; 炙甘草 6 g; 白芍 30 g; 木香 6 g; 炒鸡内金 9 g; 枳实 6 g; 丹参 10 g | *党参: Codonopsis pilosula (Franch.) Nannf.; 白术: Atractylodes macrocephala Koidz.; 黄芪: Astragalus mongholicus Bunge; 茯苓: Wolfiporia cocos (F.A.Wolf) Ryvarden & Gilb. (syn. Poria cocos); 甘草: Glycyrrhiza uralensis Fisch. ex DC.; 白芍: Paeonia lactiflora Pall.; 木香: Aucklandia costus Falc.; 鸡内金: Galli Gigerii Endothelium Corneum (animal-derived); 枳实: Citrus aurantium L.; 丹参: Salvia miltiorrhiza Bunge* | None reported | 1 prescription/day | Oral granules | Twice daily | 8 weeks |
| H13 | Xiang | 2007 | Wushen decoction | Fixed formula | 党参 15 g; 沙参 12 g; 玄参 10 g; 丹参 10 g; 苦参 10 g; 麦冬 15 g; 黄精 10 g; 川芎 10 g; 黄连 10 g; 黄芪 12 g; 石斛 15 g; 旋覆花 10 g | *党参: Codonopsis pilosula (Franch.) Nannf.; 沙参: Adenophora spp. (species not specified); 玄参: Scrophularia ningpoensis Hemsl.; 丹参: Salvia miltiorrhiza Bunge; 苦参: Sophora flavescens Aiton; 麦冬: Ophiopogon japonicus (Thunb.) Ker Gawl.; 黄精: Polygonatum spp. (species not specified); 川芎: Conioselinum anthriscoides 'Chuanxiong' (syn. Ligusticum chuanxiong); 黄连: Coptis chinensis Franch.; 黄芪: Astragalus mongholicus Bunge; 石斛: Dendrobium spp. (species not specified); 旋覆花: Inula japonica Thunb.* | None reported | 1 decoction/day | Oral decoction | NR | 3 months |
| H14 | Xu | 2021 | Wanfu Qutong decoction | Modified/individualized | 北沙参 20 g; 桑寄生 20 g; 玉竹 15 g; 白芍 15 g; 山药 10 g; 石斛 15 g; 延胡索 15 g; 香附 10 g; 焦山楂 10 g; 黄芪 15 g; 浙贝母 15 g; 桂枝 10 g; 甘草 6 g | *北沙参: Glehnia littoralis F.Schmidt ex Miq.; 桑寄生: Taxillus chinensis (DC.) Danser; 玉竹: Polygonatum odoratum (Mill.) Druce; 白芍: Paeonia lactiflora Pall.; 山药: Dioscorea polystachya Turcz.; 石斛: Dendrobium spp. (species not specified); 延胡索: Corydalis yanhusuo W.T.Wang; 香附: Cyperus rotundus L.; 山楂: Crataegus pinnatifida Bunge; 黄芪: Astragalus mongholicus Bunge; 浙贝母: Fritillaria thunbergii Miq.; 桂枝: Cinnamomum cassia (L.) J.Presl; 甘草: Glycyrrhiza uralensis Fisch. ex DC.* | Source reported symptom-based additions including 丹参, 枳实, 海螵蛸 and others | 1 decoction/day; about 300 mL | Oral decoction | Twice daily | 3 months |
| H15 | Ye | 2024 | Yiweining capsules | Hospital preparation; source-reported ingredients | 党参; 黄芪; 麸炒白术; 茯苓; 赤芍; 砂仁; 炙甘草; 枳壳; 黄连 | *党参: Codonopsis pilosula (Franch.) Nannf.; 黄芪: Astragalus mongholicus Bunge; 白术: Atractylodes macrocephala Koidz.; 茯苓: Wolfiporia cocos (F.A.Wolf) Ryvarden & Gilb. (syn. Poria cocos); 赤芍: Paeonia lactiflora Pall. / Paeonia veitchii Lynch; 砂仁: Wurfbainia villosa (Lour.) Skornick. & A.D.Poulsen (syn. Amomum villosum Lour.); 甘草: Glycyrrhiza uralensis Fisch. ex DC.; 枳壳: Citrus aurantium L.; 黄连: Coptis chinensis Franch.* | None reported | 4 capsules per dose (0.5 g/capsule; 2 g per dose) | Oral capsules before meals | Three times daily | 12 weeks |
| H16 | Ye | 2020 | Erchen Pingwei decoction | Fixed formula | 苍术 10 g; 厚朴 12 g; 陈皮 10 g; 制半夏 10 g; 茯苓 15 g; 炙甘草 6 g | *苍术: Atractylodes lancea (Thunb.) DC.; 厚朴: Magnolia officinalis Rehder & E.H.Wilson; 陈皮: Citrus reticulata Blanco; 半夏: Pinellia ternata (Thunb.) Makino; 茯苓: Wolfiporia cocos (F.A.Wolf) Ryvarden & Gilb. (syn. Poria cocos); 甘草: Glycyrrhiza uralensis Fisch. ex DC.* | None reported | 1 decoction/day; 400 mL | Oral decoction | Morning and evening | 24 weeks |
| H17 | Zhu | 2009 | Yangwei granules | Proprietary product; source-reported main ingredients | 黄芪; 白芍; 延胡索; 甘草 | *黄芪: Astragalus mongholicus Bunge; 白芍: Paeonia lactiflora Pall.; 延胡索: Corydalis yanhusuo W.T.Wang; 甘草: Glycyrrhiza uralensis Fisch. ex DC.* | None reported | 5 g per dose | Oral granules in warm water | Three times daily | 6 months |
| H18 | Zhu | 2010 | Jianwei Xiaoweiling decoction | Modified/individualized | 白芍 15 g; 柴胡 10 g; 党参 15 g; 枳壳 10 g; 白术 10 g; 茯苓 10 g; 莪术 10 g; 半夏 10 g | *白芍: Paeonia lactiflora Pall.; 柴胡: Bupleurum chinense DC.; 党参: Codonopsis pilosula (Franch.) Nannf.; 枳壳: Citrus aurantium L.; 白术: Atractylodes macrocephala Koidz.; 茯苓: Wolfiporia cocos (F.A.Wolf) Ryvarden & Gilb. (syn. Poria cocos); 莪术: Curcuma spp. (species not specified); 半夏: Pinellia ternata (Thunb.) Makino* | Liver–stomach disharmony: 香附 10 g and 佛手 10 g; qi deficiency/stasis: 丹参 15 g, 五灵脂 10 g and 蒲黄 10 g; yin deficiency: replace 党参 with 北沙参 and add 石斛 15 g; spleen deficiency: 黄芪 20 g and 山药 20 g; damp heat: 藿香 10 g and 蒲公英 20 g | 1 decoction/day; 400 mL split into two 200 mL packs | Oral decoction | Morning and evening | 6 courses (14 days/course) |
| H19 | Zhu | 2021 | Modified Huatan Xiaoyu formula | Modified/individualized | 陈皮 10 g; 法半夏 10 g; 茯苓 15 g; 丹参 15 g; 薏苡仁 30 g; 莪术 10 g; 鸡内金 10 g; 白花蛇舌草 15 g; 仙鹤草 15 g; 半枝莲 15 g; 藤梨根 20 g; 吴茱萸 3 g; 黄连 3 g; 乌贼骨 20 g; 瓦楞子 30 g | *陈皮: Citrus reticulata Blanco; 半夏: Pinellia ternata (Thunb.) Makino; 茯苓: Wolfiporia cocos (F.A.Wolf) Ryvarden & Gilb. (syn. Poria cocos); 丹参: Salvia miltiorrhiza Bunge; 薏苡仁: Coix lacryma-jobi var. ma-yuen (Rom.Caill.) Stapf; 莪术: Curcuma spp. (species not specified); 鸡内金: Galli Gigerii Endothelium Corneum (animal-derived); 白花蛇舌草: Scleromitrion diffusum (Willd.) R.J.Wang (syn. Hedyotis diffusa Willd.); 仙鹤草: Agrimonia pilosa Ledeb.; 半枝莲: Scutellaria barbata D.Don; 藤梨根: Actinidia chinensis Planch.; 吴茱萸: Tetradium ruticarpum (A.Juss.) T.G.Hartley; 黄连: Coptis chinensis Franch.; 乌贼骨: Sepiae Endoconcha (Sepia spp.; animal-derived); 瓦楞子: Arcae Concha (Arca spp.; animal-derived)* | For CAG with intestinal metaplasia/dysplasia, 茯苓 was replaced by 猪苓 10 g. Additional symptom-based modifications were reported for pain, fullness, belching, early satiety, throat discomfort, insomnia, anxiety and yin deficiency | 1 decoction/day; 200 mL | Oral decoction | Morning and evening | 12 weeks |
| H20 | Zou | 2024 | Moluodan (eligible add-on arm: Moluodan + folic acid) | Proprietary product; composition not reported | NR | *NR* | None reported; folic acid was the shared conventional background | Moluodan 1 packet per dose; folic acid 10 mg/week in the shared background | Oral proprietary product | Moluodan three times daily; folic acid one tablet twice weekly | 12 months |
| U02 | Xu | 2026 | Chaigui decoction | Fixed formula | 北柴胡 15 g; 党参 10 g; 炒白术 15 g; 茯苓 15 g; 大枣 20 g; 炙甘草 10 g; 当归 10 g; 白芍 15 g; 川芎 15 g; 炒黄芩 10 g; 泽泻 15 g; 姜半夏 15 g; 生姜 10 g | *柴胡: Bupleurum chinense DC.; 党参: Codonopsis pilosula (Franch.) Nannf.; 白术: Atractylodes macrocephala Koidz.; 茯苓: Wolfiporia cocos (F.A.Wolf) Ryvarden & Gilb. (syn. Poria cocos); 大枣: Ziziphus jujuba Mill.; 甘草: Glycyrrhiza uralensis Fisch. ex DC.; 当归: Angelica sinensis (Oliv.) Diels; 白芍: Paeonia lactiflora Pall.; 川芎: Conioselinum anthriscoides 'Chuanxiong' (syn. Ligusticum chuanxiong); 黄芩: Scutellaria baicalensis Georgi; 泽泻: Alisma plantago-aquatica subsp. orientale (Sam.) Sam.; 半夏: Pinellia ternata (Thunb.) Makino; 生姜: Zingiber officinale Roscoe* | None reported | 1 decoction/day; 600 mL | Oral decoction 1 h after meals | Morning and evening | 12 weeks |
| U04 | Wang | 2026 | Shuwei formula | Modified/individualized | 柴胡 12 g; 白芍 15 g; 枳壳 10 g; 党参 15 g; 白术 15 g; 丹参 15 g; 莪术 9 g; 麦冬 12 g; 法半夏 9 g; 合欢皮 15 g; 炙甘草 6 g | *柴胡: Bupleurum chinense DC.; 白芍: Paeonia lactiflora Pall.; 枳壳: Citrus aurantium L.; 党参: Codonopsis pilosula (Franch.) Nannf.; 白术: Atractylodes macrocephala Koidz.; 丹参: Salvia miltiorrhiza Bunge; 莪术: Curcuma spp. (species not specified); 麦冬: Ophiopogon japonicus (Thunb.) Ker Gawl.; 半夏: Pinellia ternata (Thunb.) Makino; 合欢皮: Albizia julibrissin Durazz.; 甘草: Glycyrrhiza uralensis Fisch. ex DC.* | Severe pain: 延胡索 12 g and 川楝子 9 g; frequent belching: 旋覆花 9 g and 代赭石 15 g; dry mouth/constipation: 北沙参 12 g and 火麻仁 15 g; insomnia: 酸枣仁 20 g and 远志 9 g | 1 decoction/day; 400 mL; 200 mL per dose | Oral decoction | Morning and evening | 12 weeks |

**Panel D. Control and conventional-therapy regimen**

| **Study ID** | **First author** | **Year** | **Control type** | **Conventional drug or regimen** | **Dose** | **Frequency** | **Administration** | **Treatment duration** | **Background therapy** |
| --- | --- | --- | --- | --- | --- | --- | --- | --- | --- |
| S1 | Wei | 2025 | Conventional therapy | Hp-positive patients: omeprazole 20 mg bid or lansoprazole 30 mg bid or esomeprazole 20 mg bid; clarithromycin 0.5 g bid; amoxicillin 1 g bid; hydrotalcite 0.5 g tid | Specified in regimen | Specified in regimen | Oral | 4 weeks | None reported |
| S2 | Xi | 2025 | Conventional therapy | Hp-positive patients: omeprazole 20 mg bid, bismuth potassium citrate 0.6 g bid, clarithromycin 0.5 g bid and amoxicillin 1 g bid for 14 days; thereafter rebamipide 100 mg tid; mosapride 5 mg tid as needed | Specified in regimen | Specified in regimen | Oral | 3 months | None reported |
| S3 | Zhang | 2022 | Conventional therapy | Pepsin mixture 10 mL tid before meals; furazolidone 120 mg qid; cholestyramine 4 g tid; additional source-reported conventional therapy | Specified in regimen | Specified in regimen | Oral | 3 months | None reported |
| S4 | Shen | 2017 | Active conventional-therapy comparator | Teprenone 50 mg tid | Specified in regimen | Specified in regimen | Oral | 4 months | None reported |
| S5 | Yuan | 2026 | Active conventional-therapy comparator | Omeprazole 20 mg bid plus colloidal bismuth pectin 3 capsules qid before meals/at bedtime | Specified in regimen | Specified in regimen | Oral | 8 weeks | None reported |
| S6 | You | 2021 | Conventional therapy | conventional therapy | See source regimen | See source regimen | Oral unless otherwise stated | 6 months | Same control/background therapy plus CHM in experimental arm |
| S7 | Gao | 2022 | Active conventional-therapy comparator | Vitacoenzyme capsules, 2 capsules (0.2 g/capsule) tid | Specified in regimen | Specified in regimen | Oral | 3 months | None reported |
| S8 | Yi | 2022 | Conventional therapy | Omeprazole 20 mg/day; teprenone 150 mg/day in 3 doses; mosapride 15 mg/day in 3 doses | Specified in regimen | Specified in regimen | Oral | 8 weeks | None reported |
| S9 | Zhang | 2016 | Active conventional-therapy comparator | Ursodeoxycholic acid 250 mg qd on the source-reported intermittent schedule; hydrotalcite 0.5 g tid then bid | Specified in regimen | Specified in regimen | Oral | 24 weeks | None reported |
| S10 | Zhang | 2020 | Conventional therapy | Lansoprazole 30 mg qd plus teprenone 50 mg tid | Specified in regimen | Specified in regimen | Oral | 6 months | None reported |
| S11 | Huang | 2020 | Conventional therapy | Amoxicillin 0.25 g bid, omeprazole 20 mg bid and clarithromycin 0.5 g bid for 2 weeks; folic acid 10 mg tid for 4 weeks | Specified in regimen | Specified in regimen | Oral | 4 weeks | None reported |
| S13 | Liu | 2005 | Active conventional-therapy comparator | Vitacoenzyme 3 tablets tid plus sucralfate 2 tablets tid | Specified in regimen | Specified in regimen | Oral | 20 days | None reported |
| S14 | Liu | 2017 | Active conventional-therapy comparator | Teprenone 50 mg tid | Specified in regimen | Specified in regimen | Oral | 4 months | None reported |
| S15 | Wang | 2025 | Active conventional-therapy comparator | Amoxicillin 1 g bid, clarithromycin 0.5 g bid and omeprazole 20 mg bid | Specified in regimen | Specified in regimen | Oral | 10 days | None reported |
| S16 | Zhuang | 2011 | Conventional therapy | Pectin bismuth 150 mg tid, domperidone 10 mg tid and amoxicillin 50 mg tid; omeprazole 20 mg qd if reflux | Specified in regimen | Specified in regimen | Oral | 3 months | None reported |
| S17 | Wang | 2021 | Conventional therapy | Pectin bismuth 100 mg tid, domperidone 10 mg tid and amoxicillin 50 mg tid; omeprazole 20 mg qd if reflux | Specified in regimen | Specified in regimen | Oral | 3 months | None reported |
| S18 | Zhang | 2013 | Conventional therapy | Folic acid 5 mg tid; hydrotalcite 2 tablets tid if severe pain; mosapride 1 tablet tid if distension | Specified in regimen | Specified in regimen | Oral | 8 weeks | None reported |
| S19 | Shen | 2021 | Conventional therapy | Pantoprazole 20 mg bid, amoxicillin 1 g bid and teprenone 50 mg tid | Specified in regimen | Specified in regimen | Oral | 8 weeks | None reported |
| S20 | Wang | 2023 | Conventional therapy | Lansoprazole 30 mg qd plus teprenone 50 mg tid | Specified in regimen | Specified in regimen | Oral | 12 weeks | None reported |
| S21 | Zhang | 2025 | Active conventional-therapy comparator | Bismuth potassium citrate 0.3 g qid, omeprazole 20 mg bid, amoxicillin 0.5 g every 8 h and extended-release clarithromycin 0.5 g qd | Specified in regimen | Specified in regimen | Oral | 2 weeks | None reported |
| S22 | Jia | 2021 | Active conventional-therapy comparator | Omeprazole 20 mg bid, bismuth potassium citrate 0.3 g tid, furazolidone 0.1 g tid and amoxicillin 0.5 g tid; antibiotics stopped after 2 weeks | Specified in regimen | Specified in regimen | Oral | 12 weeks | None reported |
| S24 | Yang | 2024 | Active conventional-therapy comparator | Clarithromycin 0.5 g bid, amoxicillin 0.5 g bid, rabeprazole 20 mg bid and colloidal bismuth pectin 0.2 g bid | Specified in regimen | Specified in regimen | Oral | 8 weeks | None reported |
| S26 | Wang | 2018 | Active conventional-therapy comparator | Mosapride 5 mg tid before meals plus hydrotalcite 1 g tid after meals | Specified in regimen | Specified in regimen | Oral | 4 weeks | None reported |
| S27 | Ning | 2016 | Active conventional-therapy comparator | Vitacoenzyme 0.2 g × 4 tablets tid | Specified in regimen | Specified in regimen | Oral | 3 months | None reported |
| S28 | Qi | 2024 | Active conventional-therapy comparator | Quadruple therapy | See source regimen | See source regimen | Oral unless otherwise stated | 3 months | Same control/background therapy plus CHM in experimental arm |
| S29 | Dong | 2024 | Active conventional-therapy comparator | Folic acid 5 mg tid | Specified in regimen | Specified in regimen | Oral | 18 weeks | None reported |
| S30 | Yang | 2017 | Active conventional-therapy comparator | Triple therapy | See source regimen | See source regimen | Oral unless otherwise stated | 2 months | Same control/background therapy plus CHM in experimental arm |
| S31 | Xu | 2018 | Conventional therapy | Hydrotalcite 0.5 g tid, pantoprazole 40 mg bid, clarithromycin 250 mg bid and amoxicillin 1 g bid | Specified in regimen | Specified in regimen | Oral | 2 months | None reported |
| S32 | Liu | 2010 | Active conventional-therapy comparator | H. pylori eradication therapy | See source regimen | See source regimen | Oral unless otherwise stated | 6 months | Same control/background therapy plus CHM in experimental arm |
| S33 | Xing | 2004 | Active conventional-therapy comparator | Triple therapy | See source regimen | See source regimen | Oral unless otherwise stated | 3 months | Same control/background therapy plus CHM in experimental arm |
| S34 | Jiang | 2025 | Conventional therapy | Teprenone 50 mg tid | Specified in regimen | Specified in regimen | Oral | 12 weeks | None reported |
| S35 | Zhang | 2021 | Conventional therapy | Domperidone 10 mg tid, lactasin 0.4 g tid and omeprazole 20 mg tid | Specified in regimen | Specified in regimen | Oral | 2 months | None reported |
| S37 | Bu | 2019 | Active conventional-therapy comparator | Rebamipide 0.1 g tid | Specified in regimen | Specified in regimen | Oral | 3 months | None reported |
| S38 | Yu | 2023 | Conventional therapy | Amoxicillin 0.5 g bid, omeprazole 20 mg bid and clarithromycin 0.5 g qd | Specified in regimen | Specified in regimen | Oral | 1 month | None reported |
| S39 | Xu | 2021 | Active conventional-therapy comparator | Vitacoenzyme 1.2 g tid | Specified in regimen | Specified in regimen | Oral | 3 months | None reported |
| S40 | Li | 2019 | Conventional therapy | Omeprazole, domperidone and colloidal bismuth pectin (source-specific doses) | Specified in regimen | Specified in regimen | Oral | 8 weeks | None reported |
| S42 | Sun | 2017 | Active conventional-therapy comparator | Folic acid 5 mg tid | Specified in regimen | Specified in regimen | Oral | 6 months | None reported |
| S43 | Wang | 2010 | Conventional therapy | Routine conventional therapy individualized for Hp, reflux and dyspepsia | Specified in regimen | Specified in regimen | Oral | 6 months | None reported |
| S44 | Zhang | 2008 | Conventional therapy | conventional therapy | See source regimen | See source regimen | Oral unless otherwise stated | 2–5 courses (30 days/course) | Same control/background therapy plus CHM in experimental arm |
| S45 | Zhang | 2004 | Conventional therapy | Omeprazole 20 mg each morning; amoxicillin 0.5 g bid; metronidazole 0.4 g bid; folic acid 50 mg bid | Specified in regimen | Specified in regimen | Oral | 8 weeks | None reported |
| S46 | Fu | 2020 | Conventional therapy | Domperidone 10 mg tid before meals plus sucralfate 1 g qid | Specified in regimen | Specified in regimen | Oral | 6 months | None reported |
| S47 | Wang | 2003 | Conventional therapy | Colloidal bismuth 100 mg tid, tinidazole 400 mg tid, vitamin C and folic acid | Specified in regimen | Specified in regimen | Oral | 2 weeks | None reported |
| S48 | Sun | 2020 | Conventional therapy | conventional therapy | See source regimen | See source regimen | Oral unless otherwise stated | 3 months | Same control/background therapy plus CHM in experimental arm |
| S49 | Wu | 2017 | Active conventional-therapy comparator | Famotidine 40 mg at bedtime | Specified in regimen | Specified in regimen | Oral | 30 days | None reported |
| S50 | Lin | 2023 | Active conventional-therapy comparator | Omeprazole 20 mg qd | Specified in regimen | Specified in regimen | Oral | 3 months | None reported |
| S51 | Wang | 2021 | Conventional therapy | Vitacoenzyme 1 g tid; rabeprazole 10 mg bid; Hp-positive patients also amoxicillin 1 g bid for 4 weeks | Specified in regimen | Specified in regimen | Oral | 4 weeks | None reported |
| S52 | Zhang | 2023 | Active conventional-therapy comparator | Rabeprazole 10 mg bid before meals, amoxicillin 1 g bid after meals, furazolidone 100 mg bid after meals and bismuth potassium citrate 0.6 g bid | Specified in regimen | Specified in regimen | Oral | 6 months | None reported |
| H01 | Cai | 2016 | Active conventional-therapy comparator | Vitacoenzyme, 5 tablets tid | Specified in regimen | Specified in regimen | Oral | 3 months | None reported |
| H02 | Shen | 2007 | NR | NR | NR | NR | NR | 3 months | NR |
| H03 | Ding | 2019 | Active conventional-therapy comparator | Omeprazole 20 mg qd, amoxicillin 1 g bid and clarithromycin 0.5 g bid | Specified in regimen | Specified in regimen | Oral | 30 days | None reported |
| H04 | Du | 2015 | NR | Amoxicillin 1 g bid and clarithromycin 0.5 g bid for 2 weeks plus esomeprazole 40 mg qd and bismuth potassium citrate 220 mg bid for 8 weeks | Specified in regimen | Specified in regimen | Oral | 8 weeks | None reported |
| H05 | Feng | 2011 | Placebo / conventional background | Folic acid 10 mg tid plus matched placebo | Specified in regimen | Specified in regimen | Oral | 3 months | None reported |
| H06 | Guo | 2023 | NR | Domperidone 10 mg tid before meals | Specified in regimen | Specified in regimen | Oral | NR | None reported |
| H07 | Hu | 2016 | NR | Amoxicillin 0.5 g tid, clarithromycin 0.25 g bid and rabeprazole 10 mg qd | Specified in regimen | Specified in regimen | Oral | 4 months | None reported |
| H08 | Hu | 2019 | NR | NR | NR | NR | NR | 3 months | NR |
| H09 | Jin | 2012 | NR | Esomeprazole 20 mg bid | Specified in regimen | Specified in regimen | Oral | 12 weeks | None reported |
| H10 | Ke | 2025 | Active conventional-therapy comparator | Rabeprazole 20 mg bid, trimebutine 0.1 g tid and compound bismuth aluminate 1.3 g tid; quadruple therapy if Hp-positive | Specified in regimen | Specified in regimen | Oral | 3 months | None reported |
| H11 | Peng | 2022 | Active conventional-therapy comparator | Quadruple therapy | See source regimen | See source regimen | Oral unless otherwise stated | 3 months | Same control/background therapy plus CHM in experimental arm |
| H12 | Wang | 2012 | Active conventional-therapy comparator | Conventional treatment | See source regimen | See source regimen | Oral unless otherwise stated | 8 weeks | Same control/background therapy plus CHM in experimental arm |
| H13 | Xiang | 2007 | Active conventional-therapy comparator | Sucralfate 1 g tid, mosapride 5 mg tid and folic acid 5 mg qd | Specified in regimen | Specified in regimen | Oral | 3 months | None reported |
| H14 | Xu | 2021 | Active conventional-therapy comparator | Esomeprazole | See source regimen | See source regimen | Oral unless otherwise stated | 3 months | Same |
| H15 | Ye | 2024 | Active conventional-therapy comparator | Rebamipide 0.1 g tid | Specified in regimen | Specified in regimen | Oral | 12 weeks | None reported |
| H16 | Ye | 2020 | Active conventional-therapy comparator | Conventional therapy | See source regimen | See source regimen | Oral unless otherwise stated | 24 weeks | Same control/background therapy plus CHM in experimental arm |
| H17 | Zhu | 2009 | Active conventional-therapy comparator | Multienzyme 2 tablets tid plus vitamin B6 10 mg bid | Specified in regimen | Specified in regimen | Oral | 6 months | None reported |
| H18 | Zhu | 2010 | NR | NR | NR | NR | NR | 6 treatment courses; exact duration unclear | NR |
| H19 | Zhu | 2021 | NR | NR | NR | NR | NR | 12 weeks | NR |
| H20 | Zou | 2024 | Active conventional-therapy comparator | Folic acid alone with Moluodan simulant; eligible comparison is Moluodan + folic acid vs folic acid | Folic acid 10 mg/week (two tablets/week, as reported in the source Discussion) | One folic-acid tablet twice weekly | Oral | 12 months | Folic acid was the shared background therapy; the experimental arm additionally received standard-dose Moluodan |
| U02 | Xu | 2026 | Active conventional-therapy comparator | Teprenone 50 mg tid plus folic acid 5 mg tid | Specified in regimen | Specified in regimen | Oral | 12 weeks | None reported |
| U04 | Wang | 2026 | Active conventional-therapy comparator | Flupentixol/melitracen 2 tablets/day in two doses; folic acid 5 mg tid; hydrotalcite 1 g tid; Hp eradication before enrollment when indicated | Specified in regimen | Specified in regimen | Oral | 12 weeks | None reported |

**Panel E. Reported/extracted outcome domains**

| **Study ID** | **First author** | **Year** | **Pathology-related outcomes** | **Symptom-related outcomes** | **Endoscopic outcomes** | **Serum/gastric-function markers** | **Clinical-response outcomes** | **Inflammatory/immune markers** | **Adverse events** | **Other outcomes** |
| --- | --- | --- | --- | --- | --- | --- | --- | --- | --- | --- |
| S1 | Wei | 2025 | atrophy; dysplasia/IEN; inflammation/activity; intestinal metaplasia | TCM symptom/syndrome scores | Endoscopic/mucosal findings or scores | G-17/gastrin | Clinical total/effective response | IL-1β/IL-6 | NR | NR |
| S2 | Xi | 2025 | atrophy; inflammation/activity; intestinal metaplasia | NR | Endoscopic/mucosal findings or scores | PGI/PGII/PGR; G-17/gastrin | Clinical total/effective response | IL-1β/IL-6; CRP | Reported adverse events | quality of life |
| S3 | Zhang | 2022 | atrophy; inflammation/activity; intestinal metaplasia | TCM symptom/syndrome scores | Endoscopic/mucosal findings or scores | PGI/PGII/PGR; G-17/gastrin; MTL/motilin | Clinical total/effective response | NR | Reported adverse events | NR |
| S4 | Shen | 2017 | atrophy; dysplasia/IEN; inflammation/activity; intestinal metaplasia | NR | NR | PGI/PGII/PGR; G-17/gastrin | Clinical total/effective response | IL-1β/IL-6; TNF-α | Reported adverse events | NR |
| S5 | Yuan | 2026 | atrophy; dysplasia/IEN; inflammation/activity; intestinal metaplasia | TCM symptom/syndrome scores | NR | PGI/PGII/PGR; G-17/gastrin | Clinical total/effective response | IL-1β/IL-6; TNF-α | NR | anxiety/depression scales |
| S6 | You | 2021 | atrophy; dysplasia/IEN; intestinal metaplasia | TCM symptom/syndrome scores | Endoscopic/mucosal findings or scores | PGI/PGII/PGR | Clinical total/effective response | NR | NR | anxiety/depression scales; quality of life |
| S7 | Gao | 2022 | atrophy; dysplasia/IEN; intestinal metaplasia | NR | Endoscopic/mucosal findings or scores | NR | Clinical total/effective response | NR | NR | quality of life |
| S8 | Yi | 2022 | atrophy; dysplasia/IEN; inflammation/activity; intestinal metaplasia | NR | NR | PGI/PGII/PGR; G-17/gastrin | Clinical total/effective response | NR | NR | NR |
| S9 | Zhang | 2016 | atrophy | NR | Endoscopic/mucosal findings or scores | NR | Clinical total/effective response | NR | Reported adverse events | NR |
| S10 | Zhang | 2020 | atrophy | TCM symptom/syndrome scores | Endoscopic/mucosal findings or scores | NR | Clinical total/effective response | NR | Reported adverse events | NR |
| S11 | Huang | 2020 | atrophy; dysplasia/IEN; inflammation/activity; intestinal metaplasia | NR | NR | PGI/PGII/PGR | Clinical total/effective response | NR | NR | quality of life |
| S13 | Liu | 2005 | atrophy | NR | Endoscopic/mucosal findings or scores | NR | Clinical total/effective response | NR | NR | NR |
| S14 | Liu | 2017 | intestinal metaplasia | NR | NR | NR | NR | NR | NR | NR |
| S15 | Wang | 2025 | atrophy; dysplasia/IEN; inflammation/activity; intestinal metaplasia | TCM symptom/syndrome scores | NR | PGI/PGII/PGR | Clinical total/effective response | VEGF; CD markers | Reported adverse events | H. pylori clearance/negative conversion |
| S16 | Zhuang | 2011 | atrophy | NR | Endoscopic/mucosal findings or scores | NR | Clinical total/effective response | NR | Reported adverse events | NR |
| S17 | Wang | 2021 | atrophy | TCM symptom/syndrome scores | NR | PGI/PGII/PGR; G-17/gastrin | Clinical total/effective response | IL-1β/IL-6; CRP; Ki-67 / molecular markers | NR | NR |
| S18 | Zhang | 2013 | atrophy; dysplasia/IEN; intestinal metaplasia | TCM symptom/syndrome scores | NR | NR | Clinical total/effective response | NR | NR | NR |
| S19 | Shen | 2021 | atrophy; dysplasia/IEN; inflammation/activity; intestinal metaplasia | NR | NR | G-17/gastrin | Clinical total/effective response | TNF-α | NR | quality of life |
| S20 | Wang | 2023 | atrophy; inflammation/activity; intestinal metaplasia | TCM symptom/syndrome scores | Endoscopic/mucosal findings or scores | PGI/PGII/PGR | Clinical total/effective response | IL-1β/IL-6; VEGF | NR | NR |
| S21 | Zhang | 2025 | atrophy; dysplasia/IEN; inflammation/activity; intestinal metaplasia | TCM symptom/syndrome scores | NR | PGI/PGII/PGR; SS/somatostatin | Clinical total/effective response | CXCL5/Runx3 | NR | quality of life; H. pylori clearance/negative conversion |
| S22 | Jia | 2021 | atrophy; dysplasia/IEN; intestinal metaplasia | TCM symptom/syndrome scores | Endoscopic/mucosal findings or scores | PGI/PGII/PGR | Clinical total/effective response | IL-1β/IL-6 | NR | anxiety/depression scales |
| S24 | Yang | 2024 | atrophy; dysplasia/IEN; intestinal metaplasia | TCM symptom/syndrome scores | Endoscopic/mucosal findings or scores | PGI/PGII/PGR; G-17/gastrin | Clinical total/effective response | IL-1β/IL-6; TNF-α; CRP; CD markers; Ki-67 / molecular markers | Reported adverse events | quality of life |
| S26 | Wang | 2018 | atrophy; inflammation/activity; intestinal metaplasia | NR | Endoscopic/mucosal findings or scores | SS/somatostatin | NR | NR | NR | anxiety/depression scales |
| S27 | Ning | 2016 | atrophy; inflammation/activity; intestinal metaplasia | TCM symptom/syndrome scores | NR | PGI/PGII/PGR; G-17/gastrin | Clinical total/effective response | NR | Reported in source text | NR |
| S28 | Qi | 2024 | atrophy; dysplasia/IEN; intestinal metaplasia | NR | Endoscopic/mucosal findings or scores | NR | Clinical total/effective response | NR | NR | anxiety/depression scales; H. pylori clearance/negative conversion |
| S29 | Dong | 2024 | atrophy; inflammation/activity; intestinal metaplasia | TCM symptom/syndrome scores | Endoscopic/mucosal findings or scores | NR | Clinical total/effective response | NR | Reported adverse events | quality of life |
| S30 | Yang | 2017 | atrophy; intestinal metaplasia | NR | Endoscopic/mucosal findings or scores | NR | Clinical total/effective response | NR | Reported in source text | NR |
| S31 | Xu | 2018 | atrophy; inflammation/activity; intestinal metaplasia | NR | Endoscopic/mucosal findings or scores | G-17/gastrin | Clinical total/effective response | NR | NR | H. pylori clearance/negative conversion |
| S32 | Liu | 2010 | intestinal metaplasia | NR | Endoscopic/mucosal findings or scores | NR | Clinical total/effective response | NR | NR | H. pylori clearance/negative conversion |
| S33 | Xing | 2004 | intestinal metaplasia | NR | Endoscopic/mucosal findings or scores | NR | Clinical total/effective response | NR | NR | H. pylori clearance/negative conversion |
| S34 | Jiang | 2025 | atrophy; dysplasia/IEN; intestinal metaplasia | NR | Endoscopic/mucosal findings or scores | PGI/PGII/PGR; G-17/gastrin; MTL/motilin | Clinical total/effective response | CXCL5/Runx3 | NR | NR |
| S35 | Zhang | 2021 | atrophy; dysplasia/IEN; inflammation/activity | NR | NR | G-17/gastrin | Clinical total/effective response | NR | NR | quality of life |
| S37 | Bu | 2019 | atrophy; dysplasia/IEN; inflammation/activity; intestinal metaplasia | TCM symptom/syndrome scores | Endoscopic/mucosal findings or scores | PGI/PGII/PGR | Clinical total/effective response | IL-1β/IL-6 | Reported in source text | anxiety/depression scales; H. pylori clearance/negative conversion |
| S38 | Yu | 2023 | atrophy; dysplasia/IEN; intestinal metaplasia | TCM symptom/syndrome scores | Endoscopic/mucosal findings or scores | NR | Clinical total/effective response | IL-1β/IL-6; TNF-α; VEGF; Ki-67 / molecular markers | Reported adverse events | NR |
| S39 | Xu | 2021 | atrophy; dysplasia/IEN; inflammation/activity; intestinal metaplasia | NR | NR | PGI/PGII/PGR | Clinical total/effective response | VEGF; Ki-67 / molecular markers | NR | quality of life |
| S40 | Li | 2019 | atrophy | TCM symptom/syndrome scores | Endoscopic/mucosal findings or scores | NR | Clinical total/effective response | NR | NR | quality of life |
| S42 | Sun | 2017 | atrophy; dysplasia/IEN; inflammation/activity; intestinal metaplasia | NR | Endoscopic/mucosal findings or scores | NR | Clinical total/effective response | NR | NR | H. pylori clearance/negative conversion |
| S43 | Wang | 2010 | atrophy; dysplasia/IEN; intestinal metaplasia | NR | Endoscopic/mucosal findings or scores | NR | Clinical total/effective response | NR | NR | NR |
| S44 | Zhang | 2008 | atrophy; dysplasia/IEN; intestinal metaplasia | NR | Endoscopic/mucosal findings or scores | G-17/gastrin | Clinical total/effective response | NR | NR | NR |
| S45 | Zhang | 2004 | atrophy; dysplasia/IEN; intestinal metaplasia | NR | NR | NR | Clinical total/effective response | NR | NR | anxiety/depression scales; H. pylori clearance/negative conversion |
| S46 | Fu | 2020 | atrophy; dysplasia/IEN; intestinal metaplasia | NR | NR | NR | Clinical total/effective response | NR | NR | quality of life |
| S47 | Wang | 2003 | intestinal metaplasia | NR | NR | NR | NR | NR | NR | NR |
| S48 | Sun | 2020 | atrophy; inflammation/activity | NR | NR | PGI/PGII/PGR | Clinical total/effective response | NR | Reported adverse events | NR |
| S49 | Wu | 2017 | atrophy; intestinal metaplasia | TCM symptom/syndrome scores | NR | PGI/PGII/PGR; G-17/gastrin; SS/somatostatin | Clinical total/effective response | NR | Reported adverse events | H. pylori clearance/negative conversion |
| S50 | Lin | 2023 | atrophy; dysplasia/IEN; inflammation/activity; intestinal metaplasia | TCM symptom/syndrome scores | NR | PGI/PGII/PGR; G-17/gastrin | Clinical total/effective response | NR | NR | H. pylori clearance/negative conversion |
| S51 | Wang | 2021 | atrophy; dysplasia/IEN; intestinal metaplasia | TCM symptom/syndrome scores | Endoscopic/mucosal findings or scores | G-17/gastrin; MTL/motilin; SS/somatostatin | Clinical total/effective response | IL-1β/IL-6; TNF-α; CD markers | Reported adverse events | NR |
| S52 | Zhang | 2023 | atrophy; dysplasia/IEN; inflammation/activity; intestinal metaplasia | TCM symptom/syndrome scores | NR | G-17/gastrin; MTL/motilin; SS/somatostatin | NR | IL-1β/IL-6; TNF-α; VEGF; Ki-67 / molecular markers | Reported adverse events | H. pylori clearance/negative conversion |
| H01 | Cai | 2016 | NR | NR | Endoscopic/mucosal findings or scores | NR | Clinical total/effective response | NR | NR | anxiety/depression scales |
| H02 | Shen | 2007 | NR | NR | Endoscopic/mucosal findings or scores | NR | Clinical total/effective response | NR | NR | NR |
| H03 | Ding | 2019 | NR | NR | Endoscopic/mucosal findings or scores | NR | Clinical total/effective response | NR | Reported in source text | H. pylori clearance/negative conversion |
| H04 | Du | 2015 | NR | NR | Endoscopic/mucosal findings or scores | NR | Clinical total/effective response | NR | NR | H. pylori clearance/negative conversion |
| H05 | Feng | 2011 | NR | NR | Endoscopic/mucosal findings or scores | NR | Clinical total/effective response | NR | NR | H. pylori clearance/negative conversion |
| H06 | Guo | 2023 | atrophy; dysplasia/IEN; intestinal metaplasia | TCM symptom/syndrome scores | Endoscopic/mucosal findings or scores | G-17/gastrin; MTL/motilin | Clinical total/effective response | NR | Reported in source text | NR |
| H07 | Hu | 2016 | atrophy; dysplasia/IEN; intestinal metaplasia | NR | Endoscopic/mucosal findings or scores | G-17/gastrin | Clinical total/effective response | NR | NR | NR |
| H08 | Hu | 2019 | atrophy; dysplasia/IEN; intestinal metaplasia | NR | Endoscopic/mucosal findings or scores | PGI/PGII/PGR; G-17/gastrin | Clinical total/effective response | IL-1β/IL-6; TNF-α; CRP | Reported in source text | quality of life |
| H09 | Jin | 2012 | NR | NR | Endoscopic/mucosal findings or scores | NR | Clinical total/effective response | NR | NR | NR |
| H10 | Ke | 2025 | atrophy; inflammation/activity; intestinal metaplasia | TCM symptom/syndrome scores | Endoscopic/mucosal findings or scores | NR | NR | NR | NR | anxiety/depression scales; quality of life |
| H11 | Peng | 2022 | NR | NR | NR | NR | Clinical total/effective response | NR | NR | quality of life; H. pylori clearance/negative conversion |
| H12 | Wang | 2012 | NR | NR | Endoscopic/mucosal findings or scores | NR | Clinical total/effective response | Ki-67 / molecular markers | NR | H. pylori clearance/negative conversion |
| H13 | Xiang | 2007 | atrophy; dysplasia/IEN; intestinal metaplasia | NR | Endoscopic/mucosal findings or scores | NR | Clinical total/effective response | NR | NR | quality of life; H. pylori clearance/negative conversion |
| H14 | Xu | 2021 | atrophy; dysplasia/IEN; inflammation/activity; intestinal metaplasia | TCM symptom/syndrome scores | NR | PGI/PGII/PGR; G-17/gastrin | Clinical total/effective response | NR | NR | NR |
| H15 | Ye | 2024 | NR | TCM symptom/syndrome scores | Endoscopic/mucosal findings or scores | NR | Clinical total/effective response | NR | Reported in source text | anxiety/depression scales |
| H16 | Ye | 2020 | atrophy | NR | Endoscopic/mucosal findings or scores | PGI/PGII/PGR; G-17/gastrin | Clinical total/effective response | CD markers | NR | NR |
| H17 | Zhu | 2009 | NR | NR | NR | NR | Clinical total/effective response | IL-1β/IL-6 | NR | NR |
| H18 | Zhu | 2010 | NR | NR | Endoscopic/mucosal findings or scores | NR | Clinical total/effective response | NR | NR | H. pylori clearance/negative conversion |
| H19 | Zhu | 2021 | atrophy | TCM symptom/syndrome scores | NR | NR | Clinical total/effective response | NR | Reported in source text | anxiety/depression scales; quality of life |
| H20 | Zou | 2024 | Global staging improvement (OLGA/OLGIM/dysplasia disappearance); global histological scoring improvement; total pathological score; dysplasia disappearance | NR | Gastroscopy with systematic biopsy follow-up; no separate pure endoscopic efficacy outcome used | NR | NR | NR | Adverse events and serious adverse events reported; safety laboratory monitoring described | NR |
| U02 | Xu | 2026 | atrophy; dysplasia/IEN; inflammation/activity; intestinal metaplasia | TCM symptom/syndrome scores | NR | PGI/PGII/PGR | Clinical total/effective response | VEGF; Ki-67 / molecular markers | NR | NR |
| U04 | Wang | 2026 | atrophy; intestinal metaplasia | TCM symptom/syndrome scores | Endoscopic/mucosal findings or scores | PGI/PGII/PGR; G-17/gastrin | Clinical total/effective response | NR | NR | anxiety/depression scales |

Notes: Study identity follows the verified 69-source crosswalk. S12, S23, S25, S36 and S41 are not included. The historical H-series IDs correspond to source files B1–B24 after author/title/year verification; notably H17 corresponds to B4. U02 corresponds to the source file labelled U03 (Xu Zhangying), whereas U04 corresponds to the source file labelled U02 (Wang Shuo). NR indicates not reported or not reliably recoverable from the supplied full text; no value was invented to replace NR. Chinese ingredients and quantities are transcribed from the source articles, including unusual source-reported values. Botanical/Latin names were editorially standardized from the source-reported Chinese ingredient terms using the 2025 Chinese Pharmacopoeia and accepted taxonomic nomenclature; they were not necessarily printed verbatim in the original articles. Where the Chinese term did not determine a unique species, the genus or 'species not specified' is shown. Animal-, mineral- and fermented-derived materia medica are labelled accordingly. Proprietary products remain NR when the article did not report their ingredient composition. This rule was applied to H20 (Moluodan). For H20, the eligible review comparison is Moluodan plus folic acid versus folic acid alone; other randomized arms are retained only as source context and are not used as eligible review comparisons.
